
JOURNAL INFORMATION
==============================
NLM Title Abbreviation: J Health Pollut
Journal ID: J Health Pollut
NLM Journal ID: 101690849
Journal ID: hapn

Journal of Health & Pollution

EISSN: 2156-9614
Publisher: National Institute of Environmental Health Sciences

ARTICLE INFORMATION
==============================
PMCID: PMC6221503
PMID: 30524807
DOI: 10.5696/2156-9614-6.12.S1
Article version: 1
Subjects: Supplement

Proceedings of the 8th International Conference on Children's Health and the Environment

Publication date: 2016 Dec
Electronic publication date: 2016 Dec 19
Volume: 6
Issue: 12
First page: S1
Last page: S154
Copyright: © 2016 Black Smith Institute
Copyright year: 2016
License: This is an Open Access article distributed in accordance with Creative Commons Attribution License (http://creativecommons.org/licenses/by/3.0/).
License URL: https://creativecommons.org/licenses/by/3.0/

Page count: 154

==============================
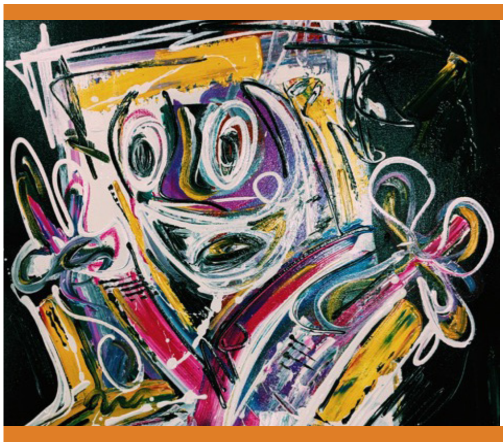

### 8th International Conference on Children's Health and the Environment

### Abstracts

### Conference proceedings

All sessions presented at the 8th International Conference on Children's Health and Environment

14–16 September 2016

Parc de Recerca Biomèdica de Barcelona – PRBB
Doctor Aiguader, 88 (at ISGlobal), Barcelona

Presented by

**INCHES**

(International Network on Children's Health and Safety)

J|H&P
JOURNAL OF HEALTH AND POLLUTION

### The 8th INCHES (International Network on Children's Health, Environment and Safety) Conference

*Barcelona • 14–16 September, 2016*

### Overview

Children, like all humans, are under the influence of multiple environmental stressors. However, children are not little adults. Their organs and metabolism are under formation and their capacity to face pollutant stress is different. Exposure to deleterious compounds starts in the preconceptional and fetal stage. In the first years of life, children are affected by exposure to toxic chemical products present in air, water, soil, domestic dust, toys, clothes, furniture and diet. The nature of pollution is changing. In rapidly developing countries, urbanization, climate change, increasing motor vehicle use and the wide spread of toxic chemicals use, pesticides and polluting industries, levels of ambient and indoor biomass air pollution and soil pollution are sharply increasing the exposure for the most vulnerable. Moreover, some polluting agents are incorporated together with nutrients in the same ingestion processes, e.g. during breastfeeding. Some food items such as fish, the main source of omega 3 fatty acids, also carry pollutants such as organochlorine compounds and methylmercury.

The health effects of these toxic compounds may not be noticed directly or shortly after exposure. Damage in growing organisms may result in delayed disorders later in life, e.g. during adolescence, the reproductive period or maturity. The effects of pollutants on children's health also depend on individual susceptibility. A full understanding of health impacts requires studies integrating gene-environment interactions. The details will provide more and more proof that we need to protect children.

### Summary of overall organization-wide achieved results

The International Network on Children's Health, Environment and Safety (INCHES) (http://www.inchesnetwork.net) was founded at a workshop meeting before the 1st International Conference on Children's Environmental Health, August 1998 in Amsterdam, the Netherlands, where the WHO, European Environmental Agency (EEA), US Environmental Protection Agency (EPA) and other organisations had an active participation, stressing the growing importance of CEH as a global health issue. The WHO and INCHES have been active partners the last 16 years.

### The Organisation: International Network on Children's Health, Environment and Safety (INCHES)

The International Network on Children's Health, Environment and Safety (INCHES) is a global network with the following goals:

1) increase understanding of and accord on how environmental factors influence child health;
2) create a global dissemination of research and information on children's environmental health;
3) educate and facilitate information exchange on the best practices and policies in children's environmental health;
4) identify information gaps and stimulate to new research; and
5) advocate for children's environmental health in the intergovernmental arena.

*Some highlights of INCHES activities since its formation in 1998 include:*

- There are now about 150 organizational members, some of which are well structured, established and active as national NGOs interested in an intensified international cooperation.
- More than 30,000 individual members (most of them health professionals) within these member organizations.
- Strong contacts in several countries where national children's environmental health networks have been founded or are under creation.
- Excellent international contact in the field of children, environment and health in the intergovernmental, governmental and NGO circles.
- Some official contacts in the field (relations with WHO, EEA, membership in European Health and Environment Alliance network, International Society of Doctors for the Environment).
- Several members in the medical scientific community covering most aspects of environmental health able and ready to give foundation to the content of INCHES' work.
- Training programs in cooperation with several universities in some countries have been conducted as a project.
- INCHES initiated several projects funded by the European Union.
- INCHES organized or co-organized several conferences on children's environmental health over the last two decades.
- INCHES collaborates with similar networks in North America: the Children's Environmental Health Network, International Society for Children's Health and the Environment and the Canadian Institute of Child Health.

Many topics were addressed in the 8th International Conference on Children's Environment and Health, which was organized by INCHES on 14-16th September 2016 in Barcelona. The Conference is organized by the International Network on Children's Health, Environment and Safety (INCHES) and the Institute of Environmental Assessment and Water Research (IDAEA) and Spanish Council for Scientific Research (CSIC). The meeting provided an international forum for the latest research findings in children's environmental health. Specific attention was also devoted to identifying opportunities to minimize childhood prenatal and postnatal exposure to environmental contaminants, to provide insight in the field of science and policy interface and to develop a greater awareness among health professionals about children's health. The abstracts that are published in the present issue summarize the main research efforts reported at this meeting.

### The objectives of the conference:

- Provide an international forum for the latest research findings in children's environmental health;
- Define the relationship between environmental contaminants and children's health;
- Strengthen the importance of prenatal exposure to environmental factors on pregnancy outcome and children's health;

- Identify opportunities to minimize childhood prenatal and postnatal exposure to environmental contaminants;
- Provide insight in the activities in the field of science and policy interface;
- Build a platform of knowledge at an international level;
- Develop a greater awareness among health professionals about children's health, environment and safety;
- Initiate future directions and international collaborations in research in the field of children's environmental health.

### The Scientific Program

The scientific program of the 8th INCHES Conference includes about 140 lectures, short oral presentations and posters presented by scientists from 34 countries covering a broad spectrum of topics. Abstracts of these presentations appear in this issue of the journal. The topics are related to: Health effects of pollutants in children (pesticides, heavy metals, air pollution, tobacco) prenatal exposures, reproductive health, cancer, obesity, biomonitoring, Children Environmental Health Centres and Specialty Units, children's environmental rights, climate change, sustainable development, e-waste, radiology, safety and injury prevention, training for pediatricians and health policy regarding Children's Environmental Health.

**The following is a list of abstracts presented at the 8th INCHES (International Network of Children's Health, Environment and Safety) Conference held on September 14-16, 2016 in Barcelona, Spain. Abstracts are presented in alphabetical order by corresponding author last name.**

**Neither the conference organizers or the Journal of Health and Pollution can be held responsible for inaccuracies or errors in any included abstracts.**

### High Risk for Neural Tube Defects from Arsenic in Drinking Water and Rice in Asia

Yona Amitai (1,2), Gideon Koren (3)

(1) Department of Management, Bar Ilan University, Ramat Gan, Israel
(2) Meuhedet Health Services, Jerusalem, Israel
(3) Faculty of Pharmacy, University of Toronto, Toronto, Canada

Corresponding author:
Amitai, Yona
Ramat Gan, Israel
yonaamitai89@gmail.com

*Background*

Neural tube defects (NTDs) affect >300,000 children annually worldwide. The incidence of NTDs in Northern India (7.7/1000) is tenfold higher than in the US (0.7/1000). Higher rates were previously reported in Northern China. The causes of these trends have not been elucidated. Arsenic is a teratogen shown in animals to induce NTDs. In India and China, the main potential sources for environmental arsenic exposure are groundwater and rice as a staple food.

*Methods*

Analyzing the association between high environmental arsenic exposure through drinking water and rice with high NTDs rates in these regions.

*Results*

Literature review indicates that arsenic contamination of groundwater is the main source of environmental arsenic exposure. The locations of toxic arsenic regions in China and India correspond in most cases to the northern regions where the NTDs rates are high. Rice, the staple food in India and China, can absorb up to 10 times more arsenic than other crops such as wheat and might further increase arsenic exposure.

*Conclusions*

We hypothesize that this NTD-arsenic in drinking water and rice association may explain why these areas in the northern regions of both countries have the highest incidence of NTDs. If proven true, this has major public health implications.

### Effect of Diet on Allergies Among Preschool Children

Sandra Andrusaityte, Regina Grazuleviciene

Department of Environmental Sciences, Vytauto Didžiojo Universitetas, Kaunas, Lithuania

Corresponding author:
Andrusaityte, Sandra
Kaunas, Lithuania
s.andrusaityte@gmf.vdu.lt

*Background*

The prevalence of asthma and allergy has increased among children. This increase in prevalence might be related to diet. The present epidemiological study investigated the relationship between fruits, vegetables and nuts intake and the prevalence of wheezing ever, asthma, and eczema among preschool children.

*Methods*

This nested case-control study included 1,489 children of 4–6 years of age residing in Kaunas, Lithuania, who were recruited in 2007-2009 to the Kaunas Cohort Newborns study. Responses to the questionnaire completed by parents were used to collect information on allergic diseases, diet and other factors. Association between diet and children's allergic diseases was estimated by multivariable regression, controlling for covariates. Ethical approval was granted by the Lithuanian Bioethics Committee.

*Results*

We found that 83.3% of all children ate fresh fruits (62.7% - vegetables and 6.2% - nuts) at least three times per week. A significantly lower prevalence of wheezing was found among children who ate fruits at least three times per week than among those did not eat fruits (crude odds ratio: 0.44, 95% confidence interval (CI) 0.22 to 0.87). Adjusted odds ratios (aOR) of asthma were also lower for children who ate vegetables at least three times per week compared with no vegetable consumption (aOR 0.70, 95% CI 0.37 to 1.35). Consumption of nuts 1-2 times per week was associated with lower prevalence of childhood asthma (aOR 0.69, 95% CI 0.44 to 1.08) and eczema (aOR 0.56 95% CI 0.35 to 0.89).

*Conclusions*

The results indicated beneficial effects of frequent consumption of fresh fruit and vegetables on asthma and allergies in children. These results might have important implications for children's health.

### Prenatal Mercury Exposure and Fetal Development: Results from Infancia y Medio Ambiente Cohort

Ferran Ballester (1,2,3), Carmen Iñiguez (1,2), Mario Murcia (1,2), Ana Fernández-Somoano (2,4), Mònica Guxens (2,6,9), Mikel Basterrechea (2,5,7), Aitana Lertxundi (2,8), Adonina Tardon (2,4), Jordi Sunyer (2,6), Sabrina Llop (1,2)

(1) Epidemiology and Environmental Health Joint Research Unit, FISABIO-Universitat Jaume I-Universitat de València, Valencia, Spain
(2) Spanish Consortium for Research on Epidemiology and Public Health, Madrid, Spain
(3) Nursing School, Universitat de València, València, Spain
(4) Universidad de Oviedo, Oviedo, Spain
(5) Subdirección de Salud Pública y Adicciones, Gipuzkoa, Spain
(6) ISGlobal, Centre for Research on Environmental Epidemiology, Barcelona, Catalonia, Spain
(7) Health Research Institute, San Sebastián, Spain
(8) Universidad del País Vasco, Spain
(9) Department of Child and Adolescent Psychiatry/Psychology, Erasmus University Medical Centre–Sophia Children's Hospital, Rotterdam, the Netherlands

Corresponding author:
Ballester, Ferran
Valencia, Spain
ballester_fer@gva.es

### *Background*
Prenatal mercury exposure has been related to reductions in anthropometry at birth. Levels of mercury have been reported to be relatively high in the Spanish population, including vulnerable groups such as pregnant women and newborns. Our objective is to investigate the relationship between prenatal exposure to mercury, expressed as cord blood mercury levels, and fetal biometry in the Spanish Infancia y Medio Ambiente study.

### *Methods*
The study subjects were 1867 pregnant women and their newborns participating in the Infancia y Medio Ambiente Spanish birth cohort study. Biparietal diamete, femur length, abdominal circumference and estimated fetal weight were measured by ultrasounds at 12, 20 and 34 weeks of gestation. Size and growth between these points were assessed by standard deviation scores adjusted for constitutional characteristics. Total mercury was determined in cord blood. Associations were investigated by linear regression models, adjusted by socio-demographic, nutritional – including four fish groups – and lifestyle-related variables in each area. Final estimates were obtained using meta-analysis. Ethical approval was granted by the ethical commitees of the participating health centers.

*Results*

Arithmetic and geometric means of total mercury were 11.0 and 8.2 μg/L, respectively. All the estimates of the association between prenatal mercury and growth from 0 to 12 weeks showed reductions in z-scores, and were only statistically significant for BPD. A doubling of cord blood T-Hg was associated with a slight reduction in size of biparietal diameter at week 12. Conversely, coefficients were positive (but none significant) for growth from 12 to 20 weeks. Finally, results for growth from 20 to 34 weeks again showed negative estimates for all indicators except femur length.

*Conclusions*

This study suggests that mercury exposure is associated with changes in fetal growth at different periods of pregnancy. Further research is needed to confirm this. Due to this and other potential hazards, preventive actions should be oriented towards reducing exposure during pregnancy.

### Maternal Cell Phone Use During Pregnancy and Child Behavior Problems In Five Birth Cohorts

Laura Birks (1,2,3), Mònica Guxens (1,2,3,4), Eleni Papadopoulou (5), Jan Alexander (5), Mina Ha (6), Anke Huss (7), Leeka Kheifets (8), Hyungryul Lim (6), Jorn Olsen (9), Madhuri Sudan (8,9), Roel Vermeulen (7,10), Elisabeth Cardis (1,2,3), Martine Vrijheid (1,2,3)

(1) Barcelona Institute for Global Health, Centre for Research in Environmental Epidemiology, Barcelona, Spain
(2) Pompeu Fabra University, Barcelona, Spain
(3) Centro de Investigación Biomédica en Red de Epidemiología y Salud Pública, Instituto de Salud Carlos III, Madrid, Spain
(4) Department of Child and Adolescent Psychiatry/Psychology, Erasmus University Medical Centre–Sophia Children's Hospital, Rotterdam, the Netherlands
(5) Domain of infection control and environmental health, Norwegian Institute of Public Health, Oslo, Norway
(6) Department of Preventive Medicine, Dankook University, College of Medicine, Cheonan, Korea
(7) Institute for Risk Assessment Sciences, Utrecht University, Utrecht, the Netherlands
(8) Department of Epidemiology, School of Public Health, University of California, Los Angeles, California, USA
(9) Danish Epidemiology Science Centre, Department of Public Health, Aarhus University, Aarhus, Denmark
(10) Julius Center for Health Sciences and Primary Care, University Medical Center, Utrecht, the Netherlands

Corresponding author:
Birks, Laura
Barcelona, Spain
laura.birks@isglobal.org

*Background*

Previous studies regarding maternal cell phone use during pregnancy collected retrospectively and associations with child behavior problems at 7 years were not conclusive. Therefore, we evaluated this association with data from the previous studies together with prospective data from three other birth cohorts.

### Methods

We used individual participant data from 84,077 mother-child pairs in five population-based birth cohorts in Denmark (1996-2002), Korea (2006-2011), the Netherlands (2003-2004), Norway (1999-2008), and Spain (2003-2008). We categorized cell phone use based on maternal-reported frequency of calls during pregnancy: none, low, medium, and high. Behavior problems (reported by mothers using CBCL or SDQ) were classified in the borderline/clinical and clinical ranges using validated cut-offs in children aged 5-7. Cohort specific risk estimates were meta-analyzed.

### Results

In all cohorts, 7% of mothers reported no cell phone use during pregnancy, except in Denmark where 61% did not use cell phones. For all cohorts, 6% of mothers reported high cell phone use. Mothers who reported no cell phone use were less likely to have a child with overall behavior problems compared to those who reported low use (odds ratio (OR) 0.82, 95% confidence interval (CI) 0.75, 0.89). High cell phone use during pregnancy was associated with an increased risk for inattention/hyperactivity problems compared to low cell phone use during pregnancy (OR 1.28, 95% CI 1.12, 1.48). No clear associations were observed in relation to the other behavior problems.

### Conclusions

Our results indicate that high cell phone use during pregnancy may be associated with hyperactivity problems and no cell phone use may be associated with a decreased risk in any behavior problems among children aged 5-7. While this is the largest study to evaluate this association, these results should be interpreted with caution, as there is possible residual confounding that may influence both cell phone use and child behavior.

---

### The Role of Trace Elements, Antioxidants and Oxidative Stress in Very Low Birth Weight Infants

Beatrice Bocca (1), Simona Ciccarelli (2), Rocco Agostino (3), Alessandro Alimonti (1)

(1) Department of Environment and Health, Istituto Superiore di Sanità, Rome, Italy
(2) Neonatal Intensive Care Unit and Neonatal Pathology, St. John Calibita-Fatebenefratelli Hospital, Rome, Italy
(3) Member of Ethics Committee, Bambino Gesù Pediatric Hospital, Rome, Italy

Corresponding author:
Bocca, Beatrice
Rome, Italy
Beatrice.bocca@iss.it

### Background

Reference data on trace elements concentration, antioxidants and oxidative status in very low birth weight infants are limited and need updating for use in clinical settings.

*Methods*
Thirteen very low birth weight infants whose mean birth weight and gestation were 1,167 g and 33.2 weeks, respectively, were enrolled in this study. Cadmium (Cd), copper (Cu), iron (Fe), mercury (Hg), manganese (Mn), selenium (Se) and zinc (Zn) were determined in serum and urine of infants at two points in the early postnatal period (24th day and 38th day). Infants were also analyzed for oxidative status and antioxidant enzyme activities determining different indicators: serum catalase; serum Cu, Zn, Fe, Mn-superoxide dismutase; urine nitrate/nitrite; serum total oxidative status (sum of lipo- and hydro-peroxides); and serum total antioxidant capacity (sum of thiols, proteins, bilirubin, uric acid, β beta-carotene, ascorbic acid, vitamin E, etc.).

*Results*
A higher urinary excretion of Cu, Se and Zn was observed at the 24th day of life than at the 38th day; in serum, Zn slightly decreased after two weeks from the enrollment. These results indicated that losses of elements and incomplete body stores are more pronounced in the earlier stage of life (24th day) than later on. Moreover, an increase in urine Cd concentrations was found at the 38th day over at enrollment, but values of this element were below the safe threshold for toxic effects. Infants had significant lower catalase activity at the 38th day than at the 24th day of life and a reduction of total antioxidant barriers. Also, high values of oxidative stress were observed in very low birth weight infants, indicative of stimulated formation of lipid and oxygen radicals during the first 5 weeks of age. Higher concentrations of trace elements, lower antioxidant barriers and higher levels of oxidative stress were found in very low birth weight infants.

*Conclusions*
Early diagnosis of essential and toxic elements and oxidant/antioxidant status in very low birth weight infants is essential for contributing to avoid severe postnatal health consequences in these patients and it is a key result in order to balance from the diet and/or by micronutrient supplementation the impact of free radicals.

---

### Urine Total Arsenic and Arsenic Species in Children from Northern Italy

Beatrice Bocca (1), Anna Pino (1), Jenny D'Aversa (1), Alberto Gotti (2,3), Dimosthenis Sarigiannis (2,3), Liza Vecchi Brumatti (4), Marika Mariuz (4), Fabio Barbone (4), Gemma Calamandrei (1), Alessandro Alimonti (1)

(1) Istituto Superiore di Sanità, Rome, Italy
(2) Aristotle University of Thessaloniki, Thessaloniki, Greece
(3) Environmental Health Engineering, Scuola Universitaria Superiore IUSS, Pavia, Italy
(4) Institute for Maternal and Child Health, Istituto Di Ricovero e Cura a Carattere Scientifico Burlo Garofolo, Trieste, Italy

Trieste, Italy
Corresponding author
Bocca, Beatrice
Rome, Italy
Beatrice.bocca@iss.it

### *Background*

Arsenic (As) exposure during childhood has been associated with neurobehavioral effects in population-based cohort studies. In addition, variations in As on children health outcomes may be, to some extent, related to exposure to different As species.

### *Methods*

Within the Cross-Mediterranean Environment and Health Network-Life project, aimed at assessing the health risk due to exposure to neurotoxic chemicals, a cohort of 200 children at 7 years (the Northern Adriatic Cohort II Public Health Impact of Long-term, Low-level Mixed Element Exposure in Susceptible Population Strata cohort) from the coastal area of Friuli Venezia Giulia Region (Italy) was analyzed for As exposure. Total As concentration was determined in children's urine by high resolution inductively coupled plasma mass spectroscopy. Then, the concentrations and distributions of urinary As species, including inorganic arsenic (iAs) [arsenite (iAsIII) and arsenate (iAsV)], monomethylarsonic acid (MMA), dimethylarsinic acid (DMA), arsenobetaine (AsBet) and arsenocholine (AsChol), were determined by high-performance liquid chromatography combined with inductively coupled plasma mass spectrometry. Ethical approval was granted by the Cross-Mediterranean Environment and Health Network-Life.

### *Results*

Results indicated a good agreement between the values obtained for total As (median, 10.6 μg/L) and the sum of species. The order of As species excretion in urine of children was: AsBet > DMA > MMA □ iAsIII > iAsV (AsChol was at trace levels); moreover, the second methylation step was more active than the first one (DMA > MMA).

### *Conclusions*

In conclusion, in children, inorganic arsenics (AsIII and AsV) were metabolized to the less toxic forms as DMA and MMA through the methylation process. In addition, a very high percentage of urine As was present as AsBet which is essentially harmless and suggestive of dietary As. Due to the different toxicity of As compounds, speciation of As in urine was confirmed to be more convenient for health risk assessment than measuring total As concentration and it can give valuable information about the metabolism of As species within children's bodies.

### Multi Residue and Non-Target Screening for Characterization of Organic Chemicals in Indoor Dust

Petra Booij, Garry Codling, Alin Ionas, Jana Klánová

Research Centre for Toxic Compounds in the Environment (RECETOX), Masaryk University, Faculty of Science, Brno, Czech Republic

Corresponding author:
Booij, Petra
Brno, Czech Republic
booij@recetox.muni.cz

*Background*

The indoor environment can expose us to a concentrated cocktail of chemicals. Indoor dust may be a source or sink for a range of contaminants. Most studies to date have focused on individual compounds or classes of contaminants. A review on dust indicated that a large number of different chemicals have been identified in recent years, e.g. polybrominated diphenyl ethers, pesticides, phthalates, polycyclic aromatic hydrocarbons, polychlorinated biphenyls, and perfluorinated compounds. Polybrominated diphenyl ethers congener concentrations in serum and feces of toddlers were significantly correlated to those in house dust and suggest that dust exposure plays a larger role in the polybrominated diphenyl ethers body burden in toddlers than in their mothers, and this may be related to additional ingestion of dust as toddlers are apt to place toys and hands in the mouth, thus gaining additional exposure.
An understanding of the hazards that contaminants in dust may pose to human health, a more comprehensive non-target approach using state of the art instruments will allow the identification of legacy compounds, but also identify potentially hazardous compounds not traditionally screened for.

*Methods*

We developed a targeted multi-residue screening method to analyse a diverse array of compounds such as may be found in indoor dust and human blood. In combination with non-target screening, previously unreported compounds in dust can be tentatively identified. Methods for screening blood and dust were tested using a mixture of >200 organic contaminants. By using different ionisation sources in positive and negative mode, identification of the introduced compounds was validated and different extraction procedures tested. The aim was primarily to understand the limitations of traditional extraction techniques and to see the differences that the extraction procedures has on the screening approach.

*Results*

Using the Norman recommended extraction technique for household dust and a trial of 5 typical blood extraction techniques we identified the recovery efficiency of a wide variety of organic contaminants in dust and blood. Using different ionisation sources, we further determined electrospray in positive, combined with negative mode, was able to detect around 80% of all compounds analyzed, however other ionisation sources identify unique features. Hydrophilic-lipophilic balance extraction cartridges, out of 5 extraction methods, extracted compounds with the highest recovery in blood, although other similar solid-phase extraction methods were also effective.

*Conclusions*

A single extraction technique for blood and the NORMAN non-target method for dust may be applicable for the determination of a wide range of compounds. For non-target analysis, electrospray ionization in positive and negative is necessary for the broadest range of most organic contaminants measured, however other ionization sources provide unique features that must be considered for a comprehensive approach.

### Lead Intoxicated Children in Kabwe/Zambia

Stephan Böse-O'Reilly (1,2), John Yabe (3), Joseph Makumba (4), Jack Caravanos (5,6)

(1) University Hospital of LMU Munich, Department of Occupational, Social and Environmental Medicine, WHO Collaborating Centre for Occupational Health, Munich, Germany
(2) Department of Public Health, Health Services Research and Health Technology Assessment, UMIT – University for Health Sciences, Medical Informatics and Technology, Hall in Tirol, Austria
(3) University of Zambia, School of Veterinary Medicine, Lusaka, Zambia
(4) Misenge Environmental and Technical Services Limited, ZCCM Investment Holdings, Kitwe, Zambia
(5) City University of New York School of Public Health, New York, USA
(6) Pure Earth / Blacksmith Institute, New York, USA

Corresponding Author:
Böse-O'Reilly, Stephan
Munich, Germany
Stephan.boeseoreilly@med.uni-muenchen.de

*Background*

Kabwe was an important lead and zinc mining town in Zambia for over 90 years, which left waste and lead contaminated tailings behind. Lead is a well known serious health hazard, causing anemia, seizures, encephalopathy and death. Soil in Kabwe's housing areas is highly contaminated with lead. Children from neighboring townships are especially vulnerable by ingesting toxic lead dust.

*Methods*

The environmental assessments and health data for Kabwe's children were collected and analyzed. Data is available from three different sources: Copperbelt Environment Project; Pure Earth and University of Zambia; and Hokkaido University in Sapporo, Japan. Ethical approval was granted by the respective national authorities in Zambia.

*Results*

In most housing areas the tolerable soil lead levels of 400 ppm was surpassed. Children's blood lead levels were increased (≥ 10 µg/dL) in the most affected townships and in over 95% of children. Most children did have blood lead levels requiring urgent action to reduce blood lead levels. Exposure to toxins urgently needs to be reduced. At over ≥ 45 µg/dL, medical treatment is recommended, and in the most affected townships > 50% of the children showed such high levels. The existing data clearly prove the severity of lead exposure in Kabwe.

*Conclusions*

Large proportions of the children are not only highly exposed, but actually have high to extremely high levels of lead in their bodies. According to threshold levels and international recommendation, they need to be treated and their exposure must be considerably reduced.

Funding by the World Bank (Environmental Remediation and Improvement Project (P154683).

### When Parents, NGOs, Environmental NGOs and Experts Join Forces to Spur Actions to Protect Children's Environmental Health

Djohra Azzi (1), Danielle van Kalmthout (2), Valérie Xhonneux (3), Erik Grietens (4) and Catherine Bouland (1)

(1) Université Libre de Bruxelles (ULB), School of Public Health, Research Centre on Environmental and Occupational Health, Brussels, Belgium
(2) Gezinsbond, Brussels, Belgium (Parent Association in Flanders)
(3) Fédération Inter-Environnement Wallonie, Namur, Belgium (Environmental Association in Wallonia)
(4) Bond Beter Leefmilieu Vlaanderen, Brussels, Belgium (Environmental Association in Flanders)

Corresponding author:
Bouland, Catherine
Brussels, Belgium
catherine.bouland@ulb.ac.be

*Background*

Involved in the international discussions on environmental health since 1994, Belgium developed its National Action Plan on the Environment and Health (NEHAP) in 2003 and launched coordinated actions. With the 4th WHO conference on environmental health in Europe, the focus was put on children. Most European states launched a specific Children, Environment and Health Action Plan (CEHAP), and Belgium decided to foster specific actions towards children within their existing NEHAP. The mid-term review in 2007 indicated a demand from parents and environmental associations to be involved. Since 2010 and the ending of the European Action Plan on Environment and Health, activities have been severely reduced.

*Methods*

A review of the actions taken is matched to demands from NGOs (parent's organisations and environmental associations). Ideas were collected around 3 main priorities among members of civil society and academia working together as the platform "Childproof".

*Results*

NEHAP actions were focused on capacity building in nurseries, research on children and cancer to integrate environmental information, and on human bio-monitoring, including towards children. The first ideas collected by the parents and environmental associations focus on chemicals and endocrine disruption, air quality, outdoor and indoor noise and inequalities. They will be discussed and presented to the NEHAP secretariat to spur specific activities and policies for the health of actual and future children.

*Conclusions*

Knowledge is growing and uncertainties remain. Hence, actions must be taken to enable children to grow and develop in good health. The whole society has a role to play to reduce children's exposure to environmental hazards and work together to encourage the implementation of laws and policies to protect children from known and potential environmental hazards.

### Organohalogenated Pollutants in Argentinean Postpartum Women Living In Salta and Ushuaia

Natalia Bravo (1), Solrunn Hansen (2), Inger Økland (3), Marisa Viviana Álvarez (4), Silvina Matiocevich (5), Jon-Øyvind Odland (2), Joan O. Grimalt (1)

(1) Institute of Environmental Assessment and Water Research, Spanish National Research Council, Barcelona, Spain
(2) The Artic University of Norway, Trømso, Norway
(3) Stavanger University Hospital, Stavanger, Norway
(4) Hospital Público Materno Infantil de Salta, Salta, Argentina
(5) Clínica San Jorge, Ushuaia, Argentina

Corresponding author:
Bravo, Natalia
Barcelona, Spain
natalia.bravo@idaea.csic.es

*Background*

Organohalogenated pollutants, namely organochlorine pesticides, polychlorobiphenyls (PCBs) and polybromodiphenyl ethers (PBDEs), may undergo long-range atmospheric transport because they are semi-volatile and chemically stable. They accumulate in organisms and humans as consequence of their lipophilic character. The toxic properties of most of them generate deleterious health effects in both humans and wild species. These compounds have been mostly synthesized and used in the northern hemisphere, but their strong capacity for long-range atmospheric transport has led to a global planetary distribution, including the southern hemisphere. While there is information on the occurrence of these compounds in the ecosystems of this hemisphere, the degree of information on the accumulation patterns of these compounds in southern hemisphere human populations is very limited.

*Method*

The present study was devoted to filling this gap by analysis of maternal serum from Argentina, representing postpartum mothers from the city of Salta (25°S; n = 498) and Ushuaia (55°S; n = 200). These samples were collected from April 2011 to March 2012 between the first and third days after delivery. Ethical approval was granted by the Ethics Committee at the Board of Medical School of Salta and conducted in accordance with the Helsinki declaration. The Northern Norway Regional Board of Research Ethics approved the study along with the Norwegian EMASAR biobank, containing the Argentinean blood samples.

*Results*

The median concentrations of the most abundant compounds were 0.33 and 0.51 ng/mL of total DDTs, 0.20 and 0.18 ng/mL of polychlorinated biphenyl, and 0.067 and 0.043 ng/mL of hexachlorobenzene in Ushuaia and Salta, respectively. These concentrations were generally low, but lower concentrations have been reported for various compounds in some northern hemisphere locations such as Norway. The influence of maternal age, parity, and body mass index on the concentrations of these pollutants has been investigated.

### *Conclusions*

This study reports the concentrations of persistent organic pollutants (POPs) in two representative populations of postpartum women living in Ushuaia and Salta, Argentina. The levels of POPs have been linked with age, BMI and parity, showing that while they increase with age and BMI they also decrease with parity. Although PBDEs decrease with the age of women, there are significant differences ($p<0.001$) between the concentrations found in Ushuaia and Salta, showing a latitudinal dependence, a possible consequence of long-range transport and condensation.

---

### E-Waste and Child Health – WHO Global Initiative

**Marie-Noël Bruné Drisse, Gloria Chen**

**Department of Public Health, Environment and Social Determinants of Health**
**World Health Organization, Geneva, Switzerland**

**Corresponding author:**
**Bruné Drisse, Marie-Noël**
**Geneva, Switzerland**
**brunedrissem@who.int**

E-waste is a growing environmental concern to children's health, with 20-50 million tons created annually and expected to increase by 19% between 2014 and 2018. Much of it is shipped to low and middle income countries, where valuable parts are extracted by burning and with acid, often informally by children, exposing them to chemicals and toxicants. This can occur in e-waste recycling hotspots, where entire towns are built around e-waste operations, as seen in Ghana and China, or throughout towns and cities as reported in a recent Latin-America e-waste report by United Nations agencies. When e-waste recycling is not concentrated in a particular area, garbage pickers may collect e-waste from landfills or door to door, before bringing it home to take it apart and burn. In both of these informal sector types, modern industrial processes and worker protection are lacking, while child labor goes unseen.
A systematic review led by WHO and WHO collaborating systems found that exposure to e-waste was associated with spontaneous abortion, stillbirth, premature birth, reduced birth weight and birth length, and DNA damage. Chemical exposures from e-waste, including lead, mercury, cadmium, PCBs and dioxins are known to adversely affect neurodevelopment, even at lower levels. Thus, it is urgent to protect the health and reduce exposures of vulnerable groups of children, pregnant women, and workers in the informal sector against e-waste exposures.

The health effects of e-waste are not widely known. Informal workers lack protective equipment and occupational health services. Children are at risk from work in recycling and exposures in their home and living in the areas contaminated by informal recycling. The health sector is often not aware of the dangers of e-waste. WHO, WHO collaborating centres and collaborators are working on advocating for this issue and on identifying and promoting e-waste interventions that will improve child health, including communicating the problem to health actors, developing training methods and tools for health professionals, and creating community multisectoral teams to identify local needs to protect health.

### How Can the Increase in Childhood Cancer be Explained? The Role of Transplacental and Transgenerational Carcinogenesis

Ernesto Burgio (1,2,3)

(1) European Cancer and Environment Research Institute, Brussels, Belgium
(2) International Society of Doctors for Environment, Arezzo, Italy
(3) Commissione Ambiente e Salute, Federazione Italiana Medici Pediatri, Rome, Italy

Corresponding author:
Burgio, Ernesto
Arezzo, Italy
erburg@libero.it
erburg@gmail.com

*Background*

Cancer is generally associated with old age and the explanation most clinicians (and others) advance for its continuous increase, observed throughout the 20th century in all industrialized countries, is that it results from the progressive accumulation over the time of aging of genetic oxidative/stochastic damages with an additional role for the improvement of diagnostic capacities. It is too often forgotten that this increase concerns persons of all ages and, particularly in the last decades, the youth.

*Methods*

Through literature review, the aim of this study is to reconsider, in the light of new epigenetic models of environmental carcinogenesis and of transgenerational cancer transmission, the recent epidemiological data that confirm a substantial increase in childhood cancer throughout Europe, hardly explainable by the current stochastic mutational paradigm.

*Results*

Carcinogenesis is a long and complex process. As far as the increase in childhood cancer is concerned, the key factor for its occurrence should be looked for in parents (exposure of the reproductive cells) or the fetus (exposure in the very first stages of ontogenetic development).

*Conclusions*

The two main possibilities that have to be considered are: 1) direct exposure of the embryo/fetus to physical agents or, through transplacental transmission, to biological (viruses) or chemical agents, capable of directly damaging the fetal DNA or of inducing epigenetic changes in the fetal tissues (fetal programming); 2) transgenerational transmission of epigenetic "signatures" through the gametes. Admitting the importance of such mechanisms would have several implications. The first would be recognizing the highly underestimated role of environmental pollution in the genesis and the progressive increase of cancer. In particular, by hypothesizing that the "initiating" stage of cancer might take place much earlier, in the fetus or even in the parents' gametes, and that the increasing trends in cancer in the very early childhood should be seen as a sentinel sign of a possible transgenerational amplification of (epi)genetic/programmatic changes with their associated pathologies.

### Transgenerational Effects of Treatment with Antiepileptics in Pregnancy: Behavioural and Molecular Markers in a Mouse Study

Anna Maria Tartaglione (1,2), Laura Ricceri (1), Benedetta Perrone (1), Alessia De Felice (1), Emanuela Balestrieri (3), Paola Sinibaldi Vallebona (3), Gemma Calamandrei (1)

(1) Department of Cell Biology and Neuroscience, Istituto Superiore di Sanità, Rome, Italy
(2) Department of Neurology and Psychiatry, Sapienza University of Rome, Rome, Italy
(3) Department of Experimental Medicine and Surgery, University of Rome Tor Vergata, Rome, Italy

Corresponding author:
Calamandrei, Gemma
Rome, Italy
gemma.calamandrei@iss.it

*Background*

Clinical evidence indicates that exposure to the antiepileptic agent valproic acid (VPA) during pregnancy increases the risk of congenital malformations and neurodevelopment delay in children. The mechanisms by which VPA causes neurotoxicity are still unknown, but epigenetic effects are likely implicated.

*Methods*

We used a rodent model to evaluate the transgenerational impact of fetal exposure to VPA on neurobehavioural development of laboratory mice, by studying F1 and F2 generations after a single injection of VPA during pregnancy.

On gestational day 10.5, the F0 pregnant mice were injected subcutaneously with VPA 500 mg/kg of body weight. Offspring of both sexes from the vehicle control and VPA-treated litters were assessed for motor and somatic growth, spontaneous locomotion, ultrasonic vocalization, and nest-odour recognition from postnatal day 4 to 12. Motor activity, social abilities and cognitive functions were also analyzed at the juvenile and adult stage. At adulthood male and female mice of the F1 generation were mated creating three experimental groups of F2 (maternal VPA/paternal vehicle, maternal vehicle/paternal VPA, maternal vehicle/paternal vehicle) to evaluate the contribution of VPA parental exposure to multigenerational effects on the behavioural phenotype. In F2 offspring, the same F1 neurobehavioral assessment was performed. By PCR, we also analysed molecular markers of VPA effects on early brain development in both F1 and F2 generation.

*Results*

In F1, VPA offspring presented tail malformation ("kinky tail"), delay in somatic growth and motor development, hyperactivity and a reduced number of ultrasonic vocalizations. No prenatal treatment-induced differences were evident in adult performance. Also in F2, alterations in early motor development were observed in VPA offspring.

### Conclusions

These findings show that in outbred mice VPA exposure has transgenerational actions on early neonatal behavioural patterns; interestingly, preliminary data suggest that the behavioural changes induced by VPA are paralleled by increased expression of endogenous retroviruses and neuroinflammatory biomarkers, which are possible downstream effectors of the VPA-induced epigenetic alterations.

### Funding Acknowledgments

Supported by FP7 HEALS Grant N° 603946 “Health and Environment-wide Associations Based on Large Population Surveys”, and by Ministry of Health-ISS Project 13CAL “Identification of early markers of pathology in rodent models of autism: role of endogenous endoretroviruses.”

---

### The Northern Adriatic Cohort II in the Friuli Venezia Giulia Region: Prenatal and Childhood Exposure to Metals and Neuropsychological Maturation at 7 Years

Gemma Calamandrei (1), Flavia Chiarotti (1), Fiorino Mirabella (1), Aldina Venerosi (1), Anna Pino (1), Beatrice Bocca (1), Alessandro Alimonti (1), Luca Ronfani (2), Liza Vecchi Brumatti (2), Marika Mariuz (3), Valentina Rosolen (4), Fabio Barbone (4,5)

(1) Department of Cell Biology and Neuroscience, Istituto Superiore di Sanità, Rome, Italy
(2) Institute for Maternal and Child Health, Istituto Di Ricovero e Cura a Carattere Scientifico, Burlo Garofolo, Trieste, Italy
(3)Department of Medical and Biological Sciences, University of Udine, Friuli Venezia Giulia Region, Italy
(4) Department of Medical and Biological Sciences, University of Udine, Udine, Italy
(5) Department of Medicine, Surgery and Health Sciences, University of Trieste, Trieste, Italy

Corresponding author:
Calamandrei, Gemma
Rome, Italy
gemma.calamandrei@iss.it

### Background

The Northern Adriatic Cohort II is a prospective mother-child cohort established in 2007 in coastal areas of Italy, Croatia and Slovenia to investigate the association between prenatal mercury exposure from maternal fish consumption and child neurodevelopment. The Italian Northern Adriatic Cohort II refers to the coastal area of the Friuli Venezia Giulia Region. At present, a biological bank collected during pregnancy and at birth from mother-child pairs is available. The data bank has collected neuropsychological outcomes measured at different developmental stages (18 months, 40 months), as well as a wide range of potential explanatory variables (socioeconomic indicators, diet habits, child postnatal exposures, maternal lifestyle). The Cross-Mediterranean Environment and Health Network Life (CROME LIFE+) project has followed 200 children born within The Northern Adriatic Cohort II for follow up at 7 years.

*Methods*

Chemical analyses consisted of measurements of the concentration of five neurotoxic metals (mercury, lead, manganese, cadmium, arsenic) in either hair or urine of each child. The neuropsychological tests included the Wechsler Intelligence Scale for Children-IV, the Developmental Neuropsychological Assessment-II, for the assessment of reading and writing skills, and the Child Behaviour Check List to identify behavioural and emotional problems in children. Ethical approval was granted by the Burlo Garofolo Child Hospital of Trieste.

*Results*

Results show that total mercury exposure during pregnancy and breastfeeding did not significantly affect neuropsychological performances at 7 years. The total mercury level in urine was strongly related to the fish consumption by the mother during pregnancy and breastfeeding; there was a positive effect of fish consumption by the child on several attentional and cognitive tasks at 7 years. We found an adverse effect of manganese as measured in children's hair on the five Wechsler Intelligence Scale for Children-II score, with statistically significant decrements of the general intelligence quotient and of verbal comprehension.

*Conclusions*

These results highlight the complex interaction between multiple chemical exposures, life style factors, and time of outcome assessment and support the usefulness of the integrated approach to risk modeling adopted by the CROME LIFE project.

*Funding Acknowledgments*

Supported by the CROME Grant N° LIFE12 ENV/GR/001040 "Cross-Mediterranean Environment and Health Network."

---

### Environmental Screening of Girls with Early Puberty Disorder

Ferran Campillo i López (1), Juan Antonio Ortega-García (2), Arancha Escribano-Muñoz (3), Esther Tobarra-Sánchez (2), Almudena Pernas Barahona (2), Alberto Cárceles-Álvarez (2), Miguel Felipe Sánchez-Sauco (2)

(1) Pediatric Environmental Health Specialty Unit, Garrotxa's Territorial Pediatric Team, Olot and Garrotxa's County Hospital, Olot, Spain
(2) Pediatric Environmental Health Specialty Unit, Universitary Hospital Virgen de la Arrixaca, Murcia, Spain
(3) Pediatric Endocrinology, Universitary Hospital Virgen de la Arrixaca, Murcia, Spain

Corresponding author:
Campillo i López, Ferran
Olot, Spain
campillo@pehsu.org

*Background*

A decrease of the age of menarche and thelarche has been observed during the last decades. The cause of most cases of precocious puberty in girls remains unknown. Some environmental factors such as obesity and adoption have been related to early puberty. Our objective is to explore environmental factors within a sample of girls with early puberty disorders.

*Methods*

We performed a cross-sectional study with a sample of 30 randomly selected girls who consulted at a tertiary hospital in an agricultural area of southeastern Spain with a diagnosis of precocious puberty, isolated premature thelarche, early puberty, early menarche, early adrenarche or early pubarche. Environmental factors previously described in literature studies were explored through an interview performed by a single interviewer. Auxiological parameters were measured at the moment of the interview. The body mass index and z-scores for each parameter were calculated. Written informed consent was obtained from all participants.

*Results*

6.9% of girls in the sample were adopted. The age of thelarche was positively and significantly correlated with family income (r 0.738, CI95% 0.342-1.345; p0.001), with the poorest girls being the ones that began puberty earlier. Of the total sample, 12% of the girls were overweight and 11.1% obese. Body mass index at the age of diagnosis was correlated, but not significantly, with the age of thelarche (r 0.702, CI95% -0.019 -1.423; p0.056). At least 80% of the girls were exposed to pesticides in different degrees. Another 80% of the sample was exposed to lice treatments, especially tea tree oil.

*Conclusions*

Adoption and obesity were present in the sample. The correlation between income status and menarche has been previously reported, but this is the first study to correlate poverty with earlier thelarche. Pesticide exposure is extremely common in our sample, probably due to the fact that agriculture is one of the most important activities in the area of study. Tea tree oil exposure has been related with gynecomastia in boys. The effect on puberty among girls remains unknown. An environmental approach of patients with early puberty disorders may help to create a hypothesis about the etiology and develop specific treatments.

### Quality of Life of Childhood Brain Tumor Survivors: Intervention, Analysis and Results

Eduardo Martínez-Salcedo (1,5), Alberto Cárceles-Álvarez (1,5), Juan Antonio Ortega-García (1,5), Vicente Vicente-Ortega (2), José Luis Fuster Soler (3), Helena Alarcón-Martínez (4), Salvador Ibáñez-Micó (4), Rosario Domingo-Jiménez (4)

(1) Pediatric Environmental Health Speciality Unit, University Hospital Virgen de la Arrixaca, Murcia, Spain
(2) Chair of Pathology, Faculty of Medicine, University of Murcia, Murcia, Spain
(3) Oncohematology Section, Pediatrics, University Hospital Virgen de la Arrixaca, Murcia, Spain
(4) Neurology Section, Pediatrics, University Hospital Virgen de la Arrixaca, Murcia, Spain
(5) Laboratory of Environment and Human Health, Instituto Murciano de Investigación Biosanitaria, Murcia, Spain

Corresponding author:
Cárceles-Álvarez, Alberto
Murcia, Spain
alberto@pehsu.org

*Background*
Our aim is to assess the socio-demographic and environmental factors, morbidity, and health related quality of life (HRQL) of childhood brain tumor survivors.

*Methods*
Through this cross-sectional study we examined pediatric survivors (diagnosed 0-14 years of age) out of treatment and more than 2 years since the diagnosis in a specialized unit. Evaluations were administered though questionnaires regarding socio-demographics, environment, clinical history, scale of sequelae effects (LESS), and PedsQL™ 4.0 parents and patients generic scale (2-18 years and young adult).

*Results*
Total population: 82. Died during the study: 3; Evaluated: 67. Male: 58.2%. Current mean age: 164.6 months (σ 76). Mean age at diagnosis: 62.1 months (σ 46). Mean follow-up: 108.1 months (σ 54.8). Low income: 28.8%. Low level of parental education: 30%. Tobacco smoke in the home: 61.2%. Physical exercise <1-2 d/week < 30 min: 53%. Epilepsy: 16.84%. Neurofibromatosis-1: 11.9%. International Classification of Childhood Cancer, Third edition: astrocytomas 47.7%, medulloblastomas/primitive neuroectodermal tumor 17.91%, craniopharyngiomas 7.4%, ependymomas 4.4%, others 5.9%. Location: supatenorial 28.35%, infratenorials 46.2%. Ventriculoperitoneal shunt: 19.4%. Surgery: 82.1%. Radiotherapy: 35.8%. Chemotherapy: 43.3%. Hematopoietic stem cell transplantation: 7.5%. Reintervention: 11.9%. LESS modified (>0p) 82.1%. Global HRQL average values: (2-18 years): reported by parents: 67.4 (normal values (NV) 81.3), reported by patient: 72.6 (NV 82.8); reported by young adult: 77.0 (NV 78.1)

### Conclusions

On average, this population of childhood brain tumor survivors shows socioeconomic difficulty, environmental risk, high prevalence of medical sequelae and a reduction of HRQL compared to the healthy normative population of children.

---

### Environment and Childhood Cancer Survivors: PLASECAP – MUR

**Alberto Cárceles-Álvarez (1,2), Juan Antonio Ortega-García (1,2), Miguel Felipe Sánchez-Sauco (1,2), Eduardo Martínez-Salcedo (1,2), Mayra Alejandra Orozco Llamas (3,4), Lizbeth Álvarez Gómez(3,4), Esther Tobarra Sánchez (1,2), Almudena Pernas Barahona (1,2), María Luisa Azurmendi Funes (1,2), José Luis Fuster Soler (5)**

**(1) Pediatric Environmental Health Speciality Unit, University Hospital Virgen de la Arrixaca, Murcia, Spain**
**(2) Laboratory of Environment and Human Health, Instituto Murciano de Investigación Biosanitaria, Murcia, Spain**
**(3) Mount Sinai School of Medicine, Pediatric Environmental Health Speciality Unit, New York, USA**
**(4) University Hospital Virgen de la Arrixaca, Murcia, Spain**
**(5) Oncohematology Section, Pediatrics, University Hospital Virgen de la Arrixaca, Murcia, Spain**

**Corresponding author:**
**Cárceles-Álvarez, Alberto**
**Murcia, Spain.**
**alberto@pehsu.org**

### Background

The National Cancer Institute considers that every child with cancer is a childhood cancer survivor (CCS) from the time of diagnosis, and will continue to be one for the rest of his or her life. In the last decades the pediatric cancer survival rate has increased progressively, reaching approximately 80%. The increase of survival has revealed the need for controlling late effects caused by treatments. International organizations recommend structured follow-up, and if possible, throughout their lifespan, for CCS. The objective of this work is to present the Long-Term Follow-Up Program for Childhood Cancer Survivors in the Region of Murcia (PLASESCAP-MUR).

### Methods

In the Region of Murcia (Spain) with an implemented public primary care health system, we have developed PLASESCAP-MUR for the follow-up of CCS, which follows a shared-care model for the transition from hospital to primary care that is tailored to the specific needs of each CCS.

### Results

The first objective of PLASESCAP is the education and promotion of healthier environments and lifestyles for the survivor, their family, and community, with the goal of improving their environmental standard and quality of life. The second objective is for early detection and screening for an appropriate management of the late effects arising from disease and treatments.

*Conclusions*

Long-term follow-up programs for CCS based on creating healthier environments, reducing and eliminating exposure to environmental carcinogens and promoting healthy lifestyles in order to reduce the incidence of late effects and to help CCS to achieve the highest quality of life possible are necessary.

### Health Related Quality of Life of Survivors of Childhood Extracranial Neoplasms

Mayra Alejandra Orozco Llamas (1,2,3), Alberto Cárceles-Álvarez (3,4), Miguel Felipe Sánchez-Sauco (3,4), Juan Antonio Ortega-García (3,4), Eduardo Martínez-Salcedo (3,4), María Luisa Azurmendi Funes (3,4), Esther Tobarra Sánchez (3,4), José Luis Fuster Soler (5)

(1) Mount Sinai School of Medicine, Pediatric Environmental Health Speciality Unit, New York, USA
(2) University Hospital Virgen de la Arrixaca, Murcia, Spain
(3) Laboratory of Environment and Human Health, Instituto Murciano de Investigación Biosanitaria, Murcia, Spain
(4) Pediatric Environmental Health Speciality Unit, University Hospital Virgen de la Arrixaca, Murcia, Spain
(5) Oncohematology Section, Pediatrics, University Hospital Virgen de la Arrixaca, Murcia, Spain

Corresponding author:
Cárceles-Álvarez, Alberto
Murcia, Spain
alberto@pehsu.org

*Background*

In the last decades, the pediatric cancer survival rate has increased spectacularly, reaching approximately 80%. This increase has revealed the need to control late effects arising from treatments and disease that affect the quality of life of childhood cancer survivors (CCS). The Long-Term Follow-Up Program for Childhood Cancer Survivors in the Region of Murcia (PLASESCAP-MUR) offers an integrated follow-up for CCS and among its objectives is environmental education and awareness and healthier lifestyles to improve environmental and life quality of survivors and their environment.

*Objectives*

Assess the health related quality of life of a group of survivors of extracranial neoplasms and evaluate the associations of demographic, clinical, and environmental factors with the results.

*Methods*

Descriptive cross-sectional study. The PedsQL™ 4.0 self-administered generic version for 4 to >18 year old survivors was completed by 85 survivors.

*Results*

Overall quality of life was 84.1 (81.7; 86.4). Subscales: physical health = 86.8 (84.1; 89.7), psychosocial health = 82.5 (79.9; 84.9), emotional domain = 75.4 (71.6; 78.9), social domain = 92 (89.3; 94.5), and educational domain = 79.9 (76.7; 83.2). The worst quality of life was reported by CCS that have been treated with radiotherapy (-9.0; p=0.007), that presented with a relapse of their disease (-9.2; p=0.033), or that presented with some adverse effect derived from the treatments used (-8.0; p<0.001). The environmental and lifestyle factors studied did not significantly influence the results.

*Conclusions*

The results obtained are similar to other international work. Strategies to improve quality of life of survivors are necessary, preferably focused on the creation of healthier environments.

### Lifestyles of Survivors of Childhood Hematologic Neoplasms

Mayra Alejandra Orozco Llamas (1,2,3), Alberto Cárceles-Álvarez (3,4), Miguel Felipe Sánchez-Sauco (3,4), Juan Antonio Ortega-García (3,4), Eduardo Ramos Elbal (3,4), Esther Tobarra Sánchez (3,4), Almudena Pernas Barahona (3,4), José Luis Fuster Soler (5)

(1) Mount Sinai School of Medicine, Pediatric Environmental Health Speciality Unit, New York, USA
(2) University Hospital Virgen de la Arrixaca, Murcia, Spain
(3) Laboratory of Environment and Human Health, Instituto Murciano de Investigación Biosanitaria, Murcia, Spain
(4) Pediatric Environmental Health Speciality Unit, University Hospital Virgen de la Arrixaca, Murcia, Spain
(5) Oncohematology Section, Pediatrics, University Hospital Virgen de la Arrixaca, Murcia, Spain

Corresponding author:
Cárceles-Álvarez, Alberto
Murcia, Spain
alberto@pehsu.org

*Background*

The prevention of late effects caused by treatments and the promotion of healthy environment and lifestyle are the two pillars of the Long-Term Follow-Up Program for Childhood Cancer Survivors in the Region of Murcia (Spain) (PLASESCAP-MUR) to achieve the highest levels of environmental and quality of life in childhood cancer survivors. The objective of this project is to analyze the lifestyles of survivors of childhood hematologic neoplasms.

*Methods*

Descriptive cross-sectional study. A total of 55 survivors were analyzed within the PLASESCAP from August 2015 to May 2016.

*Results*

There were 39 leukemias and 16 lymphomas. Mean age = 16.8 (15.6; 17.9). Of these, 9.4% are smokers and 50% occasionally consume alcohol. About 52% of the survivors engaged in physical activity less than three times a week. Almost 56% and 60% of the survivors eat less than one portion of fruit and vegetables per day, respectively. Finally, 53% use electronic devices more than 2 hours a day and 44% of them never engage in activities in contact with nature or do so only in summer.

*Conclusions*

More than half of the survivors do not meet the dietary and physical activity recommendations. Up to 50% of them consume alcohol with a mean age under 17. Programs that encourage the promotion of healthy lifestyles with a diet rich in fruits and vegetables, exercise, and regular contact with nature as well as avoidance of toxic habits and drugs such as alcohol or tobacco are necessary to obtain the best environmental standard and quality of life.

### Spatial Clustering of Childhood Leukemia in the Region of Murcia, Spain, 1998-2010

Alberto Cárceles-Álvarez (1), Mayra Alejandra Orozco Llamas (1,2), Blanca Espinosa López (1), Eduardo Ramos Elbal (1), Juan Antonio Ortega-García (1)

(1) Pediatric Environmental Health Speciality Unit. Clinical University Hospital 'Virgen de la Arrixaca', Murcia, Spain, Laboratory of Environment and Human Health, IMIB – LAIB, University of Murcia, Spain
(2) Mount Sinaí School of Medicine, New York, USA

Corresponding author:
Cárceles-Álvarez, Alberto
Murcia, Spain
alberto@pehsu.org

*Background*

The objective of this study is to test the causal hypothesis that exposure to carcinogens in utero leads to the onset of early childhood leukemia by exploring the presence of spatial and spatial-temporal clusters within administrative divisions in the region of Murcia, Spain, while considering the residential addresses of cases during pregnancy, early childhood and diagnosis.

*Methods*

The Pediatric Environmental Health Specialty Unit is carrying out an ongoing research project on the environment and pediatric cancer, collecting a pediatric environmental history (PEH) to create regional maps and analyze the geographical distribution of incident cancer cases in the region of Murcia. Between 1998 and 2010, 117 children were diagnosed with leukemia. Pregnancy, early childhood and diagnosis addresses were collected. Spatial and spatial-temporal clusters were then evaluated using the statistical software SatScan®.

*Results*

Leukemia was most common in children under 5 years of age. The incidence rate was 40.1 per 1 million children during the study period. Among subtypes of leukemia, ALL (acute lymphoblastic leukemia) has a greater incidence in children, occurring in 31.5 per million children, in comparison to AML (acute myeloblastic leukemia), affecting 8.6 in every million children. The incidence rate of all leukemias and subtypes increased in the time frame between 1998-2004 and 2005-2010. Spatial clusters were observed for leukemia in general and ALL, considering the pregnancy addresses and census district; however, no clusters were identified for the residential addresses during diagnosis.

*Conclusions*

The detection of spatial clusters in a localized area when examining the distribution of pregnancy addresses and census district areas lends proof to the hypothesis that fetal environmental exposure is likely implicated in the etiology of childhood leukemia.

---

### Secondhand Tobacco Smoke and Survival in Children With Acute Lymphoblastic Leukemias in the Region of Murcia (Spain)

Alberto Cárceles-Álvarez (3,4), Juan Antonio Ortega-García (3,4), Miguel Felipe Sánchez-Sauco (3,4), Eduardo Martínez-Salcedo (3,4), Mayra Alejandra Orozco Llamas (1,2,3), Esther Tobarra Sánchez (3,4), José Luis Fuster Soler (5)

(1) Mount Sinai School of Medicine, Pediatric Environmental Health Speciality Unit, New York, USA
(2) University Hospital Virgen de la Arrixaca, Murcia, Spain
(3) Laboratory of Environment and Human Health, Instituto Murciano de Investigación Biosanitaria, Murcia, Spain
(4) Pediatric Environmental Health Speciality Unit, University Hospital Virgen de la Arrixaca, Murcia, Spain
(5) Oncohematology Section, Pediatrics, University Hospital Virgen de la Arrixaca, Murcia, Spain

Corresponding author:
Cárceles-Álvarez, Alberto
Murcia, Spain
alberto@pehsu.org

*Background*

Environmental tobacco smoke is associated with diseases and premature death in childhood. It has been associated with an increased risk of several pediatric tumors, including acute leukemias, with variable results. There are certain clinical and biological features that have prognostic influences in childhood acute lymphoblastic leukemia (ALL). However, the observed plateau in survival rates in recent years suggests that there must be other unknown factors affecting prognosis. In adults, there are some reports linking active smoking with decreased survival in patients with myeloblastic leukemia, but this relationship has not been studied in the pediatric population. Our objective is to study the relationship between environmental tobacco smoke and overall survival in children diagnosed with ALL.

### Methods
Survival analysis in the MACAPEMUR (Environment and Pediatric Cancer in the Region of Murcia) cohort of patients between 0 and 15 years with ALL diagnosed from January 1998 to December 2013 in the Region of Murcia (Spain).

### Results
Sixty percent of children with ALL live with at least one parent smoking at diagnosis and in 26% of households both parents are smokers. Environmental tobacco smoke from maternal smokers after diagnosis was significantly associated with lower overall survival (HR = 9.92; 95% CI 1.89 – 51.89).

### Conclusions
Maternal smoking after diagnosis of ALL in children is an independent prognostic factor and easily adaptable in the overall survival of acute lymphoblastic leukemia. In order to increase survival and improve their environment and quality of life it is necessary to provide smoking cessation programs to parents of children with leukemia.

---

### Changing Kindergarten Practices with Localized Real-Time Information on Outdoor Air: the Experience of the CITI-SENSE Project in Oslo, Norway

Núria Castell, Sonja Grossberndt, Mirjam F. Fredriksen, Alena Bartonova

Norwegian Institute for Air Research, Kjeller, Norway

Corresponding author:
Castell, Núria
Kjeller, Norway
ncb@nilu.no

### Background
In Norway, children in kindergarten spend a lot of time outdoors in all weather, and there is a natural concern about the quality of outdoor air. It is well known that air pollution is associated with a wide variety of adverse health impacts in children, with greater impact on children with asthma. Kindergartens in Oslo that are situated close to streets with busy traffic, or in areas where wood burning is used for house heating, can experience many days with bad air quality, particularly during winter and spring. During these periods, updated information on air quality levels can help to plan outdoor activities and thus protect children's health.

### Methods
We tested new low-cost air quality monitoring platforms to provide air quality information to kindergartens. These platforms are smaller, cheaper and less complex to use than reference equipment. Performance evaluation shows that while they are less accurate and suffer from higher uncertainty than European Committee for Standardization reference equipment, they still can provide reliable coarse information to the local pollution. We received approval from the Norwegian ethical committee.

*Results*

We installed the monitoring platforms in 17 kindergartens in Oslo to measure nitrogen dioxide, nitrogen dioxide, ozone, carbon monoxide and particulate matter. The data is displayed using the Air Pollution Indication in a 5-colour scale, indicating if the air pollution is very low, low, rather low, rather high and very high (http://oslo.citi-sense.eu).

*Conclusions*

We arranged a focus group with the participation of the local administration, kindergarten staff and parents to understand their opinion and needs related to the air quality information that was generated in Oslo kindergartens. They expressed concern about the data quality but agree that having updated information on the air quality in the surrounding kindergartens can help to reduce children's exposure to air pollution or to better plan medication to reduce asthma symptoms.

---

### Engaging Schoolchildren in Understanding the Indoor Environment Using Citizen Science

Johanna A. Robinson (1,2), Joanne Crawford (3), Yaela N. Golumbic (4,5), Britt Ann K. Høiskar (6,7), Sverre B. Holøs (8), Milena Jovasevic-Stojanovic (9), Dusan Topalovic (9), Milos Davidovic (9), David Kocman (1), Núria Castell (6), Alena Bartonova (6)

(1) Department of Environmental Sciences, Jožef Stefan Institute, Ljubljana, Slovenia
(2) Jožef Stefan International Postgraduate School, Ljubljana, Slovenia
(3) Research Division, Institute of Occupational Medicine, Edinburgh, United Kingdom
(4) Faculty of Education in Technology and Science, Technion, Haifa, Israel
(5) Faculty of Civil and Environmental Engineering, Technion, Haifa, Israel
(6) Norwegian Institute for Air Research, Kjeller, Norway
(7) The Norwegian Asthma and Allergy Association, Oslo, Norway
(8) Building and Infrastructure Division, SINTEF, Oslo, Norway
(9) Department of Radiation & Environmental Protection, Vinča Nuclear Institute, University of Belgrade, Belgrade, Serbia

Corresponding author:
Castell, Núria
Kjeller, Norway
ncb@nilu.no

*Background*

Children spend a substantial part of their childhood in school buildings. The school staff might not have the time or knowledge to investigate the indoor environment, even though they acknowledge that it could be improved, nor do they have the capacity to have a dialogue with the students regarding a good indoor environment.

*Methods*
As part of a CITI-SENSE project, we used citizen science approaches to investigate how the situation can be improved. We engaged schools to study the indoor environment with the help of sensor technology and support from scientists. Some schools took this as an opportunity to conduct individual student-led research as part of the school curriculum, while others wanted to confirm the indoor environmental issues the school was known to have.

*Results*
We provided 13 schools in Belgrade, Edinburgh, Ljubljana, Oslo and Haifa with low-cost sensor units, which collect indoor environmental data in near real time, and gave them the resulting data as graphs or data tables. The students defined and conducted related research, e.g., studied how the carbon dioxide concentration changes depending on the amount of students in the classroom, or investigated the variability of indoor parameters across classrooms and workshops in the schools. Others investigated why particulate matter is high when students are present in the classroom. A school with a known Radon issue was happy to investigate further which rooms were the most problematic. Questionnaires conducted by the scientists assessed learning outcomes related to general knowledge on the indoor environment or ability to think critically about the environment.

*Conclusions*
These activities created awareness in the schools, increased the individual students' and teachers' knowledge about indoor air, and sometimes led to mitigation measures at schools such as changing cleaning practices.

---

### Indoor Air and Improving Children's Health, the School Indoor Pollution and Health-Observatory Network in Europe and the School Environment and Respiratory Health of Children Projects

Eva Csobod

Regional Environmental Center for Central and Eastern Europe, Szentendre, Hungary

Corresponding author
Csobod, Eva
Szentendre, Hungary
ecsobod@rec.org

*Background*
The first of two projects presented here is the School Indoor Pollution and Health-Observatory Network in Europe project, (SINPHONIE), 2010-2014, which established a scientific/technical network to act at the EU level with the long-term perspective of improving air quality in schools and kindergartens and reducing the risk and burden of respiratory diseases among children and teachers due to outdoor and indoor air pollution. The project also supports future policy actions by formulating guidelines, recommendations and risk management options for better air quality and associated health effects in schools.

The second is the pan-European School Environment and Respiratory Health of Children (SEARCH II) project (2010-2013). It is the second phase of the SEARCH initiative and was developed in order to expand monitoring of children's health and air quality, and to assess energy use in selected schools in 10 countries. Four new countries (Belarus, Kazakhstan, Tajikistan and Ukraine) joined the six countries that participated in The School Environment and Respiratory Health of Children I (Albania, Bosnia and Herzegovina, Hungary, Italy, Serbia and Slovakia).

*Methods*

The School Indoor Pollution and Health-Observatory Network in Europe has produced guidelines for healthy school environments (www.sinphonie.eu). The guidelines are intended to be generally applicable in most school environments in Europe. However, as each school environment is unique (in terms of design, climatic conditions, operational modes, etc.), the guidance needs to be adapted at the national or local level. This network was initiated and funded by the European Parliament. The project coordination committee includes Éva Csobod, REC, Eduardo de Oliveira Fernandes, IDMEC-FEUP, Peter Rudnai, NIEH and Stylianos Kephalopoulos, JRC.

*Results*

The final reports of the SINPHONIE project summarise the work performed by a consortium of 38 partners from 25 countries that involved around 300 people of specialised scientific and/or technical backgrounds (www.sinphonie.eu).

The extensive database of the SEARCH II project contains information on 7,860 children from 388 classrooms in 100 schools in 10 countries. It has created a unique opportunity to study a wide variety of school indoor and outdoor environments, to measure outdoor and indoor concentrations of several air pollutants, and to study the associations between the school environment and children's health (www.search.rec.org).

*Conclusions*

The implementation of both projects was coordinated by the Regional Environmental Center for Central and Eastern Europe (REC). The funders, EP/DG SANCO and the Italian Ministry of Land and Sea (IMELS), provided targeted contributions to research on air quality and children's health to improve the quality of life for children in the future.

### Lifelong Exposure to Green Space and Attentional Development: A Prospective Birth Cohort Study

Payam Dadvand (1,2,3), Christina Tischer (1,2,3), Marisa Estarlich (2,4), Sabrina Llop (2,4), Albert Dalmau (1,2,3), Mónica López-Vicente (1,2,3), Antònia Valentín (1,2,3) Mireia Gascon (1,2,3), Mònica Guxens (1,2,3,5), Xavier Basagaña (1,2,3), Mark J Nieuwenhuijsen (1,2,3), Ferran Ballester (2,4), Jordi Sunyer (1,2,3)

(1) Barcelona Institute for Global Health, Centre for Research in Environmental Epidemiology, Barcelona, Spain
(2) Spanish Consortium for Research and Public Health, Instituto de Salud Carlos III, Madrid, Spain
(3) Pompeu Fabra University, Barcelona, Spain
(4) Fundación para el Fomento de la Investigación Sanitaria y Biomédica de la Comunitat Valenciana, Universitat de València, Universitat Jaume I, Joint Research Unit of Epidemiology and Environmental Health, Valencia, Spain
(5) Department of Child and Adolescent Psychiatry/Psychology, Erasmus University Medical Centre, Sophia Children's Hospital, Rotterdam, the Netherlands

Corresponding author:
Dadvand, Payam
Barcelona, Spain
pdadvand@isglobal.org

*Background*
Natural environments including green spaces are thought to exert beneficial impacts on brain development; however, the available evidence on such impacts is still very scarce. The aim of this study was to evaluate the association between lifelong exposure to residential surrounding greenness and cognitive development during preschool and early primary school years.

*Methods*
A longitudinal study based on data from a well-established multi-centre population-based birth cohort in Sabadell and Valencia (Spain) followed participants from pregnancy (2003-2006) till 7 years of age (2012-2013). A satellite-derived normalized difference vegetation index was used to quantify surrounding greenness (100 m, 300 m, and 500 m buffers) of participants' residential addresses at birth and at 4 and 7 years of age. Attentional function was assessed using Kiddie-Continuous Performance Test at age 4 (N=888) and Attention Network Task at age 7 (N=978). Mixed effects linear and negative binomial models with cohort random effect were developed to quantify the association between greenness exposure and attention, adjusted for relevant covariates. Ethical approval was granted by the ethical committee of each cohort.

*Results*
Higher average residential surrounding greenness between birth and age 4 was associated with lower omission errors and lower hit reaction time standard error, both indicators of inattentiveness in the Kiddie-Continuous Performance Test. Similarly, higher average residential surrounding greenness between birth and age 7 was associated with lower hit reaction time standard error in the Attention Network Task.

*Conclusions*

This is the first longitudinal study to report on the impact of lifelong green space exposure on cognitive development in general and attentional development in particular. It found this exposure was associated with enhanced attentiveness in preschool and primary school children. The associations for attentiveness were consistent using different tests (Kiddie-Continuous Performance Test and Attention Network Task) and different exposure windows (birth to 4 years and birth to 7 years).

### The PERSIAN Birth Cohort: A Work in Progress

Payam Dadvanda (2), Reza Malekzadeh (3), Hossein Poustchi (3), Amir Hooshang Mehrparvar (4), Mohammad Javad Zare (4), Akhgar Ghassabian (5), Amirhossein Modabbernia (6), Mark J. Nieuwenhuijsena (2), Jordi Sunyera (2), Roya Kelishadi (7)

(1) Barcelona Institute for Global Health, Centre for Research in Environmental Epidemiology, Barcelona, Spain
(2) Spanish Consortium for Research and Public Health, Instituto de Salud Carlos III, Madrid, Spain
(3) Digestive Diseases Research Institute, Tehran University of Medical Sciences, Tehran, Iran
(4) Shahid Sadoughi University of Medical Sciences, Yazd, Iran
(5) Department of Child and Adolescent Psychiatry/Psychology, Erasmus MC-Sophia, Rotterdam, the Netherlands
(6) Department of Psychiatry (A.M.), Icahn School of Medicine, Mount Sinai Hospital, New York, USA
(7) Research Institute for Primordial Prevention of Non Communicable Disease, Isfahan University of Medical Sciences, Isfahan, Iran

Corresponding author:
Dadvand, Payam
Barcelona, Spain
pdadvand@@isglobal.org

*Background*

The influence of various exposures during the pre- and postnatal period is not limited to reproductive and childhood outcomes and can extend over a lifetime, a corpus of knowledge embodied by the Developmental Origins of Health and Diseases (DOHaD) concept. The overarching aim of this project is to study DOHaD in the Iranian population. Towards this aim, we will evaluate the impact of socioenvironmental, psychological and genetic factors on pregnancy outcomes, child mental and physical health, growth, and development, and early- and late-onset chronic non-communicable diseases.

*Methods*

The Prospective Epidemiological Research Studies of the Iranian Adults, Adolescents and Newborns birth cohort is designed as a multi-centre network of population-based birth cohorts in areas representing cultural and contextual variations in Iran. Standardised protocols for data collection, maintenance, and analysis tailored for the Iranian population with the prospect of future international collaborations have been developed. The cohort will be conducted in 5 phases: periconception to birth, infancy, childhood, adolescence, and adulthood. Data collection will be based on computerized questionnaires, biological samples, physical examinations, clinical tests, hospital records, and surveys of living environment.

### Results

The pilot phase will start in Shahreza (Isfahan) and Yazd in April 2016 and is expected to last 2 calendar years. In each study site, 2000 pregnant women will be recruited during their first pregnancy visit (at the end of the 1st trimester). We will utilize a combination of public and private healthcare providers in order to ensure that the participants represent the ethnic and socioeconomic distribution of the underlying population.

### Conclusions

The Prospective Epidemiological Research Studies of the Iranian Adults, Adolescents and Newborns birth cohort will advance our knowledge of DOHaD in the Iranian population. Moreover, it will establish a platform in the Middle East for participating in international studies of DOHaD.

---

### Spatial Pattern Analysis of Hepatitis A Cases in Children using Geostatistical Analysis of Geographical Information Systems in Turkey

Ahmet Ozgur Dogru (1), Ruusa Magano David (2), Necla Ulugtekin (1), Cigdem Goksel (1), Dursun Zafer Seker (1), Seval Sozen (3)

(1) Department of Geomatic Engineering, Istanbul Technical University, Istanbul, Turkey
(2) Institute of Informatics, Geoinformation Technology Programme, Istanbul Technical University, Istanbul, Turkey
(3) Department of Environmental Engineering, Istanbul Technical University, Istanbul, Turkey

Corresponding Author:
Dogru, Ahmet Ozgur
Istanbul, Turkey
ozgur.dogru@itu.edu.tr

### Background

Epidemiology is the scientific study of the spread and control of diseases as a function of time and location. Epidemiology follows the patterns of a disease on people in their healthy and unhealthy periods; and tracks their history of illness, especially to understand its nature and characteristics. In this context, spatial data is strongly required in order to understand the spatial pattern (distribution and expansion) of the illness. Epidemiologists have traditionally used maps to analyze the spatial characteristics of illnesses in terms of relations between location, surrounding environment, and cases. Developing information technologies introduced geographical information systems (GIS) as a strong tool to be used in spatial decision making applications such as surveillance and monitoring of diseases. Geographical information systems and geostatistical techniques are used to account for spatially varying population sizes and spatial patterns in the surveillance and monitoring of diseases. In environmental and public health, identification and quantification of the disease patterns provide the first steps towards increased understanding and possibly, control of a particular disease. Spatial statistical methods, of which geostatistics is a subset, offer a means for us to use such location information to detect and quantify patterns in public health data and to investigate the degree of association between potential risk factors and disease.

### *Methods*

This study presents a general overview of the geostatistical methods used in epidemiology, experimenting an innovative geographical information systems-based approach to the analysis of epidemiological data. The paper additionally aims to analyze the spatial pattern of Hepatitis A cases in children at ages under 15. In this context, province-based official data including the number of Hepatitis A cases and deaths by gender and age were provided monthly from 2001 to 2011. The data were classified by age considering 3 main groups: 0-4, 5-9, and 10-14 years of age. Incidence rates and morbidity of Hepatitis A cases were considered as the main parameters for primary analysis of the cases by province. Time series maps were prepared and Natural Neighbor analysis was also applied to introduce the spatial pattern of case distribution in time. Additionally, Moran's I and Local Indicators of Spatial Association analysis were respectively applied to introduce the characteristics of the global and local spatial pattern of cases in time. Ethical approval was granted by the Human Research Ethics Committee of the Istanbul Technical University.

### *Results*

Time series maps of Hepatitis A cases in children concluded that incidence rates of the cases children under the age of 5 were significantly lower than the other age groups, while incidence rates the highest for children in the 5-9 age group during the study period. Although the results outlined a timely decrease in the incidences of Hepatitis A in Turkey, high incidence rates occurred in 2001, 2005 and 2007 and were significant for children, especially between the ages of 5-9. Spatial pattern analysis of the cases showed that incidence rates were relatively higher in the Eastern Mediterranean and South-eastern Anatolian Regions covering Ceyhan, Asi and Southeast part of the Fırat-Dicle river basins in Turkey. Gaziantep, Kilis and Hatay provinces were the hotspots of these regions. Incidence rates of cases in children aged 5-9 were also higher in the Aegean and Central Anatolia regions, especially in Gediz and Kızılırmak river basins.

### *Conclusions*

At the conclusion of this study, the spatial distribution of Hepatitis A cases in children from 2001 to 2011 in Turkey were mapped and analyzed using GIS technology. Since Hepatitis A is a waterborne communicable disease, spatial distribution was also examined considering the coverages of the river basins in Turkey. The study outlined the regions and specific provinces where intensive water treatment facilities were needed in Turkey during the study period.

### Persistent Organic Pollutants in Women's Breast Milk and Proximity to Municipal Waste Incinerators in the UK

Philippa Douglas (1), Danielle Ashworth (1), Evie Kritioti (1), Emily Amezdroz (1), Vincenzo Salerno (1), Anna Hansell (1,2), Paul Elliott (1), Mireille B. Toledano (1)

(1) MRC-PHE Centre for Environment and Health, Department of Epidemiology and Biostatistics, School of Public Health, Imperial College London, London, United Kingdom
(2) Imperial College Healthcare NHS Trust, Public Health and Primary Care, London, United Kingdom

Corresponding author:
Toledano, Mireille
London, UK
m.toledano@imperial.ac.uk

*Background*
EU legislation restricting landfilled waste has led to an increase in Municipal Waste Incineration (MWI) within the UK. Persistent Organic Pollutants (POPs), including dioxins and heavy-metals, released from MWIs have been found in elevated levels in areas surrounding these sites. Due to public concern over health risks associated with MWIs, this project aims to quantify POPs in human breast-milk to examine relationships between individual level POP uptake in proximity to MWIs.

*Methods*
Recruitment was completed in three phases via postal invites. Primiparous women between 18-40 weeks gestation, aged >18 years and living within 20 km of three MWIs were invited. Women were asked to provide up to 120 ml of breast-milk and complete a questionnaire (including questions on lifestyle, diet, residential history). Selected breast-milk samples have been analyzed for selected POPs, depending on the amount of milk provided

*Results*
587 women were recruited (14% recruitment rate), 329 provided milk (56% completion rate). Modelled $PM_{10}$ emissions from MWIs have been used as a proxy for incinerator exposure gradients for other pollutants, such as POPs, which cannot be directly modelled. Ground-level modelled estimations of $PM_{10}$ concentrations from incinerators is very low (yearly averages of incinerator postcode level concentrations varied from 1.00 10-8 to 5.53 10-5 mg m-3, 3-5 orders of magnitude lower than ambient background levels. 224 (38%) women live in areas in the top tertile of modelled incinerator $PM_{10}$ exposures and 203 (35%) women in the bottom tertile. Initial chemical analysis results will inform whether there are significant differences in POP or dioxin concentrations in women by MWI, proximity to MWI, and if any relationships are modified by lifestyle factors.

*Conclusions*
This study is the first and largest in the UK to provide individual level POP exposure estimates in proximity to MWIs and improve understanding of the diverse routes of dioxin exposure. It will also aid the interpretation of findings from an ongoing national study of MWIs and risk of adverse birth outcomes.

### Lead in Soil: Reducing Exposure to Young Children

Martin Eggens (1), Piet Otte (2)

(1) Municipal Health Service Groningen, Groningen, The Netherlands
(2) National Institute for Public Health and the Environment, Bilthoven, the Netherlands

Corresponding author:
Eggens, Martin
Groningen, the Netherlands
martin.eggens@ggd.groningen.nl

### *Background*
In young children, exposure to lead is associated with a reduced IQ (intelligence quotient) score even when exposure rates are low. On a population level, the loss of a few IQ points can have large consequences. For instance, the loss of one IQ point on a population level means an estimated 2% loss of productivity. It can also lead to an increase of people with (very) low IQ who need increased care and support. The European Food Safety Authority (EFSA) concluded that there is no evidence for a threshold for critical lead-induced effects. The main uptake pathways in young children are hand-mouth contact while playing in contaminated soil and the consumption of home-grown vegetables.

### *Methods*
Using the integrated exposure uptake biokinetic (IEUBK) (a physiologically-based pharmacokinetic (PBPK)) model from the Environmental Protection Agency (EPA) and CSOIL from the National Institute for Public Health and Environment (RIVM), the maximum acceptable lead content in soil was calculated. For the calculations, the doses related to a loss of one and three IQ points were used. Calculations were made for different uses of the soil (i.e. vegetable garden, living with (vegetable) garden and places where children play).

### *Results*
The calculated maximum lead content in soil for all soil uses was lower than the prevailing legislation. Recommendations are needed to lower the exposure of young children to a reasonably achievable level.

### *Conclusions*
The Dutch public health services have set up recommendations for municipalities for managing health risks due to the exposure of soil lead. These recommendations include communication and education to residents about simple measures to reduce the exposure of soil lead and the removal of hot spots. Examples of recommendations to residents include having children play in specially prepared clean sand areas and growing vegetables in containers with clean soil separated from contaminated soil. In addition, it is advised to wash the hands of children frequently and to clean the in-home area by frequent vacuuming.

### Cotinine, Cadmium and Polycyclic Aromatic Hydrocarbon Levels in Children's Urine as a Result of Environmental Tobacco Smoke Exposure in Two Spanish Locations

Marta Esteban, Juan José Ramos, Helena García, Myriam Catalá, Argelia Castaño

Environmental Toxicology, National Environmental Health Center, Instituto de Salud Carlos III, Madrid, Spain

Corresponding author:
Esteban, Marta
Madrid, Spain
m.esteban@isciii.es

*Background*

Children are especially vulnerable to secondhand smoke (SHS) due to their reduced ability to metabolize and excrete toxic chemicals. Children exposed to SHS are at increased risk of developing asthma, sudden infant death syndrome, and ear and respiratory infections. In Spain, after 2011, a stricter anti-smoking legislation led to a reduction in the exposure in public places, however this reduction was not observed in private environments, and children are mostly exposed to SHS at home.

*Methods*

In the framework of the Demonstration of a Study to Coordinate and Perform Human Biomonitoring on a European Scale project (DEMOCOPHES), urinary levels of cotinine, cadmium and polycyclic aromatic hydrocarbon (PAH) metabolites (1-hydroxypyrene and hydroxyphenanthrene isomers) were studied in relation to SHS exposure. First morning urine samples corresponding to 120 children aged 6-11 years in urban (Madrid city) and rural (Añover de Tajo, Toledo) areas were collected from October 2011 to January 2012. Sociodemographic and environmental tobacco exposure data were collected by an epidemiological questionnaire. Cotinine levels were analyzed by high resolution liquid chromatography coupled to mass spectrometry in tandem mode and cadmium was determined by dynamic reaction cell inductively coupled plasma mass spectrometry. For PAH metabolites, high performance liquid chromatography-fluorimetric detection analyses were performed. Ethical approval was granted by the Ethical Committee of the Instituto de Salud Carlos III.

*Results*

The geometric mean for cotinine was 685.8 μg/g creatinine for smoker mothers and 0.6 μg/g creatinine for non-smoker mothers (max 3779.0 and 4.1 μg/g creatinine, respectively). In children, the geometric mean for cotinine was 4.62 μg/g (smoker mother) and 0.86 μg/g (non-smoker mother) with maximum values of 86.28 and 10.91 μg/g creatinine, respectively. For all children, the geometric mean (confidence interval 95%) for cadmium was 0.05 μg/g creatinine (0.04-0.05), for 1-hydroxypyrene 0.15 μg/g creatinine (0.10-0.24) and 0.22 μg/g creatinine (0.13-0.37) for hydroxyphenanthrene isomers. Significant differences were observed regarding residence area, age, number of smokers per household and their smoking habits.

*Conclusion*

The home environment is a significant predictor of the cotinine levels in children and therefore efforts are required to protect children from SHS exposure in scenarios out of the scope of application of the current anti-smoking legislation.

*Acknowledgments*

Funded by MAGRAMA - ISCIII agreement, SEG 1251/07, SEG 1112/10, SEG1321/15 & DEMOCOPHES (LIFE09/ENV/BE/000410).

### Preventing Exposure to Environmental Hazards — Role of Primordial Prevention

**Ruth Etzel**

**Office of Children's Health Protection, US Environmental Protection Agency, Washington, DC, USA**

**Corresponding Author:**
**Etzel, Ruth**
**Washington DC, USA**
**Etzel.Ruth@epa.gov**

Protecting children from environmental hazards requires individual clinicians to be alert to environmental causes of disease, but it also is important to adopt broad-based societal interventions; this is considered primordial prevention. Primordial prevention aspires to establish and maintain conditions to minimize hazards to health. It consists of actions and measures that inhibit the emergence and establishment of environmental, economic, social and behavioral conditions.

In the field of environmental health, population-level actions are needed to address, for example, the problem of global climate change. Clinicians can raise awareness and be effective advocates for action on the national and international level to promote energy efficiency and renewable energy production while decreasing incentives for continued production and consumption of carbon-intensive fuels. The American Academy of Pediatrics issued a policy statement and technical report on the health effects of climate change on children that encourage child health care professionals to engage in efforts to reduce national $C0_2$ emissions, and major medical journals and organizations have highlighted the importance of health professional involvement. In 2015, Pope Francis spoke out on the threats of global climate change to future generations in his encyclical Laudato Si, On the Urgent Need for Care of Our Common Home. When clinicians address these concerns and work to reduce the country's use of fossil fuels that result in $C0_2$ pollution, they are practicing primordial prevention.

The influential role child health professionals are playing in national and international efforts to reduce environmental threats to children is not new. In 1957, the American Academy of Pediatrics

(AAP) launched this field when it formed a committee to educate providers about the importance of recognizing, treating and preventing exposure to radiation, the first environmental hazard to be noted by the AAP. In 1998, Pediatric Environmental Health Specialty Units were established by the Agency for Toxic Substances and Disease Registry and the US Environmental Protection Agency to provide resources about children's environmental health to clinicians and communities. In 1999, the American Academy of Pediatrics published the first Handbook of Pediatric Environmental Health; a 3rd edition was published in 2012. A Textbook of Children's Environmental Health was published in 2014. Many international bodies have endorsed the need for healthier environments for children and called for actions to reduce children's exposures to environmental hazards.

Primordial prevention is an integral part of the practice of pediatrics. It is foundational to addressing the major environmental health problems facing children today.

---

### Radiation Imaging Communication (Cooperation) in Pediatric Healthcare: Awareness, Accountability, and Action

Donald Frush (1,2)

(1) Medical Physics Graduate Program, Professor of Radiology, Duke University, North Carolina, USA
(2) Alliance for Radiation Safety in Pediatric Imaging/Image Gently Campaign, USA

Corresponding author:
Frush, Donald
Durham, North Carolina, USA
donald.frush@duke.edu

### *Background*

Professional responsibilities for safe and effective diagnostic imaging consist of three A's: radiation awareness, collective accountability for protection of our patients (both adult and pediatric), and actions to achieve this end. These have been fundamental responsibilities in diagnostic imaging since the discovery of x-rays over 120 years ago. Concurrently, there have been remarkable advancements in imaging procedures that use ionizing radiation, including x-rays (radiography), fluoroscopy, and computed tomography (CT).  This imaging portfolio clearly improves management and saves lives minute by minute, day by day across the world. Rapidly evolving technology affords both opportunities for improved service, but also challenges with the fundamental responsibilities for radiation safety. The principle components of radiation protection in medicine consist of justification (the imaging procedure is indicated) and optimization (the procedure is done in an appropriate fashion to balance diagnostic quality with risk considerations, including radiation exposure).

### *Methods/Results*

Both justification and optimization are embodied in the action aspect of our professional responsibilities. Justification is really a shared duty between all stakeholders, including healthcare providers who request imaging procedures and members of the imaging team. While the branding using "justification" seems relatively simple, it is actually quite complicated. There are a myriad of factors which come into play in determining which of any imaging modality is appropriate. Many of these are global, and some are regional, such as in the US with defensive medicine. The fundamental success for justification requires communication between healthcare providers (e.g. decision support) as well as the children we care for and their care providers.

### *Conclusions*

Development on both fronts requires consensus efforts (cooperation); a partnership of shaking hands rather than pointing fingers and assigning blame. Success may be more effectively and efficiently realized through a fourth A: assurance. This is exemplified through the Image Gently Alliance and the Alliance's mission for radiation education awareness as an advocate in the care of children. Such efforts can serve as global models for improved imaging care of children.

---

### Radiation Risk and Strategies for Informed Use of Medical Imaging in Children: What Care Providers Should Know

Donald Frush (1,2)

(1) Medical Physics Graduate Program, Professor of Radiology, Duke University, North Carolina, USA
(2) Alliance for Radiation Safety in Pediatric Imaging/Image Gently Campaign, USA

Corresponding author:
Frush, Donald
Durham, North Carolina, USA
donald.frush@duke.edu

### *Background*

Diagnostic imaging is lauded as one of the most important advancements in medical care for both adults and children. The benefits are accompanied by risks, such as the potential cancer risk from the incumbent exposure to ionizing radiation. Radiation risk in diagnostic imaging can be approached through a review of patterns of use, radiation dose measurements and doses delivered, potential risk, and strategies to mitigate risk.

### *Methods/Results*

The use of medical imaging, especially CT, has increased rapidly and globally over the past three decades for a variety of reasons. In the US, CT now accounts for 25% of the per capita exposure annually, included background (natural) sources. Exposures in medical imaging are considered low level radiation although CT is a relatively greater radiation exposure than x-rays, or fluoroscopy. Approximately 5-10% of CT examinations are performed in children. Based on data from several sources, especially atomic bomb survivors, a risk, at most small, has been attributed to medical exposures in the range of some of the higher dose procedures such as CT for children (in general a more vulnerable population).

The principle components of radiation protection in medicine consist of justification (the imaging procedure is indicated) and optimization. That is, the procedure is done in an appropriate fashion to balance diagnostic quality with risk considerations including radiation exposure.

General considerations for appropriate performance consist of minimizing the exposure time or views (e.g. fluoroscopy); examining only the necessary area; and adjusting the radiation exposure to the region scanned, clinical question(s), and the size of the child. Specific measures for CT include adjustment of scan settings, restricting multiple series taken of the same area, and avoiding overlapping of scan regions. There are special considerations for children in performing CT examinations such as the use of IV contrast media that must be not be neglected. There are also new technologies to help optimize the balance between dose and quality.  Recently, dose monitoring programs for all patients have become a responsibility for the medical community.  Finally, informed strategies supplementing requisite regulatory, guidance and accreditation components must include education.

*Conclusions*

Understanding and implementing appropriate strategies when imaging children will help in optimizing the value in medical care.

---

### Communicating Radiation Risks in Pediatric Imaging

Maria Perez (1), Donald Frush (2,3), Marie-Noël Bruné Drisse (1)

(1) Department of Public Health, Environment and Social Determinants of Health, World Health Organization, Geneva, Switzerland
(2) Medical Physics Graduate Program, Professor of Radiology, Duke University, North Carolina, USA
(3) Alliance for Radiation Safety in Pediatric Imaging/Image Gently campaign, USA

Corresponding author:
Frush, Donald
Durham, North Carolina, USA
donald.frush@duke.edu

*Background*

The use of ionizing radiation diagnostic imaging procedures for children has rapidly increased globally during the past 20 years, including computed tomography as a valuable tool for assessing pediatric illness and injury, as well as pediatric image-guided procedures, which may replace more complex surgical options. While the clinical value of imaging involving the use of radiation for pediatric health-care is unquestionable, this is often juxtaposed with children's relative increased susceptibility to radiation, including the potential for cancer from low-level exposures. While individual radiation risks are at most quite small, radiation protection in pediatric imaging is a public health issue due to the large population exposed and public concern. This demands policies and actions that recognize, maximize, and promote the multiple health benefits that can be obtained, which at the same time minimize potential health risks of the radiation exposure with the ultimate purpose of ensuring that the benefit outweighs potential harm. Radiation risk communication has a key role to inform the appropriate risk-benefit dialogue in healthcare settings. Healthcare providers requesting and/or performing radiological imaging procedures in children have a shared responsibility to communicate radiation risks to patients, parents and other caregivers.

*Methods/Results*

WHO organizes this seminar to present the new document "Communicating radiation risks in pediatric imaging: information to support health care discussions about benefit and risk" available at http://www.who.int/ionizing_radiation/pub_meet/radiation-risks-pediatric-imaging/en/ . This document is intended to serve as a tool for healthcare providers to communicate risks associated with pediatric imaging procedures. The session will discuss the effective and balanced benefit-risk dialogue in pediatric imaging and how this can be achieved to improve health service.

*Conclusions*

This WHO communication tool will serve as a basis to further develop training packages to improve communication skills of health care providers, as well as advocacy and information materials targeting patients, parents, other caregivers and the public.

---

### Birth and Growth Without OH: Detection and Follow-Up From Pregnancy Until the End of Adolescence of Children at Risk of Neurobehavioral Disorders Through Exposure of Alcohol and Illegal Drugs

María Luisa Azurmendi Funes (1,4), Esther Tobarra Sánchez (1,4), Miguel Felipe Sánchez-Sauco (1,4), Alberto Cárceles-Álvarez (1,4), Ferrán Campillo I López (1,4), Carolina Diana Jaimes Vega (1,4), Almudena Pernas Barahona (1,4), Isabel Saura Martínez (2), Sonia Hernández López (1,4), Mayra Alejandra Orozco Llamas (3), Juan Antonio Ortega-García (1,4)

(1) Pediatric Environmental Health Speciality Unit, Psychology of Children and Adolescents, Pediatrics Service, Universitary Hospital Virgen de la Arrixaca, Murcia, Spain
(2) Clinical Universitary Hospital Santa Lucía, Cartagena, Murcia, Spain
(3) Mount Sinai School Of Medicine, New York, USA
(4) Laboratory Environment and Human Health, Instituto Murciano de Investigación Biosanitaria, Murcia, Spain

Corresponding author:
Funes, María Luisa Azurmendi
Murcia, Spain
azurmendi_87@hotmail.com

*Introduction*

This integrated study examines the prevention and detection/follow-up of children at risk of neurobehavioral disorders associated with prenatal and/or postnatal exposure to alcohol and illegal drugs. We utilized the Elijo Mas Sano (Choose Healthier) registry of children at environmental risk from drugs and alcohol.

### Methods

For this descriptive study of environmental clinical history we observed the first 39 patients of Elijo Mas Sano. We included clinical, social and neurodevelopmental evaluations. The Elijo Mas Sano criteria of inclusion was a) >20 g/day of alcohol and/or 3 incidents of binge drinking of ≥40 g/day during periconceptional, gestational, or lactation stages and/or b) fetal exposure to any illegal drug at least once a week. Ethical approval was granted by the Clinical Research Ethics Committee of the University Hospital Virgen de la Arrixaca.

### Results

The study group was comprised 39 subjects, 19 girls and 20 boys. The gestation period was an average of 38.26 weeks (min. 30; max. 41). There were 5 premature newborns (<36 gestation week). The average birth weight (≥36 gestation week) was 3,055 g (min. 2,433.9; max. 3,676.1). The average consumption of alcohol at early pregnancy in mothers was 33.55 g (confidence interval 95% 23.7-43.2).

In the study group, 33% reported 3 or more incidents of binge drinking. Fetal exposure to illegal drugs occured in 18% of the study group. Four patients had neonatal anthropometry (≥36 gestation week) with an average head circumference of <p10, a birth weight of <p10: 9, and 3 patients had a height of <p10. There were 6 patients with major congenital malformations. Two patients had chromosomal comorbidities.

We evaluated 37 of the 39 patients' facial features: palpebral fissure (<-2 standar deviation): 2, philtrum (4-5): 2, upper lip (4-5): 4. The Battelle test neurodevelopmental evaluation was given to 30 of the 39 subjects to examine personal and social, adaptive, motor, and communication domains. Twenty-eight showed delays on one domain and 21 in 3 or more. A negative correlation existed between g/day of ethanol and months of cognitive delay (-0.37, $p<0.05$). In the study, 35% had an average cognitive delay of -8.5 months (confidence interval 95% -0.5;-16.5).

For some of the study group participants, growth curves and head circumference showed normalization by 2 years.

### Conclusions

Half of the children from the Elijo Mas Sano registry had neurodevelopmental delays before the age of 2. The program of prevention and harm reduction of intrauterine exposure to alcohol and other drugs has been implemented with good preliminary results: decreased alcohol consumption during pregnancy and syndromes and alcohol-related birth defects. The next step is to follow-up to children who are at risk of fetal alcohol spectrum disorders through adolescence.

### BORN WITHOUT OH: an Innovating Model for Preventing and Managing the Neurodevelopmental Disorders Caused by Alcohol and Drug Prenatal Exposure In a Mediterranean Region

María Luisa Azurmendi Funes (1,2), Esther Tobarra Sánchez (1,2), Alberto Cárceles-Álvarez (1,2), Miguel Felipe Sánchez-Sauco (1,2), Ferrán Campillo i López (1,2), Diana Carolina Jaimes Vega (1,2), Isabel Saura Martínez (3), Almudena Pernas Barahona (1,2), Lizbeth Álvarez Gómez (4), Mayra Orozco Llamas (4), Juan Antonio Ortega-García (1,2)

(1) Pediatric Environmental Health Speciality Unit, University Hospital Virgen de la Arrixaca, Murcia, Spain
(2) Laboratory of Environment and Human Health, Instituto Murciano de Investigación Biosanitaria, Murcia, Spain
(3) Clinical Universitary Hospital Santa Lucía, Cartagena, Murcia, Spain
(4) Mount Sinai School of Medicine, New York, USA

Corresponding author:
Funes, María Luisa Azurmendi
Murcia, Spain
azurmendi_87@hotmail.com

*Background*

Born without OH is a comprehensive program of prevention, reduction of harm and management of neurobehavioral disorders associated with prenatal exposure to alcohol and other drugs through the pediatric environmental health from the beginning of pregnancy until the end of adolescence. Our objective is to prevent and reduce damage from exposure to alcohol and other drugs during critical periods of development (periconceptional until the end of adolescence).

*Methods*

Beginning in 2009, at the University Clinical Hospital Virgen de la Arrixaca (Murcia, Spain), the GREEN SHEET was implemented in hospital and primary care. It is a set of basic and concise questions for women in the 1st trimester of pregnancy to detect the environmental risk factors related to alcohol and other drugs in early pregnancy. Those with high-risk criteria of prenatal exposure to alcohol and other drugs pass to a follow-up program called ELIJO MAS SANO (CHOOSE HEALTHIER): early and comprehensive intervention for the management and monitoring of pregnant women and / or children at risk of fetal alcohol spectrum disorders (FASD).

*Results*

In the follow-up of 200 children in the program ELIJO MÁS SANO, there was a significant decrease ($p < 0.05$) in women who drank some alcohol in early pregnancy and the amount of alcohol drunk by the couple. There was a decrease in the number of newborns with congenital heart disease associated with intrauterine exposure to ethanol controlled by the number of births and abortions. There were 6 children diagnosed with fetal alcohol syndrome (FAS) (in 1500 pregnant couples), estimating a prevalence of 4

cases of FAS per 1,000 newborns in the region of Murcia.

### *Conclusions*

The Born without OH programme has been implemented with good preliminary results: reducing the consumption of alcohol during pregnancy, decreased congenital heart diseases related to intrauterine exposure to alcohol and registration of syndromes and alcohol-related birth defects.

---

### Temporal Trends of Organochlorine Compounds and Polybromodiphenyl Ethers from Utero to 4 Years of Age in the Asturias INMA Birth Cohort

Mercè Garí (1,2), Joan O. Grimalt (2), Esther Vizcaino (2,3), Berit Glomstad (2), Adonina Tardón (3,4), Ana Fernández-Somoano (4,3)

(1) Department of Earth and Ocean Dynamics, University of Barcelona, Barcelona, Spain
(2) Department of Environmental Chemistry, Institute of Environmental Assessment and Water Research, Barcelona, Spain
(3) Department of Preventive Medicine and Public Health, University of Oviedo, Asturias, Spain.
(4) Spanish Consortium for Research on Epidemiology and Public Health, Spain

Corresponding author:
Garí, Mercè
Catalonia, Spain
merce.gari@idaea.csic.es

### *Background*

Persistent organic pollutants (POPs) have diverse deleterious health effects and are persistent in the environment. Moreover, due to their lipophilic nature, they bioaccumulate in humans in utero and in the breastfeeding period. Assessment of the significance of these early accumulation processes is important for understanding what are the main mechanisms leading to the human body burden of these pollutants and for identification of the toxic stresses in the early life period.

### *Methods*

Organochlorine compounds (OCs) and polybromodiphenyl ethers (PBDEs) were measured in pregnant women (maternal serum, n=466), placental tissue (n=50), at birth (cord blood, n=323) and at 4 year-old children (serum, n=272) from a birth cohort in Asturias, Spain, sampled within the INMA (Childhood and Environment) Project. Ethical approval was granted by the Ethics Committee of San Agustin hospital, and each participant provided informed consent. To calculate the total serum burdens (in ng) for each compound and at each follow-up, total blood volumes were estimated using the body weight of each individual.

### *Results*

Body burdens at childhood were 50% to 85% lower than in their mothers, while for PBDEs, children body burdens' reached similar levels or even higher than their mothers (median percentage of 107%).

For all the compounds, the total serum burden increased from birth to 4 year-old children, although these increases were significantly higher for the PBDE compounds than for OCs. The influence of maternal breastfeeding in the accumulation of these compounds through time was also evaluated. At 4 years of age, formerly breast-fed children were found to have higher OC concentrations and total burdens than non-breastfed children. This tendency was not found for any of the PBDE compounds.

### *Conclusion*

The significance of these general trends for each individual pollutant was identified. PBDEs were found to be much more accumulated in the first years of life than OC compounds. Maternal breastfeeding was the main factor in the OC body burden increases between newborns and 4 year old children. For PBDEs, other sources of exposure rather than maternal breastfeeding may be involved.

---

### TiPP TAPP-First Steps into Life

Claudia Gerken, Yvonne Berchner, Gabriele Sadowski

Local Health Authority, Bremen, Germany

Corresponding author:
Gerken, Claudia
Bremen, Germany
Claudia.Gerken@Gesundheitsamt.Bremen.de

### *Background*

In April 2008, a home visit activity was launched as part of the social area oriented work approach “TippTapp”. This preventive program combines low-threshold health counselling for families with babies and screening on youth welfare topics.

### *Methods*

The program is currently located in disadvantaged areas of Bremen, Germany. Pediatric nurses offer to visit families and their babies at the age of 6 weeks, 6 months and 12 months.  Parents decide if they would like to take part. There is no obligation to take part in the program. Because of their regional work in outposts of the local health authority, our pediatric nurses provide specific knowledge about helpful offers in their particular region. During our visit in the families’ homes, we get a chance to discover circumstances of different origins that might influence the health and development of children. A standardized documentation sheet is used. Important development and safety topics are discussed. A differentiation of subjects mentioned by parents is important to grasp the needs of the family and to initiate appropriate help.

### *Results*

In 2014 there were visits in 46, 63% (n=2041) of 4345 invitations. Besides many helpful hints, 13 cases were reported to youth welfare. In 4 of them, help was given, although the parents did not agree.

*Conclusions*

The content of our work is transferable. The gained knowledge may be helpful in questions of urban development. Individual and general support of families can be positively influenced. In an upcoming cohort study in Bremen, TiPP TAPP is part of the set of interventions.

---

### Factors Influencing Breastfeeding Initiation

**Lizbeth Álvarez Gómez (1,2), Almudena Pernas Barahona (1,2), Miriam Espinosa Sánchez (1,2), Miguel Felipe Sánchez-Sauco (1,2), María Luisa Azurmendi Funes (1,2), Mayra Alejandra Orozco Llamas (1,2), Juan Antonio Ortega-García (1,2)**

**(1) Pediatric Environmental Health Speciality Unit, University Hospital Virgen de la Arrixaca, Murcia, Spain**
**(2) Laboratory of Environment and Human Health, Instituto Murciano de Investigación Biosanitaria, Murcia, Spain**

**Corresponding author:**
**Gómez, Lizbeth Álvarez**
**Murcia, Spain**
**almudena@pehsu.org**

*Background*

Breastfeeding is a unique and priority nutrition during the first year of life. However, its initiation and duration is determined by the interaction of multiple constitutional and environmental factors (physical, chemical, biological and psychosocial). Children are greatly vulnerable to environmental factors during the periods of pregnancy and lactation. Our aim is to study the protective and risk factors related to the initiation of breastfeeding in the Murcia Region.

*Methods*

A cross-sectional study of 99 women from the Region of Murcia, consisting of a personalized ¨face to face¨ interview with mothers at the time of hospital discharge, which were done by a nurse utilizing a carefully crafted questionnaire instrument called ¨the green page¨.

*Results*

About 90% of mothers had the intention of exclusively breastfeeding prior to giving birth, 5% partial breastfeeding, and 5% no breastfeeding. However, only 51% exclusively breastfed, 36% partially breastfed, 8% predominately breastfed, and 5% did not breastfeed. Especially during the immediate and early postpartum period, difficulties can arise such as the ones found during our study. About 27% of the mothers had problems breastfeeding, and of those mothers, 6% experienced pain in the nipple area, in 5% the newborn had issues latching on, 5% had cracks in the nipple, and 5% had irritated nipples.

*Conclusions*

Addressing multiple factors that affect breastfeeding can positively influence the initiation and duration of breastfeeding. As health professionals, we are able to promote breastfeeding and thus improving the quality of life of our children.

### Human Biomonitoring Data Analysis for Metals in an Italian Adolescents Cohort: an Exposome Approach

Dimosthenis A. Sarigiannis (1,2), Alberto Gotti (1,2), Spyros Karakitsios (1), Anna Pino (3), Gemma Calamandrei (4), Laura Chiarotti (4), Alessandro Alimonti (3)

(1) Environmental Engineering Laboratory, Chemical Engineering Department, Aristotle University of Thessaloniki, Thessaloniki, Greece
(2) Environmental Health Engineering, Scuola Universitaria Superiore IUSS, Pavia, Italy
(3) Department of Environment and Primary Prevention, Italian National Institute for Health, Rome, Italy
(4) Department of Cell Biology and Neurosciences, Italian National Institute of Health, Rome, Italy

Corresponding author:
Gotti, Alberto
Thessaloniki, Greece
alberto@eng.auth.gr

*Background*
In the framework of the Cross-Mediterranean Environment and Health Network-Life (CROME-LIFE) project, a cohort of 453 adolescents aged 13-15 years living in urban (Rome) and rural (Viterbo) areas from the Italian Programme for Biomonitoring of Exposure (PROBE) was enrolled to examine metals exposure. Children are more vulnerable to environmental stressors than adults, and have less control over their environment. Although evidence indicating the impact on children's development is growing, there is still only a partial understanding of the environmental risks to children.

*Methods*
Non-fasting blood specimens obtained by the National Microcytemia Association during the annual screening for microcytemia in schools (2009-10) were analyzed for nineteen metals by sector field inductively coupled plasma mass spectrometry. Administered questionnaires included information such as sex, diet habits, lifestyle factors and parental occupational status. Socio economic status of the family was derived by merging the educational level and occupational status of the parents. The Ethics Committee of the National Public Health Institute of Italy (ISS) approved the project.

Metal concentrations in blood were statistically evaluated. Basic statistics were used to obtain reference values where the 95th percentile describes the upper value to be used in health care and environmental policy.  Data on the study participants were geo-referenced on the basis of their residence address and the data were stored in a geo-database along with human biomonitoring data, dietary habits, environmental pollution and land cover data. In the frame of the Cross-Mediterranean Environment and Health Network LIFE+ project, an exposome-wide analysis was performed to identify the associations between metal concentration levels in blood and several exposure determinants including land cover, milk and fish consumption, both considering them individually and in combination to explore potential synergies between covariates. Ethical approval was granted by the Ethical Committee of Istituto Superiore di Sanità, Roma, Italy.

*Results*

Results show robust statistical associations of chromium with the dietary pathways analysed and land cover, showing that both out-of-region and local sources can be associated with the observed human biomonitoring levels of chromium in the population. Other robust associations were found between tungsten and co-exposure to contaminated milk and proximity to industrial activities and mercury, with a more complex interaction between co-exposure to different dietary pathways (milk and fish) coupled with proximity to industrial activities. No pathway alone was dominant, but the combined effect results in statistically significant associations with blood concentration levels of mercury. The associations of nickel with dietary pathways (co-exposure to milk and fish) and platinum with fish and industrial activities and enhanced traffic, even though statistically significant ($p < 0.05$), fail to meet the statistical robustness test. None of the other metals showed statistically significant associations with dietary patterns and/or land cover information.

*Conclusions*

All metals analyzed in this study are environmental pollutants posing high concerns with regard to their effects on human health. Their distribution in the environment is a result of natural processes and anthropogenic activities. However, in addition to environmental sources,other factors such as individual lifestyles, diet, and socio-economic factors need to be taken into account as they contribute to the levels of metals  found in the human body.

The results obtained thus far support the utility of an exposome approach to get a more accurate and holistic perspective in order to determine the association between population exposure and its determinants using human biomonitoring studies

### Early Life Exposure to Multiple Environmental Contaminants and Birth Outcomes: Pooled Analysis In Four Flemish Birth Cohorts

Eva Govarts (1), Lützen Portengen (2), Nathalie Lambrechts (1), Liesbeth Bruckers (3), Adrian Covaci (4), Vera Nelen (5), Tim Nawrot (6), Ilse Loots (7), Isabelle Sioen (8), Willy Baeyens (9), Bert Morrens (7), Roel Vermeulen (2), Greet Schoeters (1)

(1) Environmental Risk and Health, Flemish Institute for Technological Research, Mol, Belgium
(2) Division of Environmental Epidemiology, Institute for Risk Assessment Sciences, Utrecht University, Utrecht, the Netherlands
(3) Interuniversity Institute for Biostatistics and Statistical Bioinformatics, Hasselt University, Diepenbeek, Belgium
(4) Toxicological Centre, University of Antwerp, Antwerp, Belgium
(5) Provincial Institute of Hygiene, Antwerp, Belgium
(6) Centre for Environmental Sciences, Hasselt University, Diepenbeek, Belgium
(7) Faculty of Political and Social Sciences, University of Antwerp, Antwerp, Belgium
(8) Department of Public health, Ghent University, Ghent, Belgium
(9) Department of Analytical, Environmental and Geochemistry, Free University Brussels, Brussels, Belgium

Corresponding author:
Govarts, Eva
Mol, Belgium
eva.govarts@vito.be

*Background*

Prenatal chemical exposure has frequently been associated with reduced fetal growth, although results have been inconsistent. Most studies associate single pollutant exposure to these health outcomes, even though this does not reflect real life situations, as humans are exposed to thousands of pollutants during their life time. The objective of this study is to investigate the association between prenatal exposure to a mixture of environmental chemicals and birth weight.

*Methods*

We used exposure biomarker data obtained from cord blood samples of 2033 women from three Flemish birth cohorts (FLEHS I, II & III) and a regional birth cohort (3xG). The common set of exposure measures in these cohorts are the organochlorine compounds (three polychlorinated biphenyl (PCB) congeners (138, 153 and 180), hexachlorobenzene (HCB), p,p'-dichlorodiphenyldichloroethylene (p,p'-DDE)) and the heavy metals cadmium and lead. Birth weight was assessed as a proxy for reduced fetal growth. Elastic net regression was used to assess the effect of multipollutant exposure on birth weight. Ethical approval was granted by the ethical committee of Antwerp University (Hospital) and of the local hospitals.

*Results*

In the pooled database, birth weight ranged from 1245 to 5575 g with a median of 3430 g. The median contaminant levels in cord blood were: 26 ng/g lipid for PCB 153, 15 ng/g lipid for PCB 138, 18 ng/g lipid for PCB 180, 90 ng/g lipid for p,p'-DDE, 17 ng/g lipid for HCB, 0.045 µg/L for cadmium and 9 µg/L for lead. In single pollutant models, the three PCB congeners were significantly associated with reduced birth weight. The correlations between the different pollutants were low to moderate (r = 0.11 – 0.59), except for the three PCB congeners which were highly correlated with Pearson correlation coefficients ranging from 0.74-0.84. From all exposure measures, the two PCB congeners PCB 153 and PCB 180 were most consistently associated with birth weight based on elastic net regression.

### *Conclusions*

Assessing the health risk of combinations of exposure biomarkers better reflects real world situations. The findings allow for more effective risk assessment as addressing the critical chemicals in a mixture of pollutants is pivotal for risk assessment.

### *Acknowledgements*

The FLEHS studies were commissioned, financed, and steered by the Ministry of the Flemish Community. NIRAS/ONDRAF (Belgian National Agency for Radioactive Waste and enriched Fissile Material), STORA (Study and Consultation Radioactive Waste Dessel) and MONA (Mols Overleg Nucleair Afval) financed the 3xG study.

---

### Brainy Approaches to Children's Environmental Health

**Philippe Grandjean**

**(1) University of Southern Denmark, Odense, Denmark**
**(2) Harvard School of Public Health, Boston, Massachusetts, USA**

**Corresponding author:**
**Grandjean, Philippe**
**Odense, Denmark**
**pgrandjean@health.sdu.dk**

The foetus and the child are particularly vulnerable to pollution. The foetus shares the mother's exposure and accumulated body burden of pollutants, and many chemicals are transferred to the infant via human milk. In relation to body weight, a newborn or small child needs more water, energy and oxygen than an adult, and contaminant exposures can therefore be comparatively larger. In addition, during early life, cell differentiation and organ development must occur in a particular sequence and at certain times to create optimal functions of the mature organism. This vulnerability is critical in regard to the nervous system. Because there is only one chance to develop a brain, even doses that are safe for adults may, if occurring during early development, cause permanent losses of IQ. More than 200 industrial chemicals are already known to cause brain toxicity in adults. About half of these substances are produced in high volumes. All of them must be suspected to be capable of harming the developing brain. However, only a dozen industrial chemicals are recognised causes of developmental brain dysfunction. Methylmercury may serve as a paradigm, where exposures previously thought to be safe are now linked to adverse effects on brain development. However, documentation and recognition of such risks is cumbersome and takes decades, because the full impact of adverse effects may not be apparent before school age. Adding to the lack of insight, chemical substances are not systematically screened for neurotoxicity. Still, over one thousand chemicals have been reported to be neurotoxic in laboratory models, and such toxicity is therefore by no means rare. Because optimal brain function is crucial to each individual, as well as to society, new and precautionary approaches are required both for research and for decision-making on chemical testing and control.

### Psychosocial Stress and Obesity in Kaunas Children

Regina Grazuleviciene (1), Inga Petraviciene (1), Sandra Andrusaityte (1), Birute Balseviciene (2)

(1) Department of Environmental Sciences, Vytauto Didziojo Universitetas, Kaunas, Lithuania
(2) Department of Theoretical Psychology, Vytauto Didziojo Universitetas, Kaunas, Lithuania

Corresponding author:
Grazuleviciene, Regina
Kaunas, Lithuania
r.grazuleviciene@gmf.vdu.lt

*Background*
Recent research has found that family socioeconomic status, parenting stress and other environmental factors can have impacts on child development. The aim of this study was to assess the association between psychosocial stress in the family and risk for obesity among 4–6 year old children.

*Methods*
This nested case-control study included 1,489 Kaunas Cohort Birth Study children followed-up for 4–6 years. We used the Parent-Child Dysfunctional Interaction subscale of the Parenting Stress Index (short form) and questionnaires on child development to assess risk factors for children obesity. The body mass index status for age was calculated and the risk factors for being overweight/obese were estimated. Logistic regression models were fitted to examine the associations as odds ratios (OR) between the parent-child relationship and the risk of overweight, adjusted for relevant covariates. The Kaunas Pregnant Women Birth Cohort had 3 ethical approvals conducted by the Lithuanian Bioethics Committee.

*Results*
A total of 6.8% of the children were overweight and 5.4% were obese. Children from smoking families with lower education faced almost double the risk of obesity. The stratified multivariate model showed that, with reference to the group of high educated and normal parent-child relations, low educated and pathological parent-child relationships were statistically significant risk factors for overweight children of 4–6 years old (OR 2.43; 95% confidence interval 1.31–4.51).

*Conclusions*
Maternal tobacco smoking and pathological parent-child relations may be predictors of overweight and obesity throughout the developmental trajectory of childhood. Measures to quit smoking and psychosocial stress management should be encouraged among pregnant women to decrease their children's obesity risk.

---

### Influence of Gestational Weight Gain on the Transfer of Organochlorine Pollutants to Infants

Joan O. Grimalt (1), Esther Vizcaino (1), Marta Fort (1), Berit Glomstad (1), Ana Fernández-Somoano (2), Loreto Santa Marina (3), Jesús Ibarluzea (3), Adonina Tardón (2), Jordi Sunyer (4)

(1) Spanish National Research Council, Barcelona, Spain
(2) Department of Preventive Medicine and Public Health, University of Oviedo, Asturias, Spain
(3) Public Health Branch of Gipuzkoa, The Basque Government's Health Department, Donostia-San Sebastián, Spain
(4) Centre for Research in Environmental Epidemiology, Barcelona, Spain

Corresponding author:
Grimalt, Joan
Barcelona, Spain
joan.grimalt@idaea.csic.es

*Background*
Transplacental transfer and breastfeeding are the main transport routes of organic pollutants to children at the beginning of life. These transmission mechanisms primarily depend on the maternal pollution burden, but its impact may be modulated by physiological effects. We found that gestational weight gain (GWG) exerts a considerable influence on the content of hydrophilic and low volatile pollutants in cord blood and breast milk.

*Methods*
We measured levels of 14 organochlorine pesticides, 7 polychlorobiphenyls and 14 polybromodiphenyl ethers (PBDEs). Persistent organic pollutant neonatal concentrations were inversely associated with GWG after adjustment for age, pre-pregnancy body mass index, educational level, and fish consumption. On average, neonates of women with high or recommended GWG as defined by the Institute of Medicine had lower persistent organic pollutant concentrations than neonates of mothers with low GWG. This study was approved by the ethics committees of the Clinical Research Ethical Committee of the Municipal Institute of Health Care, the Ethics Committee of the Donostia Hospital and San Agustin Hospital, and informed consent was provided by every participant.

*Results*
Colostrum from mothers with low GWG also had significantly higher concentrations of polychlorobiphenyls and 4,4'-dichlorodiphenyldichloroethylene than colostrum from mothers who gained weight within Institute of Medicine recommendations or in those who exceeded this threshold. Statistically significant differences were also found in the colostrum: maternal serum ratios of these compounds. Women with low GWG retained higher pollutant amounts in colostrum.

*Conclusions*

The higher mobilization of stored organic pollutants observed in pregnant women with low GWG plays a role for in utero pollutant exposure and during breastfeeding. The present findings suggest an association between infant exposure to persistent organic pollutants and inadequate GWG, encouraging pregnant women to meet the recommended Institute of Medicine guidelines to reduce the accumulation of these pollutants in newborns and during breastfeeding.

---

### Environmental Health Issues for Spain

Joan O. Grimalt

(1) Spanish National Research Council, Barcelona, Spain

Corresponding author
Grimalt, Joan
Barcelona, Spain
jgoqam@cid.csic.es

*Background*

In general, children in Spain have the same environmental health conditions as other European children. However, they undergo some specific pollution stresses that must be considered for the implementation of adequate public health policies.

*Methods*

This presentation combines relevant information from the scientific literature and the results of the studies developed at the IDAEA.

*Results*

One of these stresses concerns mercury exposure. With the only exception of Portugal, the concentrations of this metal in hair from children living in Spain are considerably higher than in children from other European countries, and the differences are strong. In Spain, the high mercury content observed in children's hair, and also cord blood, is rather uniform independently of location. These high mercury concentrations are likely related with high fish and seafood consumption. Often, Mediterranean fish sold in Spanish market places, both oily and lean fish, contain mercury concentrations above the EU regulations for human consumption. Impaired intellectual development due to mercury exposure has been observed in children from Granada, but not in children from other Spanish cohorts. Another specific pollution stress of children living in Spain is exposure to hexachlorobenzene. Examination of cord blood serum concentrations of this compound shows higher levels than in European children. In this case, the differences are significant when comparing newborns from different Spanish locations. In utero exposure to hexachlorobenzene has been observed to be related with lower birth size. It has also been associated with higher incidence of overweight, alteration of thyroid hormones, attention deficit hyperactive syndrome and poorer social behavior in four-year-old children from Spanish cohorts.

Cotinine content, and therefore exposure to tobacco smoking, is also high among Spanish newborns, although when compared with other European cohorts, the difference is not as strong as in the previously mentioned pollutants. Exposure to polychlorobiphenyls, hexachlorocyclohexanes, dichlorodiphenyltrichloroethane/dichlorodiphenyldichloroethylene, perfluorohexane sulfonate, perfluorooctane sulfonate, perfluorooctanoate, perfluorononanoate, bisphenol A, polybromodiphenyl ethers or cadmium in children from Spanish cohorts is similar to that observed in other European countries.

In recent studies, lower development of working memory and higher inattentiveness has been observed in children from Barcelona exposed to high levels of traffic pollutants, namely nitrogen dioxide, fine aerosol particles and polycyclic aromatic hydrocarbons. These effects are probably common in all children living in urban areas under strong traffic pollution.

### *Conclusions*

In the context of health conditions that are comparable to those of other EU children, the specific pollution stresses described in this review deserve specific attention from the health authorities.

---

### Environmental Exposure and Neurodevelopment in the Mothers' and Children's Environment and Health Study

Mina Ha (1), Eun-Hee Ha (2)

(1) Department of Preventive Medicine, College of Medicine, Dankook University, Cheonan, South Korea
(2) Department of Occupational and Environmental Medicine, Ewha Medical Research Center, School of Medicine, Ewha Womans University, Seoul, South Korea

Corresponding author:
Ha, Eun-Hee
Seoul, South Korea
eunheeha@ewha.ac.kr

### *Background*

The Mothers and Children's Environment and Health study is a prospective multicenter cohort study of pregnant women with follow-up of their offspring in Korea.

*Methods*

During 2006 to 2010, 1,751 mother and child pairs were recruited. Various environmental factors including biomarkers in blood and urine and health outcomes including neurodevelopment and allergic diseases were checked throughout periods of childhood. The Korean Bayley Scale of Infant Development, 2nd edition was performed at 6, 12, 24, and 36 months of age, and intelligence quotient was measured using the Korean version of the Wechsler Preschool and Primary Scale of Intelligence, revised edition and Korean - Wechsler Intelligence Scale for Children IV at 62 months and after.

*Results*

We found significant effects on children's neurodevelopment in association with maternal exposure to lead, iron, manganese, cadmium, phthalates, secondhand smoke, air pollutants such as nitrogen dioxide and $PM_{10}$, as well as maternal stress and depressive symptoms, job strain during pregnancy, and postnatal breastfeeding duration.

Two preliminary studies suggested an association with prenatal lead, cadmium, and iron exposure with children's cognitive development at 5 years of age. Currently, children in the Mothers and Children's Environment and Health study are between 7 to 10 years old, and are being measured on their social responsiveness and behavior using the Social Responsiveness Scale, Attention Deficit Hyperactivity Rating Scale and Child Behavior Checklist. Future analyses should consider multiple exposure factors, times of exposure, and various dimensions of neuro- behavioral- cognitive- development.

*Conclusions*

A new nationally representative birth cohort study, the Korean Children's Environmental Health Study, was initiated in 2015. Follow-up will be conducted at every growth stage for the following 17 years based on the experiences of the Mothers and Children's Environment and Health study, which is expected to provide statistically powerful and generalizable evidences in environmental and children's health.

---

### Children's Exposure to Endocrine Disrupting Chemicals. 'Women in Europe for a Common Future - WECF' is Working with Physicians, Scientists, NGOs and Media For Better Policy and More Protection

**Johanna Hausmann**

**Women in Europe for a Common Future, Munich, Germany**

**Corresponding author:**
**Hausmann, Johanna**
**Munich, Germany**
**johanna.hausmann@wecf.eu**

*Background*

Studies show that Endocrine Disrupting Chemicals (EDCs) play a role in disrupting human brain development, deterioration of male and female reproductive health, increased incidence of male and female hormone related cancers, and the increase in cardiovascular disease, obesity and diabetes. The most vulnerable groups are pregnant women, fetuses and children. Better regulation is urgently needed. The EDC criteria to classify EDCs for being banned/regulated under the EU pesticides and biocides regulations proposed by the European Commission in June this year are negligible, not protective and ignore precautionary principles. Since the EDC criteria should function as a blueprint for other EU regulations and probably even in countries outside the EU, pressure from all public interest groups is urgently needed. The WHO calls the ubiquitous presence of EDCs in daily products and food a "global threat". Scientists and scientific associations like FIGO and the Endocrine Society are calling for political action for better protection as well as on action from doctors on awareness raising. The voice of scientists and doctors is needed more urgently than ever.

*Methods*

Women in Europe for a Common Future is active on a national and European policy level around EDCs and on an international level within the framework of the United Nations Environment Programme chemical policy process Strategic Approach on International Chemicals Management (SAICM) to achieve better protection of children from EDC exposure. Within several projects, WECF works on different target groups to raise awareness, improve the legal situation and give concrete information to consumers. Together with other NGOs (www.edc-free-europe.org, www.ipen.org/search/node/EDCs ), WECF aims to increase the pressure for policy makers to take responsibility in protecting human health and the environment from EDCs.

*Results*

WECF informed thousands of people who work and live with children about hazardous chemicals in products and ways to reduce the exposure via its Nesting programme  (www.projetnesting.fr ). It delivers websites in several languages, a mobile phone app, workshops for parents and health and educational experts and information materials. On a policy level, WECF advocates for better regulations and for their enforcement.

*Conclusions*

The presentation aims to give an overview of recent EDC policy developments, EDC awareness raising and media activities, and scientific advances around EDCs, as well as political action taken so far. We will also show how strengthened cooperation between NGOs, physicians, media and policy makers is possible.

### Ambient Air Pollution and the Onset of Asthma, Hay Fever, and Allergic Sensitization in Birth Cohorts: A Systematic Review and Meta-Analysis

Joachim Heinrich

Institute of Epidemiology, Helmholtz Zentrum München, German Research Center for Environmental Health, Munich, Germany

Corresponding author:
Heinrich, Joachim
Munich, Germany
joachim.heinrich@med.uni-muenchen.de

*Background*

The causal role of ambient air pollution on the onset of asthma and allergic conditions continues to be debated. We provide a systematic review and meta-analysis of birth cohort studies with follow-ups from birth to adolescence.

*Methods*

In a previous systematic review by Bowatte et al. (2015), 19 birth cohort studies published from 1960 to March 2014 were identified. Using the same methodology, we extended this search up to January 2016, which allowed several key new studies to be considered. Random effects meta-analysis was used to obtain combined estimates.

*Results*

A total of 338 references published between April 2014 and January 2016 were identified. Of these, 88 were duplicates and 216 studies did not meet the inclusion criteria. Manual searches identified 4 additional papers. These new studies were added to those previously identified by Bowatte et al. (2015). Ultimately, 27 eligible papers were included in the systematic review, 15 of which could be used in meta-analyses.

Combined associations between asthma with particulate matter ($PM_{2.5}$ (1.11 (95% confidence interval 0.97, 1.26) per 2 µg/m$^3$ increment) and nitrogen dioxide (1.08 (95% confidence interval 0.96, 1.20) per 10 µg/m$^3$ increment) were not significant. Also for wheeze, the combined association was not significant neither for $PM_{2.5}$ (1.13 (95% confidence interval 1.00, 1.28) per 2 µg/m$^3$ increment) nor for nitrogen dioxide (1.08 (95% CI 0.98, 1.18) per 10 µg/m$^3$ increment). None of the combined associations with allergen sensitization or hayfever reached statistical significance. There were too few birth cohort studies on eczema to conduct a meta-analysis.

*Conclusions*

Despite the existence of a wealth of high quality, large, longitudinal birth cohort studies, the epidemiological evidence supporting an association between traffic-related air pollution with asthma and other allergic health outcomes remains insufficient to confirm a causal association.

### Personal Exposure to $PM_{2.5}$ and Benzo[a]pyrene in Ostrava, Czech Republic

Vlasta Svecova (1), Ivo Solansky (1), Thomas Cole-Hunter (2), the CITI-SENSE Consortium (3), Radim J Sram (1), Alena Bartonova (4)

(1) The Institute of Experimental Medicine, Academy of Sciences of the Czech Republic, Prague, Czech Republic
(2) Centre for Research in Environmental Epidemiology, Barcelona, Spain
(3) CITI-SENSE, Norwegian Institute for Air Research, Oslo, Norway
(4) Norwegian Institute for Air Research, Oslo, Norway

Corresponding author:
Honkova, Katka
Czech Republic
katerina.honkova@biomed.cas.cz

*Background*

Benzo[a]pyrene (B[a]P) is a proven human carcinogen. In the air, it is associated with particulate matter ≤ 2.5 μm ($PM_{2.5}$). High concentrations of both compounds found in the Ostrava region, CZ, have led to concern in the local population. To support the local needs for a communication platform, we started a case study of the CITI-SENSE project "Citizen Observatories for Quality of Life", with a special study on personal exposure and monitoring in schools.

*Methods*

Twenty non-smoking adult volunteers were recruited in the most polluted part of Ostrava to participate in three biomonitoring campaigns. All participants completed a personal questionnaire and time-location-acitivity diary. High precision personal monitors for $PM_{2.5}$ were used next to the new sensor technology for NOx assessment. Outdoor concentrations of $PM_{2.5}$ and B[a]P were measured using a high volume air sampler.

*Results*

The campaigns were held in winter 2013, summer 2015 and winter 2016. Average two-day personal exposures to $PM_{2.5}$ were 68.8, 45.3 and 37.4 μg/m$^3$, respectively. In 2013 and 2015, personal exposures to B[a]P were 5.5 and 1.3 ng/m$^3$. The outdoor concentrations for 2013 and 2015 were 35.4 and 17.6 μg/m$^3$ of $PM_{2.5}$ and 5.3 and 1.2 ng/m$^3$ for B[a]P in two weeks period during the campaigns. The highest concentration of B[a]P was found in kindergarten Ostrava Radvanice in February 2016 of 1.8 indoor and 5.9 ng/m$^3$ outdoor.

*Conclusions*

With use of low-cost sensors, we created a Citizens' Observatory to boost the cooperation between scientists, citizens and other stakeholders, to increase opportunities for environmental health governance. To improve relevance to the local population, we combined low-cost online measurements with high precision instumentation, and with measurements and information campaigns in schools, all in cooperation with city authorities. Such activities provide the context necessary for communicating the results of the biomonitoring study to citizens as well as authorities.

### Minamata Convention on Mercury and Vulnerable Populations

Yuyun Ismawati (1,4), Stephan Bose-O'Reilly (2,3)

(1) Center for International Health, Ludwig-Maximilians-Universität München, Munich, Germany
(2) University Hospital Munich, Institute and Outpatient Clinic for Occupational, Social and Environmental Medicine, WHO Collaborating Centre for Occupational Health
(3) University for Health Sciences, Medical Informatics and Technology, Department of Public Health and Health Technology Assessment, Institute of Public Health, Medical Decision Making and Health Technology Assessment, Austria
(4) BaliFokus Foundation, Bali, Indonesia

Corresponding author:
Ismawati, Yuyun
Bali, Indonesia
Yuyun.Ismawati@lrz.uni-muenchen.de

*Background*

The United Nations Environment Programme's global mercury assessment in 2013 identified artisanal and small-scale gold mining (ASGM) as the largest single sector releasing mercury emissions from intentional use of mercury. The United Nations Industrial Development Organization identified artisanal and small-scale gold mining practices using mercury in more than 50 countries, many of them low- and middle-income countries. Very often, gold extraction processes using mercury occur within residential areas, polluting villages and exposing residents, including children, pregnant women and women of child bearing age. This mercury exposure leads to adverse health effects to the cognitive and neurological development of affected children. The long-term impact of mercury pollution could lead to high socio-economic costs, fewer jobs and economic opportunities, and increased family burden and need for health care.

*Methods*

Observations conducted during the inter-governmental negotiation (INC) meetings to prepare a global legally binding instrument on mercury and literature review.

*Results*

The Minamata Convention on Mercury is a global environmental health agreement, signed by 128 countries in October 2013 and currently under ratification. The treaty's objective is to protect human health and the environment from anthropogenic releases of mercury and mercury compounds.

The mercury treaty specifically provides for and highlights the need to protect human health. Special attention to health aspects addressed in Article 16 aims to promote the development and implementation of strategies and programmes to protect populations at risk, particularly vulnerable populations. Among the most important requirements of the treaty is that information related to mercury and human health must not be kept confidential, thereby highlighting the public 'Right to Know' about mercury impacts on their health.

*Conclusions*
Article 16 of the Minamata Convention on Mercury does not contain mandatory provisions, but it encourages parties to the treaty to promote a range of health related measures. In addition to the present study, health issues also addressed in other articles related to ASGM, mercury in products, emissions and releases, contaminated sites, awareness and education as well as information exchanges are discussed. Countries with ASGM activities are recommended to develop a public health strategy with regard to the exposure of artisanal and small-scale gold miners and their communities to mercury. Such strategies should be included in the national action plan after the respective country ratifies the treaty.

---

### Birth Weight In Relation to Cord Blood Levels of Selected Elements In Slovenian Mother-Child Pairs

Marta Jagodic (1,2), Janja Snoj Tratnik (1,2), Vesna Fajon (1,2), Darja Mazej (1), Mladen Krsnik (3), Alfred B Kobal (4), Joško Osredkar (3), Alenka Sešek Briški (3), Milena Horvat (1,2)

(1) Jožef Stefan Institute, Ljubljana, Slovenia
(2) Jožef Stefan International Postgraduate School, Ljubljana, Slovenia
(3) Institute of Clinical Chemistry and Biochemistry, University Medical Centre Ljubljana, Ljubljana, Slovenia
(4) Ex-Department of Occupational Health, Idrija Mercury Mine, Idrija, Slovenia

Corresponding author:
Jagodic, Marta
Ljubljana, Slovenia
marta.jagodic@ijs.si

*Background*
The purpose of the present study was to evaluate the association between cord blood (CB) and CB serum levels of selected toxic and potentially toxic elements (manganese, copper, zinc, arsenic, selenium, cadmium, lead, mercury, magnesium, calcium and iron (III)) on birth weight in Slovenia, taking into account maternal socio-demographic characteristics and dietary habits due to various associations obtained in literature.

*Methods*
Delivering women (18-45 years) were recruited primarily from the central part of Slovenia as part of the Public Health Impact of Long-Term, Low-Level Mixed Element Exposure in Susceptible Population Strata study (PHIME) (2007-2011). CB samples were collected at delivery. Associations between birth weight and a) predictors obtained through the questionnaires and b) levels of the selected elements in CB samples were tested using univariate and multivariate analyses.

*Results*

In general, babies had normal birth weight (GM=3400 g, 95% CI: 3348–3453 g) and levels of selected elements did not represent a health risk for the mother-child pairs. Linear regression analyses showed statistically significant positive correlations between birth weight and number of children in the family, maternal pregnancy problems, pregnancy body mass index of mothers, gestational age, level of maternal education; child's gender (boys were heavier) and negative correlations between birth weight and the number of bedrooms in the home. Among the elements tested, birth weight showed significant positive correlation with calcium in CB serum and negative association with iron (III) in CB serum.

*Conclusions*

The main predictors of the newborn's weight (length of baby, gender of the baby, gestational age, pregnancy and pre-pregnancy body mass, level of education) were expected, while the associations between birth weight and essential elements (calcium and iron (III)) in the Slovenian birth cohort should be evaluated to identify possible mechanisms/explanation for the observed associations.

---

### Reality of Children's New Environment and Future Policy Needs

Marija Jevtic (1), Catherine Bouland (2)

(1) University in Novi Sad, Faculty of Medicine, Institute of Public Health of Vojvodina Novi Sad, Serbia
(2) Université libre de Bruxelles (ULB), School of Public Health, Research Center on Environmental Health and Occupational Health, Brussels, Belgium

Corresponding author:
Jevtic, Marija
Novi Sad, Serbia
marija.jevtic@uns.ac.rs

*Background*

As professionals, we are confronted with increasing challenges in the fields of environment and health. Attempting to find the best way to solve numerous problems, new disciplines have been established. How can we make efficient policy to reduce risks and prevent harmful scenarios in the future? Some of us will still be alive and children that are five years old today will have their own young children in 2050. Besides knowledge, do we have the courage and imagination for an effective holistic approach to produce successful policy to preserve the environment for our children?

*Methods*

Based on the premise that students are the decision makers of the future, this research examines and analyzes (using a questionnaire) students' opinions about public health priorities in the field of environment and health.

*Results*

The results present the initial phases of data collection and consider priorities in public health, especially in connection to the environment. Preliminary results point to the necessity of giving priority to climate change, water safety, and other environmental challenges. The challenge of a fast changing environment due to climate changes is probably the most important. Children are growing up in different environments, full of risk as consequences of climate changes and different disasters, as well as human migration, homelessness, discontinuation of schooling, unsecure health protection, etc.

### *Conclusions*

Future policy should be comprehensive, foster adaptation and mitigation activities and aim to include children all along their education. Policy should be established by multi-sector stakeholders, in collaboration between education and health, with the primary aim to decrease health consequences on children, protect children's health and the whole population, now and in the future. We need a new approach to prepare students, children, professors and families for climate changes, and provide them with knowledge to raise their awareness. We should increase capacity to overcome all these rapid changes in the environment now, and prepare children for the future.

---

### Association Between Breastfeeding Duration and Cognitive Development, Autistic Traits and ADHD Symptoms: a Multicenter Study in Spain

Jordi Julvez (1,2)

(1) Barcelona Institute for Global Health, Centre for Research in Environmental Epidemiology, Barcelona, Spain
(2) Barcelona Biomedical Research Park, Barcelona, Spain

Corresponding author:
Julvez, Jordi
Barcelona, Spain
jordi.julvez@isglobal.org

### *Background*

Several studies have related longer breastfeeding durations to better intellectual performance in children. However, whether this relationship is entirely attributable to socio-environmental factors remains under debate. Few studies have investigated the potential protecting effects of breastfeeding against behavioral problems such attention deficit hyperactivity disorder (ADHD) symptoms, and even fewer on autism spectrum disorders (ASD) traits, and the extent to which these effects are mediated by cognitive function has never been explored.

### *Methods*

This study examines the association between breastfeeding duration and cognitive development, attention, ADHD symptoms, and autistic traits using data from the Infancia y Medio Ambiente – Environment and Childhood – Project, a Spanish multicenter birth-cohort study, taking into account the intensity of breastfeeding. Duration of overall, predominant, and exclusive breastfeeding was documented during infancy through maternal questionnaires. Children (N = 1,832; mean age = 4.8 years)

were assessed for cognitive development (McCarthy Scales of Children's Abilities), attention (Conners' Kiddie Continuous Performance Test), ADHD symptoms (criteria of the DSM-IV form list), and autistic traits (Childhood Autism Spectrum Test). Ethical approval was granted by the Clinical Research Ethical Committee of the Parc de Salut Mar.

*Results*

Several potential confounding factors were documented through parental interviews. After adjustment for confounders, longer duration of breastfeeding was associated with better cognitive development, reduced ADHD symptoms, and lower autistic traits. The latter effects appeared independent from each other.

*Conclusions*

This study provides further confirmation of a positive association with cognitive function over socio-environmental factors, and also suggests a protecting role against ADHD symptoms and autistic traits. Results are in agreement with recommendations of prolonged breastfeeding durations to promote child development.

### Occupational Noise Exposure During Pregnancy — New Aspects in Children's Language Acquisition

Soile Jungewelter (1), Markku Sallmén (1), Erkko Airo (2), Marja-Liisa Lindbohm (1), Helena Taskinen (1), Minna Huotilainen (3), Eira Jansson-Verkasalo (3)

(1) Section of Dermatology, Finnish Institute of Occupational Health, Helsinki, Finland
(2) (Tech) Section of Dermatology Finnish Institute of Occupational Health, Helsinki, Finland
(3) University of Turku, Turku, Finland

Corresponding author:
Jungewelter, Soile
Helsinki, Finland
soile.jungewelter@ttl.fi

*Background*

Occupational noise in pregnancy may affect the child´s central auditory processing and language acquisition. There are no investigations on this topic. At present, work safety focuses on protecting maternal hearing.

*Methods*

We conducted a cohort study among women working in the food industry, and as kindergarten teachers, musicians, dental nurses or pharmacists. We contacted 855 women with new-born children, 578 (68%) of which responded to baseline and 423 (73%) when their children were 12 months old. The final analyses included 406 mother-child pairs.

The baseline questionnaires focused on maternal occupational exposure and family factors. Language acquisition was measured by a validated questionnaire, Esikko, evaluating social communication, speech production and language comprehension. The mother filled out the questionnaire when her child was 12 months old. An occupational hygienist assessed noise exposure on the basis of maternal profession, job tasks and noise measurements using the categories: no exposure (<70 dB, n=178), low exposure (>=70 dB<=80 dB, n=108), and moderate/high exposure (>80 dB, n=120). We used linear regression, adjusted for mother's age, employment, child's gender and father's education to evaluate the association between noise exposure and children's language acquisition. Ethical approval was granted by the Ethics Committee of The Hospital District of Helsinki and Uusimaa.

*Results*

The adjusted mean language acquisition scores (range 0–57) were 34.3 (95% confidence interval (CI) 32.5–36.0) for no exposure, 34.8 (32.6–36.9) for low noise exposure, and 34.9 (32.8–37.0) for moderate/high exposure. There were no statistically significant differences among the exposure groups. This was also true in analyses of social communication, speech production and language comprehension among food industry workers' children. Noise exposure was suggestively associated with lower language acquisition scores among kindergarten teachers' children. Reading to the child was strongly associated with high language acquisition scores in the entire data.

*Conclusions*

We found no clear association between noise exposure during pregnancy and language acquisition among one-year-old children. The study provides further results concerning two-year old toddlers.

### Persistent Pollutants in Food Items from Menorca Island

**Eva Junqué (1), Anna Arce (1), Joan O. Grimalt (1), Maties Torrent (2), Jordi Sunyer (3)**

**(1) Institute of Environmental Assessment and Water Research, Spanish National Research Council, Barcelona, Spain**
**(2) Àrea de Salut de Menorca, Servei de Salut de les Illes Balears, Menorca, Spain.**
**(3) Barcelona Institute for Global Health, Centre for Research in Environmental Epidemiology, Barcelona, Spain**

**Corresponding author:**
**Junqué, Eva**
**Barcelona, Spain**
**ejmqam@cid.csic.es**

*Background*

health effects. Organochlorine compounds are synthetic products used in industrial and agricultural applications, whereas the environmental occurrence of mercury has been largely enhanced by human activities. Despite their different structure, these compounds share common properties such as high toxicity (several of them), persistence in the environment and are widely distributed on the planet.

Furthermore, they bioconcentrate and biomagnify along the food chain. Dietary intake is therefore a major route of incorporation of these compounds in most human populations. However, it is important to ascertain what specific food items are the most relevant. This is particularly important for children since their organs and metabolism are still developing.

Body burden of dichlorodiphenyltrichloroethane and mercury in children from Menorca Island (western Mediterranean Sea) were considerably high when compared with cohorts from continental Europe (INfancia y Medio Ambiente and Cross-Mediterranean Environment and Health Network).

*Methods*

The present study aimed to determine the content of organochlorine compounds and mercury in representative food items from Menorca Island. Foodstuffs including seafood, meat, vegetables, fruits, cheese and eggs were collected in 2014. Fish were captured by local fishermen and all other food stuffs were produced locally.

Organochlorine compounds analysis for the determination of pentachlorobenzene, hexachlorobenzene, hexachlorocyclohexanes, dichlorodiphenyltrichloroethaneand metabolites (dichlorodiphenyldichloroethane and dichlorodiphenyldichloroethylene) and polychlorobiphenyls were performed by gas chromatography–electron capture detector and gas chromatography–mass spectrometry. Determination of mercury was performed by inductively coupled plasma mass spectrometry.

Organochlorine compounds analysis for the determination of pentachlorobenzene, hexachlorobenzene, hexachlorocyclohexanes, dichlorodiphenyltrichloroethaneand metabolites (dichlorodiphenyldichloroethane and dichlorodiphenyldichloroethylene) and polychlorobiphenyls were performed by gas chromatography–electron capture detector and gas chromatography–mass spectrometry. Determination of mercury was performed by inductively coupled plasma mass spectrometry.

*Results*

Seafood was observed to be the foodstuff with highest organochlorine compounds and mercury levels. The highest organochlorine compound concentration in fish was found in european hake (6.14 ng/g fresh weight of 4,4'-dichlorodiphenyldichloroethylene) and in shellfish, the mussel specie (1.83 ng/g fresh weight of polychlorinated biphenyl 153). Fruits and vegetables presented the lower concentrations. Concerning mercury, most fish samples exceeded the maximum level fixed by the European Commission (0.5 and 1.0 mg/kg fresh weight). Small-spotted catshark presented the highest concentration with 3.77 mg/kg fresh weight.

*Conclusions*

Low concentrations of many organochlorine compounds found in the present study suggest that the population has not been exposed to these compounds. However, high mercury levels evidence that there is a risk for children living in the Mediterranean Basin if they are consumers of large amounts of local seafood.

### Study of Childhood Illness for the Period 2009—2011, Meeting the City: Sofia and Pleven with a View to Effective Health Promotion

Nevena Tzacheva (1), Galya Traykova (1), Pepa Karadzhova (2)

(1) Department of Occupational Medicine, Faculty of Public Health, Medical University-Sofia, Sofia, Bulgaria
(2) Medical University of Pleven, Pleven, Bulgaria

Corresponding author:
Karadzhova, Pepa
Pleven, Bulgaria
karadzhova@yahoo.com

*Background*
Health status is an important aspect of children for their physical and mental development and their future career. It is determined by a number of factors, including family and school environment, the organization of school health and environmental factors. These determinants are reflected in the incidence of turnover registered by their personal physicians.

*Methods*
We collected official data from the Capital District Health Inspection, SRZI, and regional health inspection in the city of Pelven over a two-year period from 2009–2011.

*Results*
Pleven, Bulgaria has a high degree of respiratory diseases, infectious diseases, eye diseases, diseases of the digestive system, diseases of the ear and injuries.

*Conclusions*
We believe that appropriate programming for targeted health promotion specifically identifying health problems in connection with leading environmental factors would be beneficial.

### Potential Environmental Hazards to Child Health in a Prospective Mining Area in North Sumatra, Indonesia

Muchtaruddin Mansyur, Levina Chandra Khoe, Muhammad Ilyas

Department of Community Medicine, Faculty of Medicine, Universitas Indonesia, Jakarta, Indonesia

Corresponding author:
Khoe, Levina Chandra
Jakarta, Indonesia
levinachandramd@gmail.com

*Background*
Globally, Indonesia is well known for its natural environment and rich resources of potential mining areas. However, a number of environmental health issues have arisen due to mining activities. Human exposure could lead to various health impacts, such as kidney damage, cancer, child development, and other toxic effects. Children are most vulnerable to various heavy metal intoxications, especially in the first five years of life, since this is the critical period for brain and physical development. This study aimed to understand the potential environmental hazards in a prospective mining area in North Sumatra and to assess the heavy metals concentration on children's blood.

*Methods*
This is a cross-sectional study conducted in areas which may potentially be affected by future mining activities. The survey covered ten villages living in the 30 kilometer radius of the mining areas. Father-child pairs were recruited, with male adults aged 19-55 years and a young child 24-59 months old. Surveys were conducted to gather data on environment, food market, and diet patterns. Assessment of heavy metals concentration in blood was performed. Ethical approval was granted by the Committee of the Medical Research Ethics, Faculty of Medicine, University of Indonesia.

*Results*
The environmental survey showed the heavy metals concentration were within allowable range as sources for drinking water, while mercury and zinc level in soils were high. The mean capillary blood lead levels in children were 7.17 ± 0.23 μg/dL, whereas those of fathers were 6.01± 2.89 μg/dL. The blood concentrations of arsenic, copper, zinc, cadmium, and mercury were in the acceptable range for both children and fathers. The mean hemoglobin level of children and fathers were 11.72 ± 1.19 g/dL and 15.08 ± 1.50 g/dL, respectively. No correlation was found between lead and hemoglobin level.

*Conclusions*
This study showed that the heavy metals concentration in the environment and human body were within tolerable limits. However, further research should be done when mining activities take place in the area.

---

### Minamata Convention on Mercury and Children's Environmental Health: Study on Mercury-Added Consumer Products Use in Pakistan

**Mahmood A. Khwaja**

**Sustainable Development Policy Institute, Islamabad, Pakistan**

**Corresponding author:**
**Khwaja, Mahmood A**
**Islamabad, Pakistan**
**khwaja@sdpi.org**

*Background*
Children are at high risk of developing neurodevelopmental disorders that affect the brain and nervous system, including autism, attention deficit hyperactivity disorder, intellectual disabilities, and other learning and behavioral disabilities. Thousands of chemicals are used in material production processes (nearly 100,000 in the EU) that have not been adequately assessed for human and environmental impacts, and yet are used daily in consumer products. There is ample scientific evidence to support a link between children's risks for neurodevelopmental disorders and toxic chemicals, such as mercury, in consumer products, food, air and water.

Mercury ranked 3rd in the hazardous list of toxic substances, and it is a pollutant of increasing concern, as it is not only toxic to humans, but also to wildlife and the environment. In view of mercury and mercury-added products' toxicity and hazardousness, in 2013, under the United Nations Environment program, the Minamata Convention on Mercury was enacted by over 140 countries, already signed by 128 countries (including Spain & Pakistan) and ratified by 28 countries (including USA). The main objective of this convention is to protect human health and the environment from emissions and releases of mercury and mercury compounds. Articles 4 & 16 of the convention deal, respectively, with mercury added products (including dental amalgam & skin lightening/tanning creams) and health aspects (to protect population at risk, particularly vulnerable populations – children, pregnant women & others).

### *Methods*

Over the past few years, the Sustainable Development Policy Institute has been actively involved in the negotiations process leading to the Minamata Convention on Mercury, as well as research on the health and environmental effects of the use of mercury-added products, such as mercury dental amalgam and skin lightening/tanning creams in Pakistan. Standard analytical methods were used for the measurement of mercury in air and semisolid skin lightening creams. For opinion sharing, interviews based on developed questionnaires were held with dental professionals and dermatologists/skin specialists.

### *Results*

Measurements at 34 dental sites indicated mercury vapors levels in outdoor/indoor air in the range 109 – 26,000 ng/m$^3$ for air (against reference standard = 300 ng/m$^3$). Survey work/interviews at 38 dentistry teaching institutions in the country and over 90 private dental clinics in 3 main cities showed 90% of the respondents supported review and revision of the current curriculum and inclusion of additional contents on dental mercury amalgam related to health, waste issues and mercury free dentistry, using mercury free material for dental restoration. Of the interviewed dental professionals, 70% supported mercury free material use for dental restoration as the first choice for pregnant women and children (under 12 years age) and 67% supported a ban on mercury dental amalgam use. Chemical analyses of 20 samples (others in progress) of different skin lightening creams brands revealed alarmingly high total mercury content in some of the analyzed samples. On the basis of total mercury contents, the studied skin whitening creams have been categorized into 4 groups: most, (mercury > 10,000 ppm), highly (mercury > 5000), moderately (mercury < 5000) and least (mercury < 1 ppm) hazardous skin lightening creams.

### *Conclusions*

Our research findings provide ample evidence of most likely mercury exposure to personnel (especially children) at dental sites and due to the use of skin lightening creams containing high levels of mercury content. We strongly recommend (i) mercury specific legislation for public/consumers daily use products, (ii) review and revision of dentistry & dermatology curriculum at medical institutions and (iii) ban on the production/use of skin lightening creams with total mercury content above one ppm (as per Minamata convention on mercury requirement) and in the first phase, use of mercury amalgam for dental health restoration of pregnant women and children below the age of 12 years. In this regard, our awareness & advocacy activities and discussion with the stakeholders (including government) are in progress.

### Children and Artisanal and Small-Scale Gold Mining in West Sumbawa Regency, Indonesia

Baiq Dewi Krisnayanti (1), Ivano Vassura (2), Maywin Dwi Asmara (3), Ardiana Ekawanti (4), Herman Suheri (3), Elisabetta Zavoli (5,6)

(1) Department of Soil Science, Faculty of Agriculture, University of Mataram, Lombok, Indonesia
(2) Department of Industrial Chemistry "Toso Montanari", University of Bologna, Bologna, Italy
(3) Faculty of Agriculture, University of Mataram, Lombok, Indonesia
(4) Medical Faculty, University of Mataram, Lombok, Indonesia
(5) University of Bologna, Bologna, Italy
(6) Graffiti School of Photography, Rome, Italy

Corresponding author:
Krisnayanti, Baiq Dewi
Lombok, Indonesia
bdewi.krisnayanti@gmail.com

*Background*

The high value of gold reserves in West Sumbawa Regency and West Nusa Tenggara Province, Indonesia has resulted in an increase in small-scale gold mining activity in this area. Artisanal and small-scale gold mining is an attractive alternative livelihood for rural workers because it has good potential to improve the wealth of a community. Women and children are often involved in mining activities as miners usually bring their family to live close to their place of employment, thus women and children (<13 years old) are potentially highly exposed to mercury. The current study was conducted to better understand the life style, extent of mercury exposure, and the health of people living in the Taliwang artisanal and small-scale gold mining area, West Sumbawa Regency, West Nusa Tenggara Province, Indonesia. The present study was designed as a purposive field sampling study conducted in the Taliwang artisanal and small-scale gold mining area, West Sumbawa Regency, West Nusa Tenggara Province, Indonesia.

*Methods*

The subjects were miners and families including their children from three different areas within the West Sumbawa Regency: individuals directly exposed to mercury, indirectly exposed individuals and non-exposed individuals. Hair samples and medical examination were obtained from the subjects and subjects were questioned about their mercury exposure over time. Hair samples were collected at the time of the study. Ethical approval was granted by the Ethical Committee from Medical Faculty of University of Mataram, Indonesia.

*Results*
A sub-analysis of data showed that children in individuals directly exposed subgroups had highest mercury in their hair (mean 4.7 mg/kg) than indirectly exposed individuals subgroups (mean 1.5 mg/kg) and non-exposed individuals subgroups (mean 0.4 mg/kg). Medical examination on children showed no symptom of mercury intoxication yet. The results indicated that artisanal and small-scale gold mining activities were shown to affect the health of miners and their family in a very short time, as evidenced from the high mercury residue in their bodies above the normal level permitted by World Health Organization.

*Conclusions*
The current scale of artisanal and small-scale gold mining activity in the West Sumbawa Regency is predicted to rise, raising the possibility of environmental and health disasters.

---

### Environmental Pollution and Children's Health

**Philip J. Landrigan (1), Richard Fuller (2)**

**(1) Environmental Medicine and Public Health, Pediatrics, The Mount Sinai Hospital, New York, USA**
**(2) Pure Earth, New York, USA**

**Corresponding author:**
**Landrigan, Philip J.**
**New York, NY USA**
**philip.landrigan@mssm.edu**

*Background*
Pollution-related disease (PRD) is a massive and growing global problem. Diseases caused by pollution are responsible for nearly 9 million premature deaths each year, almost three times as many deaths as result from AIDS, tuberculosis and malaria combined. PRD, poverty and inequity are intertwined, and PRD falls most heavily upon children, women, and the poor. More than 90% of deaths due to PRD occur in low- and middle-income countries (LMICs).

The nature of pollution is changing. In rapidly developing countries, levels of ambient air pollution, toxic chemical pollution and soil pollution are increasing sharply as a consequence of urbanization, increasing motor vehicle use and the proliferation of toxic chemicals, pesticides and polluting industries. Asthma, neurodevelopmental disorders and sudden infant death syndrome are the main health consequences for children. In adults, health effects include chronic obstructive pulmonary disease, atherosclerosis, ischemic heart disease, stroke, renal disease, lung cancer and accelerated neurological degeneration. PRD is responsible for staggering economic costs. These costs include direct medical expenses as well as the diminished productivity of people who die prematurely or are disabled by PRD. These costs are largely invisible, but they are so great that they can distort health system spending and undermine the growth of entire societies.

Pollution can be prevented. Pollution and PRD are not the unavoidable consequences of development. Cost-effective pollution control strategies have been developed and successfully deployed in countries at all levels of income. These strategies are data-driven and are based on combinations of law, policy, civil engagement, governmental institutions and technology. They provide practical tools of proven efficacy that are available today to governments and people around the world. Interventions against PRD can win economic benefits as great as those associated with other highly effective public health programs such as childhood immunization.

PRD and pollution have been neglected in the global health and international development agendas. The resources allocated to pollution control and PRD prevention have, apart from water pollution and sanitation, been meager. Pollution has continued to worsen, and PRD has quietly become the world's largest cause of avoidable premature death.

### *Methods*

To address this great and growing global problem, we have launched the Global Commission on Pollution & Health (the Commission). The Commission is an initiative of The Lancet, the Global Alliance on Health and Pollution, and the Icahn School of Medicine at Mount Sinai. The Commission will present the first-ever analysis of the global burden of disease attributable to all types of pollution combined and the first analysis of PRD's global economic costs. It will present case studies of successful interventions against pollution.

### *Results*

The goal is to provide world leaders and global policy-makers the data and the strategic blueprint that they need to justify decisive action against pollution. The Commission's report will be published in early 2017.

### *Conclusions*

PRD is at a tipping point. While the problem is massive, it is now well mapped, and the tools for its control have been developed, tested and proven effective. With leadership and resources, pollution and PRD are problems than can be largely resolved within a generation.

### Exposure of Italian Children to Phthalates and Bisphenol A: Preliminary Results on Biomonitoring and Case-Control Studies from the LIFE PERSUADED Project

Cinzia La Rocca (1), Francesca Baldi (1), Barbara Baldini Ferroli (2), Giorgia Bottaro (2), Luca Busani (1), Emma Buzzigoli (4), Paola Cambiaso (2), Fabrizia Carli (4), Stefano Cianfarani (2,3), Demetrio Ciociaro (4), Raffaele Conte4, Annalisa Deodati (2), Enrica Fabbrizi (6), Melania Gaggini (4), Amalia Gastaldelli (4), Daniela Germani (3), Francesca Romana Mancini (1), Francesca Maranghi (1), Romana Marini (2), Laura Narciso (1), Anna Paola Pala (4), Andrea Raffaelli (4), Chiara Saponaro (4), Giuseppe Scirè (2,3), Gian Luigi Spadoni (2,3), Sabrina Tait (1), Roberta Tassinari (1), Giacomo Toffol (5)

(1) Istituto Superiore di Sanità, Rome, Italy
(2) Ospedale Pediatrico Bambino Gesù, Rome, Italy
(3) Università degli Studi di Roma Tor Vergata, Rome, Italy
(4) Consiglio Nazionale delle Ricerche, Pisa, Italy
(5) Associazione Culturale Pediatri, Italy
(6) Unità operativa Complessa Pediatria Ospedale Civile Augusto Murri, Fermo, Italy

Corresponding author:
La Rocca, Cinzia
Rome, Italy
cinzia.larocca@iss.it

*Background*

DEHP and BPA are non-persistent environmental contaminants recognized as endocrine disruptors, potentially affecting human health, and children in particular, because of their susceptible phase of development. The Phthalates and Bisphenol A Biomonitoring in Italian Mother-Child Pairs: Link Between Exposure and Juvenile Diseases (LIFE PERSUADED) Project is focused on the evaluation of the children exposure to plasticizers, such as Diethylhexyl phthalate (DEHP) and Bisphenol A (BPA), through the biomonitoring study. Moreover, the potential association with idiopathic premature thelarche and precocious puberty and idiopathic obesity is evaluated through case-control studies.

*Methods*

This project has been approved by the Ethics Committees of Istituto Superiore di Sanità and Bambino Gesù Children's Hospital.

In HBM, 2160 mother-child pairs from urban and rural areas of the Italian geographical macro-areas (North, Centre, South) are recruited by trained family pediatricians. Data on lifestyles, food storage and consumption are collected through a structured questionnaire and a food diary. BPA and DEHP metabolites (mono-(2-ethylhexyl) phthalate (MEHP), 5OH-MEHP and 5oxo-MEHP) levels are determined in urine samples by electrospray ionization, liquid chromatography–mass spectrometry.

30 girls for each group, aged 2-7 years (idiopathic premature thelarche and precocious puberty) and 30

boys and 30 girls, aged 6-10 years (idiopathic obesity) are recruited. BPA and DEHP metabolite levels are assessed in urine samples, and clinical and toxicological biomarkers related to puberty onset and obesity in serum samples.

### *Results*

The project approach and descriptive preliminary results on DEHP metabolites and BPA internal levels will be presented at the meeting.

### *Conclusions*

LIFE PERSUADED fills the data gap on DEHP and BPA exposure in Italian children by setting background levels for susceptible groups of population and implementing the EU database. Data integration for endocrine disruptors risk assessment, the evaluation of determinants of exposure as well as the creation of a biobank for urine samples will represent relevant output of the project.

---

### Low Level Arsenic Exposure During Pregnancy in the 3xG Cohort In Flanders: Less Efficient Detoxification is Associated with Higher Oxidative Stress

**Nathalie Lambrechts (1), Eva Govarts (1), Ann Colles (1), Sylvie Remy (2), Bert Morrens (3), Martine Leermaekers (4), Els Van de Mieroop (5), Elly Den Hond (5), Vera Nelen (5), Ilse Loots (3), Greet Schoeters (1,2,6)**

(1) Environmental Health and Risk, Flemish Institute for Technological Research, Mol, Belgium
(2) Department of Biomedical Sciences, University of Antwerp, Antwerpen, Belgium
(3) Department of Political and Social Sciences, University of Antwerp, Antwerpen, Belgium
(4) Department of Analytical and Environmental Chemistry, Free University of Brussels, Belgium
(5) Department of Health, Provincial Institute for Hygiene, Antwerp, Belgium
(6) University of Southern Denmark, Department of Environmental Medicine, Odense, Denmark

Corresponding author:
Lambrechts, Nathalie
Mol, Belgium
nathalie.lambrechts@vito.be

### *Background*

Increasing epidemiologic evidence indicates that even low level arsenic exposure affects adverse pregnancy outcomes. On the other hand, it is known that arsenic metabolism changes during pregnancy by means of detoxification. The adverse outcome pathways from arsenic exposure during pregnancy need further clarification.

### *Methods*

This study investigates whether urinary arsenic species and arsenic methylation efficiency are associated with oxidative stress in 151 pregnant women of the 3xG cohort in Flanders, Belgium (2012-2015). Urine samples were collected between 25 and 35 weeks of gestation and different arsenic species (inorganic arsenic (inorganic arsenic; arsenic (III), arsenic (V)), monomethyl arsenic acid (MMA) and dimethyl arsenic acid) were measured using liquid chromatography–mass spectrometry. Multiple linear regression analyses were performed to evaluate the associations between arsenic exposure and oxidative stress, measured by the amount of 8-hydroxy-2′-deoxyguanosine (8-OHdG, ln-transformed) in the same urine samples (enzyme-linked immunosorbent assay). The associations were adjusted for age and smoking before sample collection. Ethical approval was granted by the Ethical Committee of Antwerp University (Hospital) and of the local hospitals.

### *Results*

Increased 8-OHdG levels values were associated with higher MMA levels: for each µg/l increase in MMA, 8-OHdG increased with 24% (µg/l) (95% confidence interval =0.5-51.7%). Furthermore, a decreasing trend between the secondary methylation index (=dimethyl arsenic acid/MMA) and 8-OHdG was found (β=0,998 (95% confidence interval =0.996-1.000)). No significant associations were observed for inorganic arsenic, dimethyl arsenic acid and the primary methylation index (=MMA/ inorganic arsenic) with 8-OHdG.

### *Conclusions*

The information in this study indicates that in a region with a relatively low environmental arsenic burden, the toxic metabolite MMA is associated with increased oxidative stress in pregnant women. Furthermore, mothers with less efficient detoxification of arsenic had higher levels of 8-OHdG.

The 3xG study is part of the cAT project, funded by NIRAS and the local partnerships MONA and STORA.

---

### Lead and Mercury Exposure Associated with Low Resting Heart Rate in Community Children

**Jianghong Liu, Naixue Cui**

**School of Nursing, University of Pennsylvania, Pennsylvania, USA**

**Corresponding author:**
**Liu, Jianghong**
**Philadelphia, Pennsylvania, USA**
**jhliu@nursing.upenn.edu**

### *Background*

Few studies have examined the neurotoxic influence of heavy metals on the autonomic nervous system. Previously, one study showed that occupational chronic lead exposure decreased resting heart rate (RHR). The purpose of our study is to test whether environmental lead and mercury exposure is associated with decreased RHR in community children.

### Methods

As part of the China Jintan Cohort Study, 564 children aged 12 years (standard deviation=0.6) were tested for blood levels of lead (BLL) and mercury (BML) and RHR. RHR was recorded using electrocardiography placed axially on the left and right ribs. Generalized linear models (GLMs) were conducted to test the relationship between BLL and BML and the RHR controlling for children's sex, age, and socioeconomic status. Analysis was clustered at the preschool level when the children were recruited to adjust for standard error. Ethical approval was granted by the University of Pennsylvania Institutional Review Board.

### Results

The mean (standard deviation) BLL and BML was 3.1 (1.2) µg/dL, with a median value of 2.9 µg/dL and 0.1 (0.07) µg/dL and a median value of 0.1, respectively. GLMs results showed that after adjusting confounders, children with BLL (B=-1.23, p=0.010) or BML (B=-1.86, p=0.013) higher than the median level showed decreased RHR. Particularly, compared with girls, boys were more likely to have decreased RHR when their BML exceeded the median level (i.e. 0.1 ug/dL) (B=-3.33, p<0.001). No gender differences were observed for BLL and RHR. A further analysis showed that RHR was the lowest among children with both high BLL and BML (B= -3.30, p<0.001), and this combined effect was greater among boys (B=-4.09, p<0.001) than among girls (b=-2.64, p=0.147).

### Conclusions

Low RHR is an indication of chronic under-arousal and has been implicated to psychopathology, particularly for externalizing behavior. We previously reported that lead exposure was associated with increased behavioral problems in community children. Our finding of the relationship between heavy metal exposure and low RHR provides the direction for our future study testing the possible mediating role of low RHR on the relationship between heavy metals and behavioral problems in children.

### The Role of Perinatal Essential Fatty Acids In Attention Deficit and Hyperactivity Disorder Symptoms and Cognitive Functions

Mónica López-Vicente (1,2,3,4), Núria Ribas Fitó (5), Sílvia Alemany (1,2,3,4), Mario Murcia (4,6), Marisa Rebagliato (4,6), Jesús Ibarluzea (7,8), Aitana Lertxundi (7,8), Ana Fernández-Somoano (4,9), Adonina Tardón (4,10), Martine Vrijheid (1,2,3,4), Jordi Sunyer (1,2,3,4), Jordi Júlvez (1,2,3,4)

(1) Barcelona Institute of Global Health (ISGlobal), Barcelona, Spain
(2) Municipal Institute of Medical Research (IMIM-Hospital del Mar) Barcelona, Spain
(3) Department of Experimental and Health Sciences, Pompeu Fabra University (UPF), Barcelona, Spain
(4) CIBER Epidemiologia y Salud Publica (CIBERESP), Spain
(5) Fundació Orienta, CSMIJ Gavà, Spain
(6) FISABIO-Universitat de València-Universitat Jaume I Joint Research Unit of Epidemiology and Environmental Health, Valencia, Spain
(7) Department of Medicine, EHU/UPV, Donostia-San Sebastián, Spain
(8) Health Research Institute, BIODONOSTIA, Donostia-San Sebastián, Spain
(9) Preventive Medicine and Public Health, University of Oviedo, Asturias, Spain
(10) IUOPA, University of Oviedo, Asturias, Spain

Corresponding author:
López-Vicente, Mónica
Barcelona, Spain
monica.lopez.vicente@gmail.com

*Background*

Essential fatty acids play a key role in brain development. The balance between omega-6 and omega-3 is important for an optimal nutrition pattern. We aim to assess the role of omega-6/omega-3 ratio on child attention deficit and hyperactivity disorder (ADHD) symptoms and cognitive functions.

*Methods*

This study was based on the population-based birth cohort INMA, including 4 different Spanish regions (Asturias, Gipuzkoa, Sabadell and Valencia). Fatty acids were collected from whole cord blood samples by using venipuncture of cord vessels before the placenta was delivered. The omega-6/omega-3 ratio was calculated by dividing arachidonic acid (AA) with the sum of docosahexaenoic acid (DHA) and eicosapentaenoic acid (EPA). The ADHD symptoms were reported by each child's teacher using the Diagnostic Manual of Mental Disorders, 4th ed. (ADHD DSM-IV) at age 4. The child cognitive functions of the children were assessed by a psychologist using McCarthy Scales of Children's Abilities (MSCA) at age 4. Pooled negative binomial regression models were used for ADHD symptoms (n=571). Pooled linear regression models were used for MSCA (mean=100, SD=15) (n=670). All models were adjusted for child gestational age, birth weight, gender, age during testing, maternal social class, smoking during pregnancy and cohort location. The study was approved by the ethics committees of participating hospitals in Valencia and Sabadell (Spain).

*Results*

Omega-6/omega-3 ratio was associated with ADHD symptoms (IRR=1.23; 95%CI=0.98, 1.53) at 4 years old. Adverse associations were observed between omega-6/omega-3 ratio and different cognitive domains assessed with MSCA; general cognition (coef.: -1.34; 95%CI=-2.60, -0.09), verbal (coef.: -1.31; 95%CI=-2.63, 0.002), memory (coef.: -1.67; 95%CI=-3.06, -0.29), and executive function (coef.: -1.32; 95%CI=-2.60, -0.05).

*Conclusions*

Higher omega-6/omega-3 ratio on cord blood was related to more ADHD symptoms and worse cognitive functioning at 4 years of age. The identification of nutritional biomarkers that explain its effects on child cognitive development will enable the implementation of appropriate and effective nutritional recommendations as well as interventions during pregnancy.

### Prenatal Exposure to Outdoor Airborne Trace Elements and Cognitive and Psychomotor Development During Childhood in Four European Birth Cohorts

**Malgorzata Joanna Lubczynska**

**Barcelona Institute for Global Health, Centre for Research in Environmental Epidemiology, Barcelona, Spain**

**Corresponding author:**
**Lubczynska, Malgorzata Joanna**
**Barcelona, Spain**
**gosia.lubczynska@isglobal.org**

*Background*

Studies on the association between exposure to elemental composition of particulate matter in outdoor air and neuropsychological development are sparse. We aimed to investigate the association of prenatal exposure to trace elements of outdoor particulate matter with diameter of <2.5 μm ($PM_{2.5}$) with cognitive and psychomotor development during childhood.

*Methods*

We analyzed data from 4 European population-based birth cohorts – Generation R (The Netherlands), Duisburg (Germany), GASPII (Italy) and INMA-Sabadell (Spain) – with mother–infant pairs recruited between 2000 and 2006. Monitoring campaigns measuring $PM_{2.5}$ levels were performed between 2008 and 2011 in all corresponding regions and the elemental composition of the particles was determined. Individual exposures to 8 elements (copper, iron, potassium, nickel, sulfur, silicon, vanadium and zinc) that represent major anthropogenic sources of air pollution were estimated at birth addresses using land use regression models. Cognitive (general, verbal, and non-verbal) and psychomotor (global, fine, and gross) development was assessed between ages 1 and 9. Adjusted region-specific effect estimates were combined using random-effects meta-analysis. Ethical approval was granted by local authorized institutional review boards.

### Results

There were 2904 children included in this complete case analysis. That is, children that had information on exposure, at least one of the outcomes and all the selected potential confounding variables. Prenatal exposure to vanadium and nickel was associated with a reduced verbal development (-1.71 points [95% CI = -3.06 to -0.37] per increase of 2 $\mu g/m^3$ of vanadium and -1.96 points [95% CI = -3.46 to -0.46 per increase of 1 $\mu g/m^3$ of nickel]). A similar tendency, although statistically not significant, was found for exposures to sulfur and silicon with verbal development; for exposure to nickel, sulfur, and silicon with general cognitive development; and for exposure to all elements with fine psychomotor development. In a few cases, exposure to some elements showed a tendency towards improved non-verbal and gross psychomotor development, although these results were statistically non-significant as well.

### Conclusions

Prenatal exposure to airborne trace elements vanadium and nickel of outdoor $PM_{2.5}$ was associated with an impaired verbal development during childhood.

---

### Promoting Health: Recommendations of Food Consumption as a Tool to Minimize Exposure of Pollutants in Children

Rosa Maria Lull Cantallops

Food Safety Unit, Balearic Islands Government, Balearic Islands, Spain

Corresponding author:
Lull Cantallops, Rosa Maria
Balearic Islands, Spain
rmllull@dgsanita.caib.es
cantallull@gmail.com

### Background

Health care workers (HCW) are opinion leaders in the field of food safety and their promotion in the population is very effective in public health. Food is an important exposure source for many environmental contaminants and children are the most vulnerable age group for most of them.

### Methods

With the aim to improve the knowledge about food contaminants for HCW, 275 HCW in 16 of the main hospitals from the Balearic Islands attended 2 hours of lessons in 2015. A test before and after the lesson was given to assess the knowledge of HCW about how to prevent the exposure of children and pregnant women to food contaminants and to determine their opinion about the course. In addition, teaching materials for families was provided to the HCW.

### Results

Of those workers, 60% answered the test correctly. The average percentage of correct responses prior the training was 10%. The most valued aspects were the high profit and the opportunity to share scientific knowledge about risks in food between HCW and public health workers. Their demands have been to extend the course contents and its duration.

### *Conclusions*

The low percentage of tests performed was due to the difficulty of workers leaving their tasks to arrive on time at the sessions. This fact, added to their wish to receive more extended content and longer courses, requires another course format. Also, the low score of correct answers shows the necessity to provide more courses to HCW.

In 2016, a new online food safety course for HCW will be available in the Balearic Islands. The attendees will connect when they can and will have access to more extended content. To access the content they will have to take the introductory tests, and to obtain the certificate they will have to do the post test, and thus the entirety of the data will be collected. We will dtermine if better knowledge is achieved with online lessons in order to better prevent exposure of children to contaminants.

---

### Mercury Concentrations in Commercial Seafood from the Western Mediterranean Sea

Rosa Maria Lull Cantallops (1), Rafael Coll (1), Manuel Perez (1), Carlos Llopis (1), Irene García (1), Teresa Rey-Maquieira(2), Cristina Vidal (2), Eva Junque (3), Anna Arce (3), Joan O. Grimalt (3)

(1) Food Safety Unit, Balearic Islands Government, Balearic Islands, Spain
(2) Public Health Laboratories Unit, Balearic Islands Government, Balearic Islands, Spain
(3) Institute of Environmental Assessment and Water Research, Spanish National Research Council, Barcelona, Spain

Corresponding author:
Lull Cantallops, Rosa Maria
Balearic Islands, Spain
rmllull@dgsanita.caib.es

### *Background*

Mercury is a strong neurotoxic agent that can cause severe neurological damage to children because of their immature metabolism and organs. Mercury (Hg) emissions in the atmosphere due to mining and fossil fuel burning ($Hg^0$) are deposited into seawaters after oxidation to $Hg^{2+}$. There, Hg may undergo bioaccumulation and scavenging by organic-rich particles, be eventually transported from surface to deep waters and reduced back to $Hg^0$ and to $CH^3Hg$.

Fish and shellfish consumption is the known predominant input of this metal into human populations of Mediterranean regions. Attention has been focussed on oily fish as the main group of species responsible for the incorporation of Hg into the human diet. Conversely, few studies on the potential role of lean fish regularly consumed by humans have been reported so far in the scientific literature. The present study is therefore aimed to determine the Hg levels in commercial seafood from the Mediterranean Sea.

### *Methods*

Samples were caught between February 2014 and December 2015 in the Balearic Islands. The sampling plan included the most consumed and fished species in the Balearic Islands, grouping them for their study in three trophic levels with the aim to identify the species which carry the most Hg. Samples were collected in local fish markets and auction halls with validated sampling and analysis methods. Information gathering included data for each specimen of variables related with the amount of mercury in fish, as size, weight and catch location.

### *Results*

Eighty-two percent of the samples showed detectable Hg levels. The levels were generally higher than those reported in seafood from the Atlantic Ocean. A substantial number of samples exceeded the maximum level set forth by the European Union Maximum Limits of 0.5 and 1.0 mg/kg wet weight. Twenty-nine percent of the thirty-nine fish and shellfish species studied had average concentrations above these limits.

### *Conclusions*

The present study shows that not only oily fish, but also lean and shellfish species from the Mediterranean Sea have mercury concentrations above the recommended European Union Maximum limits. A reassessment of the impact of fish consumption from this region on children and the general population is needed. This assessment should estimate how much mercury intake can be attributed to this source and along with its health effects.

---

### Lung Function Growth Trajectories in Children in Relation to Prenatal Exposure to Particulate Matter — Cracow Cohort Study

Renata Majewska (1), Agnieszka Pac (1), Elǒbieta Mroz (1), Frederica P. Perera (2)

(1) Epidemiology and Preventive Medicine, Jagiellonian University Medical College, Krakow, Poland
(2) Columbia Center for Children's Environmental Health, Columbia University, New York, USA

Corresponding author:
Majewska, Renata
Krakow, Poland
renata.majewska@uj.edu.pl

### *Background*

Patterns of early life function development during childhood may be helpful in understanding the pathogenesis of respiratory diseases throughout life. The aim was to investigate the individual growth trajectories of children's lung functions as they get older by gender and prenatal personal level of exposure to airborne particulate matter 2.5 ($PM_{2.5}$), which was hypnotized to decline studied parameters. Both the shape of trajectories and the degree of variability in growth among children were of interest.

*Methods*

The study sample was comprised of 322 children from a Cracow birth cohort who annually underwent standard lung function testing (forced volume capacity, forced expiratory volume in 1 s and forced expiratory flow 25–75%) between 4 and 9 years of age. Personal measurements of $PM_{2.5}$ were performed over 48 hours in the second trimester of pregnancy among pregnant women recruited prenatally to the study. Data on child development and health status as well as exposure assessment were collected regularly based on standardized questionnaire. Growth trajectories of children's lung functions were adjusted by polynomial multilevel linear mixed models. The parents of all children involved in the study gave their informed consent and the Ethical Committee of the Jagiellonian University in Krakow and Columbia Presbyterian Medical Center approved the research.

*Results*

Lung function trajectories differed significantly for boys and girls for FVC and FEV1. Boys had a higher increase than girls (mean annual difference: 23.1ml (95% confidence interval: 8.8;37.1), p=0.002 and 22.6 ml (95% confidence interval: 10.0;35.2), p<0.001 ), respectively). In cases of FEF25-75%, trajectories had similar shape for boys and girls. Significant lung function impairment (decrease in FVC and FEV1) was observed from 4 through 9 years among subjects prenatally exposed to higher levels of $PM_{2.5}$, but not in the case of FEF25-75%.

*Conclusions*

High levels of prenatal exposure to $PM_{2.5}$ might be related to diminishing lung function growth in children.

---

### Prenatal Exposure to Perfluoroalkyl Substances and Cardio-Metabolic Risk Components During Early Childhood

**Cyntia B. Manzano-Salgado**

**Barcelona Institute for Global Health, Centre for Research in Environmental Epidemiology, Barcelona, Spain**

**Corresponding author:**
**Manzano-Salgado, Cyntia B.**
**Barcelona, Spain**
**cyntia.manzano@isglobal.org**

*Background*

Prenatal exposure to perfluoralkyl substances (PFAS) might increase obesity and cardio-metabolic (CM) risk during childhood, but results are inconclusive. We evaluated the effect of PFAS on individual and combined CM risk components during childhood in a Spanish birth cohort.

*Methods*

We measured perfluorohexanesulfonic acid (PFHxS), perfluorooctanesulfonic acid (PFOS), perfluorooctanoic acid (PFOA), and perfluorononanoic acid (PFNA) in maternal plasma. We assessed rapid growth at 6 months of life (n=1109) and age-and sex-specific z-scores for body mass index, waist circumference and mean arterial pressure at 4 (n=1,230) and 7 (n=1094) years. Using non-fasting blood samples at 4 years we measured lipid concentrations (n=629): total cholesterol, triglycerides and, high-density and low-density lipoprotein cholesterol. We calculated a continuous CM risk score at 4 years; a higher score indicated a higher CM risk. All participating women signed written informed consent before beginning the study. The study was approved by the regional ethical committees of each cohort.

### *Results*

Twenty-four percent of children were rapid growers at 6 months and 30% and 35% were overweight at ages 4 and 7, respectively. At 6 months, PFOA was associated with increased weight gain in boys [β=0.22 (95% confidence interval: 0.04, 0.40)] but not in girls [β=-0.06 (-0.21, 0.10)] (p-value for sex-interaction=0.08). We found no association between PFAS and body mass index z-scores, overweight or waist circumference at 4 and 7 years. PFAS tended to decrease mean arterial pressure z-scores at 4 years of age, but was not statistically significant. Higher PFAS concentrations increased total cholesterol and triglycerides at 4 years, especially PFHxS [β=6.32 (0.96, 11.68)]. Higher PFOA concentrations increased the CM risk score at age 4, especially in boys [β=1.37 (0.03, 2.71)].

### *Conclusions*

This is one of the largest studies to comprehensively assess the effect of PFAS on individual and combined CM risk components during early childhood. Our results suggest that PFAS can affect some CM risk components during childhood, especially in boys. Further replication of our results is needed.

---

### Mobile Phone and Other Digital Technology Use in UK Adolescents: The SCAMP Cohort

**Mireille B. Toledano, Michael O. Mireku, Charlotte Fleming, Irene Chang, William Mueller, Iroise Dumontheil, Michael Thomas, Martin Röösli, Paul Elliott**

**Imperial College London, London, UK**

**Corresponding author:**
**Mireku, Michael Osei**
**London, UK**
**m.mireku@imperial.ac.uk**

### *Background*

Scientists remain uncertain as to whether children's developing brains are vulnerable to radiofrequency (RF) waves emitted from mobile phones and other wireless devices. The Study of Cognition, Adolescents and Mobile Phones (SCAMP) is a three-year prospective cohort study of adolescents across London, UK, which aims to investigate whether children's use of mobile phones and other wireless devices influences their neurocognitive/behavioural development.

### Methods
Data on cognitive function, wireless device use and lifestyle are collected at baseline and follow-up via school-based computerised assessments and optional parent/pupil home-based questionnaires. Parents are invited to consent to linkage of their child's assessment data with routine records (e.g. health and educational records, mobile traffic data), thus allowing for comparisons between self-reported and objective mobile phone use. Biological samples (e.g. urine, saliva) are collected to provide additional information about potential confounders, such as pubertal status. A RF personal monitoring study is also being conducted in a subset of the cohort to gain an in-depth understanding of children's personal RF exposure and the relative contribution of each RF source. Ethical approval was granted by the North West Haydock Research Ethics Committee.

### Results
To date, 5,504 predominantly 11 to12-year-olds participate in SCAMP (53.2% female, mean age 12.0 (SD 0.4); 42.3% White, 20.3% South Asian, 15.9% Black, 11.5% Mixed, 9.9% Other). Preliminary analysis shows that over 80% of participants own a mobile phone. Device usage is comparable among boys and girls, apart from gaming. Black adolescents consistently report highest use across all devices. Validating self-reported information against traffic data in a subset revealed children had underestimated call frequency, but overestimated call duration and text messages.

### Conclusions
SCAMP will improve our understanding of children's RF exposures, device use, and cognitive function and will provide an evidence base to inform public health policy in this area.

---

### Current Findings on Maternal Gut Microbiome and Polychlorinated Biphenyls Concentration in Birth Cohort Study With Omics Analysis: Chiba Study of Mother and Children's Health

Chisato Mori, Kenichi Sakurai, Akifumi Eguch, Kaori Tachibana, Emiko Todaka

Center for Preventive Medical Sciences, Chiba University, Chiba, Japan

Corresponding author:
Mori, Chisato
Chiba, Japan
cmori@faculty.chiba-u.jp

### Background
In previous studies, we have reported that environmental contaminants such as polychlorinated biphenyls (PCBs) were detected in maternal blood, umbilical cord and cord blood. On the other hand, in animal studies, it has been reported that intake of PCBs induced the alteration of the microbiome. Moreover, several studies have reported that the gut microbiome influences the balance between health and disease. A birth cohort study, the Chiba Study of Mother and Children's Health focuses on environmental health effects on foetuses, and involves multiomics analysis and has been conducted since 2014. Currently, the correlation between maternal blood PCB levels and microbiome status is being investigated. The associations between changes in the maternal gut microbiome and alteration of differentially methylated regions of diabetes-associated genes in umbilical cord samples are also examined.

### *Methods*

The Chiba Study of Mother and Children's Health consists of three hospital-based cohorts. Ethical approval was given by the Biomedical Research Ethics Committee of the Graduate School of Medicine, Chiba University. Consent to participate was obtained from 433 pregnant women. Among those, 376 women answered the questionnaires regarding their characteristics and life style during the early gestational period. The mean age was 32.5 (± 4.4) years old. Blood PCBs concentration level in 93 women was measured by gas chromatography–mass spectrometry. The microbiome of 10 participants was studied. The 16S rRNA metagenome analysis of maternal stool samples and deoxyribonucleic acid methylation assays of umbilical cord samples were performed.

### *Results*

The mean concentration of blood total PCBs was 420 pg g−1 wet wt, in which chlorinated biphenyl (CB)153 (mean: 100 pg g−1 wet wt), CB138 (mean: 70 pg g−1 wet wt), and CB180 (mean: 58 pg g−1 wet wt), contributing the most of the total PCB. In our preliminary analysis, total PCB level and one genus of gut microbiome showed positive correlation. Additionally, the proportion of *Firmicutes* in the maternal gut were associated with deoxyribonucleic acid methylation levels of some diabetes-associated genes in umbilical cord samples.

### *Conclusions*

These findings suggest that PCB exposure of mothers might affect the microbiota components of mothers and epigenetic change in fetuses. Further detailed studies are needed to reveal whether maternal exposure will affect maternal microbiota components or epigenetic statues in fetuses.

---

### Poor Education – Poor Jobs – Poor Health

**Hanns Moshammer, Wali Abdul Khan**

**Institute for Environmental Health, Medical University of Vienna, Vienna, Austria**

**Corresponding author:**
**Moshammer, Hanns**
**Vienna, Austria**
**hanns.moshammer@meduniwien.ac.at**

### *Background*

Lack of schooling during childhood leads to a vicious circle with access only to hazardous, insecure, and poorly paid jobs, with higher health risks and poorer access to health care, successively with more disease and injuries, again less income and consecutively poorer housing and even more risks. Not only does this lead to individual misfortune, but also affects the fate of offspring. The mechanisms are even more visible in developing countries without a well-established welfare-system with social securities and free access to public education.

*Methods*

We investigated health in relation to occupational exposures in the cotton industry of Pakistan. A random sample of plants in the area of Faisalabad was selected, stratified by plant size. Most workers present at the plant at the day of visit received and answered a questionnaire about job history and health complaints. A random sample of workers in each plant was further examined by spirometry and buccal cell test. Dust measures were performed during each visit. Ethical approval was granted by the Ethical Review Committee of the Health Services Academy, Ministry of National Health Services, Regulation and Coordination in Islamabad, Pakistan.

*Results*

We found that small enterprises producing for the local market generally, but also on demand for big companies with long-standing contracts with European and American traders, not only displayed the highest dust exposures, poorest occupational safety measures and hygiene, but also had the longest shifts (typically 2 shifts of 12 hours each), and the least educated work-force, typically with a working history from the age of 15. These workers also fared poorest on several health parameters, from poorer lung function to a long list of reported symptoms not exclusively but also including respiratory symptoms, along with signs of cytotoxicity and genetic damage.

*Conclusions*

In this observational study it was not possible to disentangle the effects of longer years of exposure because of early initiation of work, longer occupational exposure per day, and higher exposures due to poorer occupational hygiene. Nevertheless we want to point out that all these factors were primarily caused by poor education and early termination or even lack of proper schooling.

---

### An Unexpected Epidemic of Respiratory Distress

Hanns Moshammer

Institute for Environmental Health, Medical University of Vienna, Vienna, Austria

Corresponding author:
Moshammer, Hanns
Vienna, Austria
hanns.moshammer@meduniwien.ac.at

*Background*

In 2011, a series of severe cases of respiratory distress came to the notice of Korean officials. The index case was a young female who developed deadly bronchiolitis obliterans a few weeks after an uncomplicated pregnancy. Consecutive examinations revealed that dozens of similar cases had appeared throughout the country, mostly in children and in females, the latter often either pregnant or shortly after pregnancy. The mortality rate was exceptionally high among cases and many more cases only survived after lung transplantation. We will summarize the findings of other authors reported in the literature. The aim is to draw attention to a highly instructive episode.

*Methods*

As infectious causes could not be established, further studies focused on toxic exposures. Because of the characteristics of the affected persons (no common occupational exposure), indoor pollutants were suspected.

*Results*

It turned out that cases had used humidifiers at home and had treated these with disinfectants as advertised by industry. Several case-control studies clearly indicated the causal role of these disinfectants. Animal studies demonstrated the same severe damage of lung tissue after inhalative exposure towards a disinfectant previously deemed “safe” and “not toxic”. Further studies also revealed that similar epidemics had happened for the last couple of years, usually in the cold season, without anyone taking notice.

*Conclusions*

General implications for toxicity testing and for the application of the precautionary principle will be discussed.

### Hazards and Risks in the Glyphosate Debate

**Karl Ernst von Mühlendahl, Matthias Otto**
**Kinderumwelt gGmbH der Deutschen Akademie für Kinder- und Jugendmedizin, Osnabrück, Germany**

**Corresponding author:**
**Mühlendahl, Karl Ernst von**
**Osnabrück, Germany**
**muehlend@uminfo.de**

*Background*

The question of whether licensing of glyphosate, a widely used herbicide, should be continued or phased out has been subject to intense and sometimes very emotional controversy during the last two years. The media, non-governmental organizations (NGOs), farmers, scientists, and official and government agencies, e.g. the International Agency on Research of Cancer (IARC), the Joint Meeting of the FAO/WHO on Pesticide Residues (JMPR), the German Federal Institute for Risk Assessment (Bundesinstitut für Risikobewertung (BfR)), and the European Food Safety Agency (EFSA) have differed in their opinions, namely on the carcinogenicity of glyphosate. Despite its low acute toxicity and the fact that glyphosate is not persistent, the worldwide distribution of some 700,000 tons per year, resulting in the practically ubiquitous presence of this substance, doubtlessly warrant thorough evaluation.

*Methods*

The publications and arguments of different agencies and NGOs have been reviewed and evaluated, including, in some cases, the underlying primary studies and publications. Since the carcinogenicity of glyphosate has been the aspect most widely and intensely discussed, our analysis centered on this question, but we are aware that endocrine disruption and mutagenicity are further aspects of interest.

*Results*

Both BfR and EFSA see no risk for human health under the present conditions of application. However, during their deliberations, the IARC classified glyphosate as probably carcinogenic to humans (Group 2A), arguing that, although “there is limited evidence in humans for the carcinogenicity of glyphosate”, there is an “observed positive association for non-Hodgkin lymphoma. There is sufficient evidence in experimental animals for carcinogenicity”. The animal experiments cited by the IARC report marginal effects with borderline significances in some groups under chronic and very high glyphosate dosage. The JMPR declared that there was no risk for humans, considering real exposure rates.

*Conclusions*

The unresolved conflicts within these official agencies and with NGOs are caused by insufficient wording and lack of public awareness of the fact that hazard and risk denote different categories. Several years ago, the IARC defined these concepts: “A cancer ‘hazard’ is an agent that is capable of causing cancer under some circumstances, while a cancer ‘risk’ is an estimate of the carcinogenic effects expected from exposure to a cancer hazard”, being “one part of the body of information on which public health decisions may be based”. The European Commission prolonged the license for another 18 months on June 30, 2016, thus giving time for more deliberation and discussion.

---

### Autism Spectrum Diseases, Including Asperger Syndrome: Fairy Tales and Facts about Environment and Genetics

**Karl Ernst von Mühlendahl**

**Kinderumwelt gGmbH der Deutschen Akademie für Kinder- und Jugendmedizin, Osnabrück, Germany**

**Corresponding author:**
**Mühlendahl, Karl Ernst von**
**Osnabrück, Germany**
**muehlend@uminfo.de**

*Background*

During the last two or three decades, awareness of the existence of autism and, more generally, autism spectrum disorders (ASD), also classified as pervasive developmental disorders (PDD), has increased. This has led to a wider recognition and more frequent diagnosis of ASD. It has been debated, but remains questionable, whether the incidence really has increased. Both genetic and environmental factors are important for the phenotypic manifestation of ASD.

*Methods*

The more recent literature on the genetics of ASD and on precipitating environmental factors have been evaluated and reviewed.

*Results*

The prevalence of ASD is generally reported as 5 to 6 per thousand population, and in some studies and populations even more than 10 per thousand population. The male to female ratio is about 3:1. Homozygotic twins very frequently both manifest the ASD phenotype, more than 95% in boys, and around 80% in girls. The recurrence rate in families with more than one affected member is about 50% for boys and 20% for girls. Thus heritability apparently is one cause, and is probably necessary to make individuals susceptible to environmental factors. The extensive literature on environmental conditions is partly highly speculative, in other publications, however, more solidly founded. For instance, five epidemiologic studies in the United States show statistically significant associations between air pollution or residence close to highways and ASD incidence.

*Conclusions*

As the genesis of ASD is multifactorial, there remains much to be elucidated as to the causes and mechanisms which can lead to its phenotypic manifestation. However, there exist already sufficient data showing that adequate nutrition during pregnancy is important, and, if necessary, adequate supplementation of vitamins, folate and micronutrients can decrease the risk of ASD in offspring. Avoidance of other environmental conditions, as heavy traffic and pesticide exposure, or fever during pregnancy, is difficult for most families.

---

### Autistic Symptoms Associated with Childhood Lead Poisoning

**Wan Nedra (1), Insan Satria (2), Sabriyani Permatasar (2)**

**(1) Department of Pediatric, Faculty of Medicine, YARSI University, Jakarta, Indonesia**
**(2) YARSI University, Jakarta, Indonesia**

**Corresponding author:**
**Nedra, Wan**
**Jakarta, Indonesia**
**wan.nedra@yarsi.ac.id**

*Background*

Autism is known as a complex developmental disability. Experts believe that autism presents itself during the first three years of a person's life. The condition is the result of a neurological disorder that has an effect on normal brain function, affecting development of the person's communication and social interaction skills. Lead is a neurotoxin to which the developing brain is particularly vulnerable. Moreover, lead poisoning in children is known to negatively affect brain systems implicated in cognitive, communication, and social functioning. One potential weakness of autistic children is that they are less able to remove heavy metals from the body so that the metal accumulates in the body and brain.

*Methods*

This observational study with a cross-sectional approach, examined 44 children diagnosed with autism n Grogol Jakarta and the Islamic Hospital Mental Klender Jakarta. Data were collected through interviews, and blood lead levels were checked by flame atomic emission absorption spectrophotometer. Ethical approval was granted by YARSI University.

*Results*

Hair examination of children with autism showed elevated levels of heavy metals. Examination of the 44 children with autism, ages 1-10 years old, 97% boys and 23% girls, showed that 16.7% had high levels of heavy metals. Patients were diagnosed since one-month-old.

*Conclusions*

If a child is showing symptoms of autism, then detoxification and other types of therapies, such as speech therapy, behavioral, occupational must be done. If there has been damage to the brain, detoxification cannot directly fix the symptoms. Detoxification is just to prevent further damage. Toxic metals are not the cause of autism, but can be a trigger, or aggravate the symptoms of autism.

---

### Relationships Between the Exposure of Factory Air Pollution with Asthma Impacted in Children Who Live in Shelters in an Industrial Area in Jakarta, Indonesia

Wan Nedra (1), Qomariah Ulfahimayati (2), Siti Maulidya Sari (3), Gea Putri Pertiwi (3)

(1) Department of Pediatrics, Faculty of Medicine, YARSI University, Jakarta, Indonesia
(2) Depertment of Physiology, Faculty of Medicine, YARSI University, Jakarta, Indonesia
(3) Faculty of Medicine, YARSI University, Jakarta, Indonesia

Corresponding author:
Nedra, Wan
Jakarta, Indonesia
wan.nedra@yarsi.ac.id
nedrakomaruddin@yahoo.com

*Background*

Exposure to hazardous environments can cause damage to the respiratory tract, particularly in individuals who have atopy who may suffer from continuous inflammation and can also cause asthma in children. A recent study describes the correlation between exposures to traffic air pollution with the respiratory tract exchanges. One evidence of this correlation is the increasing concentration of nitric oxide (eNO) expiration as a sign of inflammation of the respiratory tract in children. The analysis showed a strong correlation between asthma with the exposure to nitric oxide (NO) and other toxic air. Our objective is to verify the hypothesis which stated that children who live in shelters around industrial areas and are exposed to air pollution will experience more respiratory tract irritation than children who do not live in the surrounding district of the industrial area, confirmed by increased concentrations of eNO.

*Methods*

Case controls and data collection were held in Jakarta to investigate the effects of the environment around the plant with increased exposure of FeNO, and data from the area of East Nusa Tenggara, Lombok, Indonesia were used as a control.

*Results*

Children whom are often exposed to toxic air pollutants can undergo an inflammatory process and this may cause a decrease in vital lung function. This was supported by evidence found from the increasing eNO concentrations and derivation of expiratory peak flow rate (PEFR) in children who live around industrial areas under study.

*Conclusions*

There are strong correlations between asthma in children with the exposure of toxic factory air pollution. Others factors that may increase asthma include (1) the size and width of the house, (2) the humidity of the house, (3) the lighting of the house, (4) the ventilation of the house, and (5) mother's education and knowledge.

### Children's Environmental Health and Opportunities for Action in the Sustainable Development Goals Era

Maria Neira

Department of Public Health, Environment and Social Determinants of Health, World Health Organization, Geneva, Switzerland

Corresponding author:
Neira, Maria
Geneva, Switzerland
brunedrissem@who.int

The Sustainable Development Goals (SDG) present an opportunity for the health sector to work with other fields in reducing environmental hazards to children. What children experience in the first five years of life is the foundation for their future health; SDG 3, which emphasizes the importance of well-being at all ages, depends on this critical period. Healthy lives must be nurtured in healthy environments, but environmental influences to children's health extend far beyond SDG 3. Of the 5.9 million deaths of children under five each year, 26% are attributable to the environment, deaths which can only be prevented with multilateral action.

Children need access to clean water and sanitation to be healthy; action to address water, sanitation and hygiene issues will not only work towards the goals of SDG 6, but also prevent child deaths from diarrhoea, malnutrition and vector borne disease. Children should breathe fresh air and live pneumonia-free with clean energy for home and industrial use, the focus of SDG 7. They must be at the center of urban planning, addressed in SDG 11, as safe roads, buildings and public spaces can prevent injury and encourage healthy activity among the young. It is crucial for children's development and well-being that early toxic exposures are prevented and that SDG12 on sustainable production and consumption is achieved. Children's special vulnerability is also a great motivator for SDG 13: take urgent action to combat climate change and its impacts, as these impacts, including extreme weather and increased levels of asthma-inducing pollen and mould, are most harmful to children.

Improving children's health and reducing environmental risks are at the heart of sustainable development.  Every SDG relates to an element of children's environmental health, and multisectoral collaboration has the potential to improve the lives of so many. Children's futures are the strongest motivation for all SDGs, and should inspire the multisectoral efforts that will bring about a healthier world, free of environmental hazards.

### Prenatal Exposure to Particulate Matter and the Occurrence of Wheezing in the Chest in Children

Agnieszka Pac (1), Renata Majewska (1), Frederica P. Perera (2)

(1) Chair of Epidemiology and Preventive Medicine, Jagiellonian University Medical College, Krakow, Poland
(2) Columbia Center for Children's Environmental Health, Columbia University, New York, USA

Corresponding author:
Pac, Agnieszka
Krakow, Poland
agnieszka.pac@uj.edu.pl

*Background*

Several studies have found associations between air pollution levels and respiratory symptoms occurrence. We hypothesized that prenatal exposure to particulate matter ($PM_{2.5}$) may be associated with respiratory symptom (especially wheezing) occurrence in children.

*Material and methods*

The subjects come from the birth cohort study from Krakow, Poland. Air pollution was measured with personal samplers for $PM_{2.5}$ over a 48 hour period in the end of second trimester of pregnancy. The newborns and later children were examined regularly up to 9 years of age. Parents were concerned about their child's heath, especially about symptoms related to the respiratory tract. Overall, the data for full 9 years of follow-up were available for 272 children (135 boys). The impact of $PM_{2.5}$ on the number of days of wheezing symptoms was adjusted for other known factors of respiratory diseases, using a zero-inflated Poisson regression model. Ethical approval was granted by the Bioethical Committee of the Jagiellonian University.

### Results

Wheezing symptoms were present for 18.8% of children during the first year of their life, and was decreased for the next years to 4.8% for nine year olds. The risk of wheezing episodes was significantly related to $PM_{2.5}$ level during the first four years of life: during the first year incidence rate ratio =1.010; 95% confidence interval: 1.007-1.013 for 1 ug/$m^3$ of $PM_{2.5}$; and this impact was decreased to an incidence rate ratio =1.0006 (95% confidence interval: 1.0003-1.0009). Later, this impact was no longer significant.

### Conclusions

Prenatal exposure to $PM_{2.5}$ pollution can lead to higher occurrence of wheezing symptoms among children. However, this association was weaker with time.

---

### Empowering Youth and Communities to Promote Health and the Environment — The Bangalore Experience

Elizabeth Cherian Paramesh (1,2,3), Roshan Cherian Paramesh (1), Rashmi Cherian Paramesh (1)

Director, Lakeside Center for Health Promotion
Alliance for Health Promotion, Geneva, Switzerland
Zonal Secretary, Rotary District 3190

Corresponding author:
Paramesh, Elizabeth Cherian
Bangalore, India
elizabethcherianp@gmail.com

### Background

In the quest to promote health and protect the environment it has been our experience that the power of youth can impact communities and the results are far reaching and sustained. In the process of working with youth from underprivileged communities we are creating future generations who can ensure a responsible society who will protect the planet and leave behind a legacy for the children of tomorrow to inherit.

### Methods

The primary objective of our efforts was to promote community responsibility and action for health promotion. The secondary objectives were to create self responsibility for health, focus on issues of livelihood, health and safety concerns, to promote need based social and health intervention and research, protect the environment, promote health and also to establish a network of health professionals, policy makers and the public. For the past few years we have constantly strived in this direction and have had several experiments that have yielded very positive results encouraging us to make our services widespread, accessible and affordable. The media has supported the propagation of health practices relevant for the time and needs of the specific population. All of our initiatives in communities have been followed up with impact studies and the results have been highly encouraging.

*Results*

Some of our activities included the setting up of twenty-one schools in three years in villages in and around Bangalore in communities where children did not have an option but to continue in the traditional sculpting profession of the elders as there were no schools in the nearby vicinity. Today an impact study reveals that 65% of the children of this village are being educated in the schools that we have built for them. We also had the local policy makers involved and today there is community responsibility for the cause of education.

The solar lighting project of the Menda Foundation has been another significant step in lighting the dark homes of underprivileged sections of society. We have installed solar panels in schools, hostels and homes of agricultural workers to ensure the safety and security of the people along with additional subsidies for parents of girls to promote the education of girls in particular. An impact study revealed a 24% increase in the admission of girls to schools. In the State of Karnataka, 800 student hostels have been lit up and 15,000 students have benefited. Girls are now spared hard work in the fields and are getting educated. Low cost toilet blocks have been put up in schools with no proper toilet facilities and girls are again back to school. A low cost sanitary napkin making machine has been set up in the Bangalore Central Prisons for the benefit of women inmates and girls and follow-up studies have shown a reduction in genitourinary tract infections by 33% in less than one year. Another project, called Asha Kiran, is about getting one child back to school at a cost borne by a donor. These are children who are in bonded labor and destitute who now have their right to education and a happy childhood restored. Youth have been motivated to conduct blood donation camps and a record 3000 units of blood are regularly collected monthly from voluntary donors - college students and youth in particular to be given free for those who cannot afford the cost. We conduct these camps in association with the blood banks who provide the logistics free of cost. We have started a program called WASH in SCHOOLS which has reduced infections significantly in children going to school where they previously never had a hand washing facility. We have raised funds for supporting dialysis costs of poor renal failure patients. We also conduct free health camps for children in schools and ensure their timely immunisation. Free midday meals are provided to children in all our schools thereby catering to their nutritional needs. We also identify exceptional talent in children and youth and promote their training in areas of their interest.

There are several other initiatives that we have undertaken and these efforts are directed to the promotion of education and literacy, provision of a clean environment, health care, nutritional support of children and the welfare of underprivileged children in particular.

*Conclusions*
Interventions for promoting health and protecting the environment need to be issue and need based and tailored to suit particular communities. Involvement of communities in addressing the issue of health promotion and protection of environment is imperative and the results are far reaching and sustainable. The interplay of stake holders has also ensured effective services and promoted community responsibility. Involvement of youth in our community welfare programs have enabled the creation of responsible future citizens and leaders. The involvement of corporate sectors has been an added incentive for the funding of these projects. Positive significant changes in lifestyle and health practices can develop a nation of healthy and productive individuals. Accessibility to health, good nutrition, safe environment and education is everyone's right. Igniting the passion among youth to serve the underprivileged in society ensures continuity and responsibility. We intend to widen our network to involve more stake holders, NGOs and governmental agencies and reach out to the remotest areas in communities.

---

### Dietary Environment in Preventing Allergic Airway Diseases

Haralappa Paramesh (1,2,3) , Roshan Cherian Paramesh (1)

(1) Lakeside Center for Health Promotion, Bangalore, India
(2) Alliance For Health Promotion, Geneva, Switzerland
(3) Pediatric Pulmonologist and Environmentalist

Corresponding author:
Paramesh, Haralappa
Bangalore, India
drhparamesh@gmail.com

*Background*
Allergic airway disease is increasing in prevalence globally. Asthma is a global health epidemic and over 1 billion people currently suffer and this is anticipated to increase to about 4 billion in 2050. Air pollution causes 8 million deaths globally.

*Methods*
Over the years our objectives have been to control the symptoms of allergic disease and improve quality of life focusing on bronchoconstriction, airway hyper-responsiveness, inflammation and remodelling, the united airway concept, phenotypes and genetics. The current focus is on dietary habits to prevent airway allergies and reduce the morbidity, mortality and socioeconomic burden of these diseases.

*Results*
Diet plays a multifaceted role in shaping the observed world-wide trends of childhood allergies and in particular airway diseases. Sensitivity can occur by ingestion or inhalation of allergens. Both IgE and non IgE mediated immune responses and both early and late phase reactions are observed.

Dietary measures to prevent airway allergies are reviewed with regard to diet in pregnant mothers, breastfeeding of infants and diet. Diet in children with airway disease, dietary restrictions, and links between obesity and asthma are also addressed.

Maternal Dietary Intention:  Maternal diet rich in antioxidants during pregnancy has the potential to influence postnatal susceptibility to allergic diseases by modulatory T cell response. However, strict elimination of essential foods like milk, egg etc. may have an adverse effect on maternal and fetal nutrition. Meta-analysis is not recommended at this stage.

Diet of infants and Breast feeding:  It is recommended to continue breast feeds for 6 months and add solids after 6 months. Emerging data suggest that early introduction of allergic foods may reduce the risk of food allergy.

Diet for Allergic Children:  Diet rich in antioxidants like fruits, vegetables, vitamins, curds, butter, fish and Omega 3 fatty acids and home-made food is recommended

Diet to be avoided in allergic children:  Salty food snacks, take away food consumption, and soft drinks containing preservatives or colorants.

Obesity and Asthma:  Obesity is increasing in India. Between, 2002-2011 it increased from 15-20% in boys and 13.7-18.1% in girls. Decreased lung function is proportionately related to obesity. Asthmatic children should reduce the intake of gas producing food items like asparagus, broccoli, cabbage, garlic, melons, peppers, radish, cauliflower, cucumbers, raw onions, beans etc.

### *Conclusions*

Epidemiology and tradition strongly suggest the association of nutrition and airway allergy, but we lack robust scientific data to make national policy. Traditional local food habits should be encouraged along with disease guidelines to be followed to manage disease and costs can be cut by two thirds. For better compliance, links between patients, parents and other health providers is essential as well as science to progress the link between practitioners, academicians, researches and pharmaceutical companies.

### Rapid Urbanization and Changing Prevalence of Chronic Cough

Haralappa Paramesh (1,2) Rashmi Cherian Paramesh (1)

(1) Lakeside Center for Health Promotion
(2) Alliance for Health Promotion, Geneva, Switzerland

Corresponding author:
Parameshwarappa Haralappa
Bangalore, India
drhparamesh@gmail.com

*Background*

Cough is a reflex response to mechanical, inflammatory or chemical irritation of the respiratory tract predominantly and also a social signal as well. Depending on duration, cough may be considered to be chronic if it lasts at least 2 to 8 weeks. The present study considered cough to be chronic if it lasted more than two weeks.

Chronic cough has a tremendous socio-economic impact on children. It produces sleep disturbance, exhaustion, nausea, vomiting, urinary incontinence, rectal prolapse, fecal soiling, subconjunctival hemorrhage, hernia, cerebral hypoxic encephalopathy, and cough syncope. Parents often feel frustrated, upset, helpless and may experience sleepless nights and financial burden. In the present study, 80% of children had five consultations for cough.

*Methods*

The aim of our study is to determine the prevalence and spectrum of chronic cough and to correlate our findings with earlier data and city demographic changes.

We analysed 510 patients referred for pulmonary problems between Jan 2015 to March 2016. Among these patients, 106 patients came just for cough over 2 weeks duration. A detailed history including environmental history, clinical examination and laboratory testings, imaging, pulmonary function were performed as needed according to clinical judgement and patient capacity to perform pulmonary function testing.

*Results*

The prevalence of chronic cough was 105 out of 510 cases (20.78%). Wet cough and night/early morning cough were present in 80% and 84% of cases, respectively. The majority were due to allergic rhinosinusitis and asthma, and 3 cases each of bronchiectasis, pertussis, and psychogenic origin.

In 1999, the prevalence of chronic cough was 8%, and this has risen to 20.78%. In 2010, the prevalence of chronic cough was reported to be 10.5%. The population in Bangalore was 2.92 million in 1981, and had risen to 8.5 million in 2011. Traffic congestion has also increased. A total of 1,600 vehicles are added every day. A road length of 13,000 km would be needed to park all vehicles bumper to bumper. Vehicle now average 9.6 km per hour. Slow vehicles emit 5.5 times more emissions than vehicles moving at 70 km/hr. In summer there is greater production of ozone, slow wind velocity, and higher automobile emissions. Air pollution produces oxidative stress on airways, causing smooth muscle contraction, epithelial damage, altered release of inflammatory mediators and produces hyper-reactive airways and chronic cough.

### Discussion

The prevalence of chronic cough in children was 8% in 1999 and has increased to 20.78%. A community survey by same group in the year 2010 on point prevalence of chronic cough was 10.5%. The Bangalore city population was 2.92 million in the year 1981 it was increased to 8.5 million in the year 2011.

Traffic congestion is increased. There were 5,875,237 vehicles in Oct 2015. Of these, 406,456 are two wheelers, 11,44,196 are cars, and 569,579 are buses. Now we add 1,600 new vehicles every single day. Previously, from 2009-2010, there were 600 vehicles per day. We would need 13,000 km length of roads to park our vehicles bumper to bumper. The speed of vehicles now is 9.6 km per hour.

Slow vehicles emit 5½ times more emissions to the atmosphere than vehicles at 70 Km/hr speed. In summer time there is greater production of ozone in bright sun, slow wind velocity and higher automobile emission. Air pollution produces oxidative stress on airways causing airway smooth muscle contraction, epithelial damage, excess mucus production, decreased ciliary movement, increased permeability of vessels, altered release of inflammatory mediators and airway hyper-reactivity and chronic cough.

### Conclusions

Rapid urbanization and demographic changes have increased the number of slow moving vehicles, with greater emissions producing oxidative stress and airway hyper-reactivity, leading to chronic cough. Chronic cough has increased from 8% to 20.78% over a period of 17 years. Along with proper medications, measures to mitigate air pollution should be taken with all stakeholders.

---

### Descriptive Analysis from a Hospital-Based Childhood Cancer Survivors Cohort: Report From the Project Spain-CCSS

Elisa Pasqual (1,2,3), Héctor Salvador Hernàndez (4) Lourdes Arjona Camì (1,2,3), Mariona Gil Cerdan (1,2,3), Miguel Ángel Flores Taico (4), Ofelia Cruz Martínez (4), Elisabeth Cardis (1,2,3)

(1) Barcelona Institute for Global Health, Centre for Research in Environmental Epidemiology, Barcelona, Spain
(2) Universitat Pompeu Fabra, Barcelona, Spain
(3) Centro de Investigación Biomédica en Red de Epidemiología y Salud Pública, Madrid, Spain
(4) Hospital Sant Joan de Deu, Barcelona, Spain

Corresponding author:
Pasqual, Elisa
Barcelona, Spain
elisa.pasqual@isglobal.org

*Background*

Therapeutic advances in pediatric oncology have led to an increase the number of childhood cancers survivors (CCSs). Emerging evidence shows that CCSs experience long-term morbidity due to their cancer therapy (and the cancer itself). The Spain-CCSS (Spanish Childhood Cancer Survivors Study) aims to set up a childhood cancer survivor's cohort in Spain in order to join international efforts for studying long term health effects among this population and optimise treatment and follow-up.

*Methods*

CCSs treated between 1980 and 2009 in the pediatric hemato-oncology department of Hospital Sant Joan de Deu in Barcelona (one of the largest in Spain) were invited, via mail services and by phone call, to participate in the study and to complete a health status questionnaire (HSQ). Recruitment, disease validation and retrospective record collection (treatment) are still ongoing. Ethical approval was granted by the ethical committees of Hospital Sant Joan de Dèu and Parc de Salut Mar.

*Results*

Of over 1568 individuals who survived more than 5 years, 536 (34%) consented to participate (509 returned a complete HSQ), 83 (5%) were not alive at the time of the questionnaire, 480 (30%) could not be traced, and 140 (6%) refused to participate. We are still waiting for a response from 340 (30%) participants.

Among the total participants, 285 (53 %) are male. Participants' median age is 22 years (range 6-50) and the majority (N = 311; 58%) have been treated recently (from 2000-2009). The median follow up is 13 years (range 5-35). The median age at diagnosis is 5 years (range 0-19). Central nervous system tumour (N= 120, 22%) and leukaemia (N= 113, 21%) are the most prevalent primary neoplasm. Information collected through the HSQ includes socio-economic status and quality of life health related outcomes.

*Conclusions*

Though more effort is needed to increase the participation rate, this cohort is an important source of information on different health outcomes among CCSs.

### Cognitive Effects After Low- to Moderate Dose Exposure: Study Plan in a Cohort of Childhood Cancer Survivors

Elisa Pasqual (1,2,3), Héctor Salvador Hernàndez (4), Laura Mangado Aloy (4), Cristina Boix LLuch (4) Ofelia Cruz Martínez (4), Elisabeth Cardis (1,2,3)

(1) Barcelona Institute for Global Health, Centre for Research in Environmental Epidemiology, Barcelona, Spain
(2) Universitat Pompeu Fabra, Barcelona, Spain
(3) Centro de Investigación Biomédica en Red de Epidemiología y Salud Pública, Madrid, Spain
(4) Hospital Sant Joan de Deu, Barcelona, Spain

Corresponding author:
Pasqual, Elisa
Barcelona, Spain
epasqual@isglobal.org

*Background*
Brain cancer survivors are at higher risk to develop a cognitive deficit and cranial radiotherapy (RT) is one of the well-known risk factors of this impairment.

However, the effect of low dose radiotherapy (such as the scatter radiation that is absorbed by the brain when radiotherapy is delivered in another anatomical region) still needs to be clarified and more research is warranted in this area.

*Methods*
We are planning to evaluate the neuro cognitive profile in a cohort of childhood cancer survivors who received a dose below 5 mGy to the brain. Brain tumor survivors will be excluded in order to avoid confounding due to the cancer itself. Function will be assessed in different cognitive domains (intelligence, executive function, processing speed, memory, attention and executive function among others) and the score of each test will be correlated with the dose absorbed in different anatomical brain structures (with matter, hippocampus, temporal lobe, frontal lobe and posterior fossa) as estimated from medical records. We haven't yet submitted the protocol to an ethics committee, as we are still designing the study.

*Results*
Assuming that around 40% of survivors did not received radiotherapy, 35% received a dose below 1 Gy and 5% between 1 and 5 Gy (de Valthaire personal communication) and assuming a 4 point difference in the test score (average 100, standard deviation 15) between dose groups, we expect to have 80% power to find a trend among those three groups with around 100 participants. To reach 80% power to find a 4 point difference between the group who did not receive RT and the ones who received a dose below 1 Gy, we would need around 500 patients.

*Conclusions*
It appears to be feasible – and informative for radiation protection – to study cognitive effects due to low-to moderate doses of radiation in a cohort of childhood cancer survivors.

### Determining the Impact of Smoking Point of Sale Legislation Among Youth (Display) Study: New Findings From Scotland

Jamie Pearce

Centre for Research on Environment, Society and Health, University of Edinburgh, Edinburgh, United Kingdom

Corresponding author:
Pearce, Jamie
Edinburgh, UK
Jamie.Pearce@ed.ac.uk

*Background*

Point of sale displays are one of the most important forms of tobacco marketing still permitted in many countries. Point of sale displays have been shown to increase susceptibility to smoking, experimentation and initiation into smoking, particularly amongst adolescents. These displays may also influence perceived prevalence of smoking and the perception that tobacco products are easily obtained and are a 'normal' product.

*Methods*

This study examines the impact of the implementation of both a partial and comprehensive ban on point of sale tobacco advertising on attitudes to smoking, brand awareness, perceived ease of access to tobacco products and youth smoking prevalence. The study has a multi-modal before and after design and uses mixed methods to collect data, at baseline and then with longitudinal follow-up for 4 years, in four purposively selected communities. In each of the four communities (school catchment areas), at baseline and in follow-up years, data collection includes: mapping and spatial analyses of tobacco retail outlets; tobacco advertising and marketing audits of tobacco retail outlets most used by young people; cross-sectional school surveys of secondary school pupils; and focus group interviews with purposive samples of secondary school pupils. The tobacco audit is supplemented by interviews and observations conducted with a panel of tobacco retailers recruited from four matched communities.

*Results*

The results to be presented from the initial phases of data collection consider: the changing levels of point of sale exposure over the study period; the degree of compliance with the new legislation post implementation; connections between point of sale displays and young people's brand awareness; the emergence of e-cigarette marketing; and the connections between e-cigarette/cigarette marketing and e-cigarette use amongst young people.

*Conclusions*

The results will be of considerable interest to policy makers both from the UK and other jurisdictions where they are considering the development and implementation of similar legislation.

### The Role of Local Natural Space in Children's Social, Emotional and Behavioural Development in Scotland: a Longitudinal Study

Elizabeth A. Richardson, Jamie Pearce, Niamh K. Shortt, Richard Mitchell

Centre for Research on Environment, Society and Health, University of Edinburgh, Edinburgh, United Kindom

Corresponding author:
Pearce, Jamie
Edinburgh, UK
Jamie.Pearce@ed.ac.uk

*Background*

Poor mental health in childhood has implications for health, wellbeing, and productivity in later life, as well as presenting a considerable burden for families and wider society. Independent play is a crucial part of 4–6 year olds' social, emotional and behavioural development. We investigate whether total natural space, public parks and access to a private gardens enhance children's social, emotional and behavioural development.

*Methods*

We analysed longitudinal data for 2,909 urban-dwelling children (aged 4 at 2008/9 baseline) from the Growing Up in Scotland survey. Social, emotional and behavioural difficulties were scored using the Strengths and Difficulties Questionnaire (divided into four domains), and area (%) of total natural space and parks within 500 m of the child's home was quantified using Scotland's Greenspace Map. Access to a private garden was captured at the household level. We ran repeated-measures multilevel models, then stratified by sex or household educational attainment.

*Results*

Prosocial behaviour scores were higher (i.e., better) throughout for children with more total natural space, but neighbourhood natural space was unrelated to change over time of any Strengths and Difficulties Questionnaire domain. Boys and girls in neighbourhoods with more total and park space, respectively, had fewer problems. Better social outcomes were found for all groups with more neighbourhood natural space. Having access to a garden was related to the largest benefits for all except those from high education households. Having access to a garden was related to mental health benefits on a par with the advantage apparent for girls over boys, children from degree-educated households over those from households without educational qualifications, or a £20,000 to £50,000 increase in equivalised household income.

*Conclusions*

Neighbourhood natural space may reduce social, emotional and behavioural difficulties for 4 to 6 year olds, but private garden access may be most beneficial.

### Modifiable Factors Related to Low Water Intake In Mexican Children and Adolescents

María de los Angeles Peña-Farfán, Donovan Raúl Ríos-Hernández, Arely Vergara-Casteñada, Laura Martino-Roaro

Research Group Food and Health, Faculty of Chemistry, La Salle University, Mexico City, Mexico

Corresponding author:
Peña-Farfán, María de los Angeles
Mexico City, Mexico
angie.pefa12@gmail.com

*Background*

Proper hydration is important for maintaining homeostasis in all physiological processes and to avoid some diseases. Some reports have suggested that 87.5% of males and 65.4% of females have water intake below the recommendations. In children, there is a lack of information about hydration habits. This study was aimed to assess modifiable factors related to low water intake in a Mexican sample of adolescents and children.

*Methods*

A cross sectional study was performed. Cardiovascular risk factors were determined (overweight, obesity, abdominal obesity). Physical activity (frequency and type) and dietetic habits such times of eating per day were assessed. Statistics were performed using SPSS Version 22.0. Ethical approval was granted by Ethics Committee and Research from Mexican School of Medicine, Univesity La Salle.

*Results*

247 children and adolescents were included; the average age was 12.5±1.9 years old and boys made up 54.91% of the sample.. The global prevalence of overweight and obesity was 49.2%. The daily intake of water, including foods, was 1317.5 ± 648.8 ml, with no differences between boys and girls or according to the presence of cardiovascular risk factors. Only 12.1% reach the recommendations of water intake according to Mexican recommendations. Also, water intake was not different between those who practice any physical activity more than 3 days per week compared with those physically actives (1163.3±571.3 vs 1339.6±697.9, respectively; p=0.181) and considering the type of activity (aerobic or anaerobic; p>0.05). According to times of eating, those who eat less than 3 times per day reported lower water intake (778.6±477.2 vs 1342.0±688.0; p=0.033) lunch and the afternoon snack being most important, and seem to risk of not accomplishing the recommendations (odds ratio=1.148 (1.093-1.206).

*Conclusions*

There is a high prevalence of cardiovascular risk factors among Mexican adolescents. Low water consumption was reported in this population and it seemed not to be related to anthropometric indicators, suggesting that environmental factors such number of meals could be more important than nutritional status or the practice of physical activity.

### Pilot Phase of the Next Generation Study

Pavel Piler (1), Lenka Andrýsková (1), Vít Kandrnal (1), Tomáš Prša (1), Richard Hďek (1,3), Martin Tomáš (2), Petr Jankč(4), Jana Klánová (1)

1) Research Centre for Toxic Compounds in the Environment (RECETOX), Faculty of Science, Masaryk University, Brno, Czech Republic
2) Institute of Computer Science, Masaryk University, Brno, Czech Republic
3) Institute of Biostatistics and Analyses, Masaryk University, Brno, Czech Republic
4) Department of Obstetrics and Gynecology, University Hospital Brno, Medical Faculty of Masaryk University, Brno, Czech Republic

Corresponding author:
Piler, Pavel
Brno, Czech Republic
piler@recetox.muni.cz

*Background*
The Next Generation Study is a new prospective birth cohort initiated as a collaborative project of Masaryk University and the University Hospital in Brno, Czech Republic. The study is designed to follow up 7000 children from their prenatal period to adolescence with the aim of assessing multiple factors potentially affecting children's health (exposome factors).

*Methods*
Special attention is being paid to prenatal and early postnatal developmental abnormities, postnatal psychomotor development, neurobehavioral disorders and immune system disorders. Information on complex exposures will be based on on-line versions of a combination of validated on-line questionnaires and biological tissue analyses will be used to estimate complex exposures. Information on health outcomes will be taken from the psychomotor developmental screening questionnaires, medical records, and health registers. Ethical approval was granted by The University Hospital Brno.

*Results*
The Next Generation Study was initiated in April 2015 with a three-stage pilot. Feasibility of the cord blood sample collection and processing protocol was assessed at the first stage on 100 mother–child pairs. The aim of the second stage was to evaluate feasibility of the protocol for recruitment of women in the 38th week of pregnancy. That allowed for estimation of future study response rates. Samples of venous blood, urine, and saliva were collected from 150 participating mothers, and stool and saliva were taken from newborns in addition to cord blood samples. On-line versions of the self-reported questionnaires will be tested at the third stage (scheduled to start in August 2016) together with robustness of the IT systems.

A majority of the approached mothers was willing to participate in the Next Generation pilot study (90% of mothers were willing to donate venous blood in 38th week of pregnancy, 70% agreed to donate cord blood, to fill the questionnaire and provide health data). The sample collection and processing protocol were proven to be feasible: all types of samples were processed and stored within 180 minutes regardless the day and time. Whole blood, plasma, serum, red cells, and buffy coat will be stored at -80°C, viable cell lines from cord blood will be stored in liquid nitrogen.

### *Conclusions*

Based on the results of the pilot phase the full scale TNG study is scheduled to start in January 2017.

---

### Maternal Lifestyle During Pregnancy and Child Neurodevelopment

Kinga Polanska (1), Anna Krol (1), Wojciech Sobala (1), Jolanta Gromadzinska (2), Renata Brodzka (2), Gemma Calamandrei (3), Flavia Chiarotti (3), Dorota Merecz-Kot (4), Teresa Makowiec-Dabrowska (5), Danuta Ligocka (6), Wojciech Wasowicz (2), Wojciech Hanke (1)

(1) Department of Environmental Epidemiology, Nofer Institute of Occupational Medicine, Lodz, Poland
(2) Department of Biological and Environmental Monitoring, Nofer Institute of Occupational Medicine, Lodz, Poland
(3) Unit of Neurotoxicology and Neuroendocrinology, Department of Cell Biology and Neuroscience, Istituto Superiore di Sanità, Rome, Italy
(4) Department of Health and Work Psychology, Nofer Institute of Occupational Medicine, Lodz, Poland
(5) Department of Work Physiology and Ergonomics, Nofer Institute of Occupational Medicine, Lodz, Poland
(6) Bureau of Quality Assurance, Nofer Institute of Occupational Medicine, Lodz, Poland

Corresponding author:
Polanska, Kinga
Lodz, Poland
kinga@imp.lodz.pl

### *Background*

Substantial attention has been paid in recent years to the impact of environmental factors on child development. The objective of this study was to evaluate the association between maternal lifestyle factors and child neurodevelopment.

### *Methods*

The current analysis included mother–child pairs from the Polish Mother and Child Cohort study (REPRO_PL). The following factors related to maternal lifestyle were considered: smoking (based on cotinine level), phthalate exposure based on their metabolite levels in urine, microelements levels in maternal and cord blood, alcohol consumption, leisure-time physical activity and stress in pregnancy, pre-pregnancy body mass index and folic acid supplementation based on questionnaire data. Psychomotor development was assessed in children at the ages of one and two by the Bayley Scales of Infant and Toddler Development. Ethical approval was granted by the Ethical Committee of the Nofer Institute of Occupational Medicine, Łódź, Poland and written consent was obtained from all subjects.

### Results

A significant association was observed between prenatal exposure to tobacco constituents and decreased child motor development in assessments performed at both ages ($\beta=-0.8$, $p=0.01$; $\beta=-1.4$, $p=0.001$). Child motor development was also inversely associated with natural log concentrations of most of the phthalate metabolites ($p<0.05$). Maternal pre-pregnancy underweight was associated with decreased child language abilities ($\beta=-5.2$, $p=0.01$). Negative impact on child cognitive development at the age of two was observed for the Perceived Stress Scale ($\beta$=-0.8, $p=0.01$) and the Social Readjustment Rating Scale ($\beta$=-0.4, $p=0.03$). The recommended level of leisure-time physical activity during pregnancy was beneficial for child language development ($\beta=4.8$, $p=0.02$). Finally, a positive association between selenium levels in pregnancy and motor development ($\beta=0.2$, $p=0.002$) at one year of age and language development ($\beta=0.2$, $p=0.03$) at two years of age was observed. For alcohol and folic acid there were no significant associations with any of the analyzed domains of child neurodevelopment.

### Conclusions

These results underscore the importance of policies and public health interventions promoting healthy lifestyle among women in reproductive age and during pregnancy.

---

### Prenatal Exposure Assessment Approaches Need to Consider Spatio-Temporal Variation of Air Pollution

Andrea Ranzi (1), Serena Broccoli (2), Michele Cordioli (1), Federica Parmagnani (1), Silvia Candela (2), Elisa Pollini (3), Valeria Frassineti (4), Cristina Raineri (4), Patrizia Lucialli (3), Paola Angelini (5), Silvi Giuliano (4), Lauriola Paolo (1)

(1) Environmental Health Reference Centre, Regional Agency for Prevention Environment and Energy of Emilia-Romagna Region, Modena, Italy
(2) Inter-institutional Epidemiology Unit, Arcispedale Santa Maria Nuova, Research Hospital for Advanced Technologies and Clinical Pathways in Oncology, Reggio Emilia, Italy
(3) Regional Agency for Prevention Environment and Energy of Emilia-Romagna region, Ravenna, Italy
(4) Public Health Department, Local Health Authority, Ravenna, Italy
(5) Public Health Service, Emilia-Romagna Region, Bologna, Italy

Corresponding author:
Ranzi, Andrea
Modena, Italy
aranzi@arpae.it

### Background

Air pollution exposure during pregnancy may have consequences for for the unborn child's life and the future generation. The time-window of exposure (intermediate between short- and long-term effects) poses a methodological problem in the choice of the most suitable exposure assessment approach.

The objective of the present study was to evaluate the effect of different prenatal exposure assessment approaches on reproductive outcomes.

### *Methods*

In the Emilia-Romagna region (Northern Italy) two independent studies (Moniter and Sinatra projects) were conducted on air pollution and several reproductive outcomes. The first assessed the residential exposure to incinerator pollution of mothers living in areas next to plants; the latter considered different factors of environmental pressure in an urban-industrial city (Ravenna). Each newborn was georeferenced and characterized by a specific level of exposure emissions, and categorized in quartiles of $PM_{10}$. Modeling approaches were adopted for exposure assessment, namely an ADMS-Urban dispersion model for each type of environmental determinant, to account for both spatial (residential street number) and temporal (monthly simulations) variability of exposure. Both studies showed statistical associations only with preterm births. Analyses were repeated considering poorest indicators of exposure, to verify the influence of exposure assessment approach on final results.

### *Results*

Considering the best method of exposure (spatio-temporal variability of air-pollution), both studies showed a significant association between preterm births (PTB) and exposure to incinerators and port activities. The exclusion or an inadequate assessment of the temporal variability of the exposure led to a reduction of the associations and loss of statistical significance in RRs (from 1.30: CI95% 1.08-1.57 to 1.02: CI95% 0.88; 1.25 and from 1.52: CI95% 1.01-2.29 to 1.01: CI95% 0.66-1.55) respectively, considering the highest versus the lowest level of exposure.

### *Conclusions*

Air pollution from industrial sources is associated with PTB. Our experience confirms the need of exposure models capable of capturing both the spatial and temporal variability of air pollution exposures, in order to be able to detect associations with birth outcomes.

### Air Pollution and Children's Environmental Health Action in Brazil

Emerson Rodrigues da Silva

University of Caxias do Sul, Caxias do Sul, Brazil

Corresponding author:
Rodrigues da Silva, Emerson
Caxias do Sul, Brazil
ersilva9@ucs.br

### *Background*

Air pollution is estimated to cause around 800 deaths in children under 5 years of age in low- and middle-income countries of the Americas. Although it is not the worst scenario compared to regions like

Africa or the Eastern Mediterranean region, this rate may be underestimated because environmental awareness and air pollution monitoring are extremely scarce, even in middle-income countries like Brazil.

*Methods*

A quick review of children's environmental health in Brazil is presented, highlighting some issues regarding risk awareness and current environmental threats.

*Results*

Around 19 million people use biomass for cooking or heating purposes in Brazil, and the country has one of the highest rates of pediatric asthma as evidenced in worldwide epidemiologic studies. The lack of awareness about children's environmental health in low- and middle-income countries is not exclusive to air pollution: children's access to unsafe water, the emergence of vector-borne diseases (like the triad Zika / Dengue / Chikungunya viruses), manmade disasters, and unsafe buildings in landslide areas are problems that occur frequently, mostly due to the lack of proper risk perception. Another example is the fact that Brazil has led the use of pesticides in the world since 2008 and yet concerns about children's ingestion of pesticides are rare. Efforts to increase awareness (not only about air pollution) are urgently needed, not only in Brazil, but probably in most low- and middle-income countries. On the other hand, children's exposure to indoor pollution due to second-hand tobacco smoke has been reduced consistently in Brazil over the last 2 decades, after a strong action of all stakeholders and a long-lasting governmental campaign. This successful case shows that it is indeed possible to increase awareness about other children's environmental health issues as long as all players work together.

*Conclusions*

In an effort to collect data, help policy makers, increase research possibilities, and create training programs for health workers, the WHO has recently created a Collaborating Center for Children's Environmental Health in the South of Brazil with the goal of helping improve this scenario.

---

### Children's Environmental Health Training in Brazil

**Emerson Rodrigues da Silva**

**University of Caxias do Sul, Caxias do Sul, Brazil**

**Corresponding author:**
**Rodrigues da Silva, Emerson**
**Caxias do Sul, Brazil**
**ersilva9@ucs.br**

*Background*

Due to their unique characteristics, children are more vulnerable to environmental hazards than adults. The environment is responsible for one third of the global burden of disease in children, but there is a lack of proper awareness of such risks in many countries.

### *Methods*
To highlight the current state of environmental pediatric training in Brazil, and describe ongoing strategies to address problems.

### *Results*
The environmental burden of disease in children is not equally distributed throughout the world: the heaviest burden lies in low- and middle-income countries. To prevent, assess and treat environmental problems, it is essential to understand the hazards and physiologic characteristics particular to children. Unfortunately, many pediatricians still lack knowledge and awareness regarding environmental health issues in low- and middle-income countries. In Brazil, for example, 79% of pediatricians have never received any kind of training regarding children's environmental health. Only 17% declared they knew about the risks of lead intoxication in children, a concern included long ago in the routine pediatric care in developed countries.

In Brazil, the inclusion of children's environmental health in the pediatric curricula during residency is rare. Pediatric departments of universities lack specific divisions or even pediatricians able to share information and promote environmental health. The Brazilian Society of Pediatrics is the third largest in the world, with 22.000 affiliates. It has 33 different departments, but no one dedicated to children's environmental health (although some of them are in related areas, like toxicology).

### *Conclusions*
The pediatric curricula in residency programs in Brazil have a giant gap that urgently needs to be fixed. To help with this, the WHO and the Collaborating Center for children's environmental health in the south of Brazil are working to disseminate training programs in the country, based on 29 training modules developed by the WHO and translated into Portuguese by the Collaborating Center. In addition, partnerships with governmental administrators and pediatrics societies are being established. The subject was formally introduced in some post-graduate and residency programs in the south of Brazil.

---

### Burden of Congenital Malformations Possibly Due to Infectious Disease: The Case of Zika Virus and Microcephalia in Brazil

Roberto Ronchetti

Department of Pediatrics, Second School of Medicine, University La Sapienza, Rome, Italy
President of Rome Department of the ISDE (Int Soc of Doctors for the Environment), Rome, Italy

Corresponding Author:
Ronchetti, Roberto
Rome, Italy
roberto.ronchetti@uniroma1.it

### *Background*
It has long been known that a number of infective agents have the capacity to induce severe cerebral lesions in fetuses of women infected during the first months of pregnancy, but this was considered to be a rather rare event, under the influence of several environmental conditions. During the last few years, the Zika virus, known for decades as an agent of a very mild disease, has for the first time rapidly spread across Central and South America. Some months ago an international alarm ("Public Health Emergency of International Concern") was created under the hypothesis that this virus could be the cause of a large epidemic (thousands of cases) of cerebral malformations and microcephalia in Brazil. This WHO declaration has had enormous environmental and economic consequences worldwide.

*Methods*

We summarize the scientific literature (and the daily contributions of health authorities and of "experts" in the media reports) available on the issue of the Brazilian "epidemic" of microcephaly which could be caused by the Zika virus.

*Results*

According to available data, after one year from the beginning of the "epidemic", three things can be said:
1) in Brazil the number of cases of craniostenosis confirmed from September 2015 up to the present is not higher than the incidence expected on the basis of international global epidemiology.
2) in Brazil more than 80% of these cases of microcephalia were registered in a few states in the northeast of the country, where peaks of this malformation were also registered several years before the appearance of the Zika virus. At the same time, no cases were reported in the western or southern states of the country where Zika virus infection has the highest incidence.
3) in none of the more than 60 other countries around the world in which the Zika virus is epidemiologically active has there been a reported increase in the incidence of microcephalia.

*Conclusions*

These data suggest that in Brazil there is no link between the Zika virus and an "epidemic" of microcephalia. If this is the case, then the "scientific medical community" should discuss why, at least sometimes, an unjustified "burden" can arise from causes outside of a scientific rationale.

---

### Prenatal Early Exposure to the Environmental Endocrine Disruptors

**María Ángeles Martínez (1), Joaquim Rovira (1,2), Raju Prasad Sharma (1), Vikas Kumar (1), Martí Nadal (2), José L. Domingo (2), Marta Schuhmacher (1,2)**

**(1) Environmental Engineering Laboratory, Departament d'Enginyeria Quimica, Universitat Rovira i Virgili, Catalonia, Spain**
**(2) Laboratory of Toxicology and Environmental Health, School of Medicine, L'Institut d'Investigació Sanitària Pere Virgili, Universitat Rovira i Virgili, Catalonia, Spain**

**Corresponding author:**
**Rovira, Joaquim**
**Catalonia, Spain**
**joaquim.rovira@urv.cat**

*Background*

Endocrine disruptors (EDs) are chemicals compounds that send confusing messages causing various dysfunctions by mimicking or altering the effect of hormones in the human body. Several EDs such as bisphenol A, phthalates, organotins, among others are involved in obesity and diabetes diseases in children. Recent studies have shown evidences that these chemicals can cross the placental barrier making fetal exposure closely related to maternal exposure. The aim of this research is to establish and detect early fetal exposure to different EDs.

*Methods*

To address this issue, recently, a human biomonitoring campaign in a cohort of pregnant women has begun, in order to predict the ED mothers' intake and also the dose of ED at which the child has been exposed in the early stage of the development. The data obtained from this cohort (biological levels of EDs and results from questionnaires regarding food frequency intake, habits, and lifestyles) will be implemented into the physiological based pharmacokinetic model. For the physiological based pharmacokinetic model development and parameterization, data from literature were used.  Approval of the hospital ethical committee was received.

*Results*

Regarding the human biomonitoring campaign, complete data are expected to be analyzed by April 2018. Development of PBPK models and parameterisation for selected EDs are in progress. Initial validation of these models is performed using literature data. Biomonitoring data obtained during this study will be cross validated and further analysed to understand early fetal exposure of selected EDs.

*Conclusions*

The integration of the data obtained from current on-going human biomonitoring campaign and the physiological based pharmacokinetic model, here developed, will predict the prenatal early exposure of the child/foetus to EDs. This work is included in the frame of HEALS project (FP7-603946).

### Particulate Matter Characterisation in Schools Located around Petrochemical Industrial Complex: Infiltration of Particles From Outdoor to Indoor Air

Francisco Sanchez-Soberón (1), Ewelina Badowska (1), Joaquim Rovira (1,2), Montserrat Mari (1), Jordi Sierra (1,3), Vikas Kumar (1), José L. Domingo (2), Marta Schuhmacher (1,2)

(1) Environmental Engineering Laboratory, Departament d'Enginyeria Quimica, Universitat Rovira i Virgili, Catalonia, Spain
(2) Laboratory of Toxicology and Environmental Health, School of Medicine, L'Institut d'Investigació Sanitària Pere Virgili, Universitat Rovira i Virgili, Catalonia, Spain
(3) Laboratory of Soil Science, Faculty of Pharmacy, Universitat de Barcelona, Catalonia, Spain

Corresponding author:
Rovira, Joaquim
Catalonia, Spain
joaquim.rovira@urv.cat

*Background*

Evidence of associations between exposure to ambient particulate matter (PM) and cardiovascular and respiratory diseases has being widely reported. In recent years, in addition to $PM_{10}$ and $PM_{2.5}$ (particle having a diameter of less than 10 and 2.5 µm, respectively), other smaller fractions such as $PM_1$ and “ultrafine” particles ($PM_{0.1}$) become more relevant in the literature due to their capacity to deposit deeper in the respiratory tract. With a changing lifestyle, children, same as adults, spend most of their time in indoors (mainly in school and homes). However compared to adults, children are more susceptible to air pollutants. The aim of this study is to characterize the composition of indoor PM fractions at schools and validate with an indoor air quality model.

*Methods*

Indoor and outdoor samples of $PM_{10}$, $PM_{2.5}$, $PM_1$ and $PM_{0.25}$ were collected in different schools close to one of the largest industrial complexes of southern Europe located in Tarragona (Northeast Spain). Using AERMOD, an air dispersion model, schools were selected from the most impacted by industrial (chemical and petrochemical) and harbor emissions. PM fraction levels were measured in both the indoor and outdoor environment of schools using two different samplers, high volume samplers and cascade impactors for outdoor and indoor air, respectively. Chemical characterization was carried out by analyzing elements and ions content. In addition, electron microscope analyses were done in order to assess the morphology of different particles fractions. In order to establish the amount of particles coming from ambient air to inside the schools, an indoor air quality simulation model (IAQX 1.1) was applied. This model takes into account building parameters such as ventilation rates and room volumes in order to calculate the infiltration of outdoor particles to indoor compartments. Sensitivity analysis and calibration with real data were applied.

*Results*
Despite the absence of indoor PM sources, indoor PM levels were higher than outdoor. The gym was the environment where highest levels of PM were reached. These results are related with the phenomena of resuspension. For that reason, the lack of a module to introduce resuspension is the main limitation of indoor air quality simulation. As a result, modelled indoor PM levels were about 10% of actual levels. To correct this, resuspension was introduced into the model as an indoor source, having thus a higher similitude between modelled and measured PM levels. Chemical characterization of outdoor PM show a higher contribution from every component in $PM_{2.5}$ fraction, except for Secondary inorganic aerosols Regarding morphology, four main types of particles were found in our samples: fly ash, mineral, soot and undetermined.

*Conclusions*
Higher levels of PM were experienced in indoor environments, mainly by the effect of resuspension. IAQX model results are improved by taking into account the phenomena of resuspension as an indoor particle source. Characterization of outdoor PM shows that playgrounds could act as a source of fine mineral matter. Future research with more samples is needed to improve the present results. This work is in the framework of the Ultrapar project (Spanish Ministry) and HEALS project (7FP European Union).

---

### School Children Exposure to Particulate Matter (Coarse, Fine, and Quasi-Ultrafine) around Petrochemical Complex: Respiratory Tract Dosimetry

Francisco Sanchez-Soberón (1), Montserrat Mari (1), Vikas Kumar (1), José L. Domingo (2), Joaquim Rovira (1,2) Marta Schuhmacher (2)

(1) Environmental Engineering Laboratory, Departament d'Enginyeria Quimica, Universitat Rovira i Virgili, Av. Països Catalans 26, 43007 Tarragona, Catalonia, Spain
(2) Laboratory of Toxicology and Environmental Health, School of Medicine, L'Institut d'Investigació Sanitària Pere Virgili, Universitat Rovira i Virgili, Catalonia, Spain

Corresponding author:
Rovira, Joaquim
Catalonia, Spain
joaquim.rovira@urv.cat

*Background*
With the changing lifestyle in developed countries, children are spending more than 80% of their time indoors. Pollutants levels, as well as emission sources, could be different between inside and outside buildings and change across heterogeneous indoor environments. Children are one of the most vulnerable population groups to environmental pollutants, including particulate matter (PM). In a previous study we measured several fractions of indoor PM ("coarse" ($PM_{10}$-$PM_{2.5}$), "fine" ($PM_{2.5}$-$PM_{0.25}$) and "quasi-ultrafine" ($PM_{0.25}$)) in several schools. These schools are in a highly impacted area of the largest petrochemical industrial complex in southern Europe, located in Tarragona (Northeast Spain). The aim of the present study is to provide an advanced risk assessment of PMs in a vulnerable population cohort by: 1) characterizing the PM exposure of children in schools impacted by industrial emission in Tarragona; and 2) assessing of primary toxicity of these particle fractions through 3-(4,5-dimethylthiazol-2-yl)-2,5-diphenyltetrazolium Bromide assay with epithelial alveolar cell. Mechanistic exposure models can provide a valuable insight that considers human variability for risk assessment.

### *Methods*

The exposure model applied was a respiratory tract dosimetry model that evaluates the dose retained in the different parts of the human respiratory tract (Multiple-Path Particle Dosimetry Model). This exposure model takes into account the parameters affecting human exposure to particles such as PM concentration and size, and activity patterns of children in different environments (indoors and outdoors). The activity patterns of children were assessed using time use surveys and physical activity questionnaires. The 3-(4,5-dimethylthiazol-2-yl)-2,5-diphenyltetrazolium bromide assays with human pulmonary alveolar cells were conducted to evaluate the viability of exposed cells to PM collected in the schools.

### *Results*

A total mass of 198 µg was retained daily by children in the respiratory tract as a whole. More than half of this mass was retained in the head region. Maximum deposition fractions for every PM fraction were reached in this region. Similar mass of PM was contained in tracheobronchial (41.1 µg) and pulmonary (37.5 µg) regions. However, most of the mass found in the tracheobronchial region is composed of $PM_{10-2.5}$, while deposition in lungs consists almost exclusively of $PM_{2.5-0.25}$ and $PM_{0.25}$. Since most of the particles reaching the alveolar region were the fractions $PM_{0.25}$ and $PM_{2.5-0.25}$, we performed the cytotoxicity assay for these two fractions. At low doses, $PM_{0.25}$ show greater toxicity, while doses equal or higher than 50 µg/mL exhibit similar damage for both fractions.

### *Conclusions*

The approach here described provides a framework for evaluating of PM exposure and toxicology. Principal advantages of this methodology are the ability of working with different microenvironments, activities, and population collectives, instead of using only PM levels in one environment (usually outdoor) and physiological parameters of one population group (usually adult males). This methodology also provides a powerful tool to join toxicological assays with real PM exposures. This work is in the framework of the Ultrapar project (Spanish Ministry) and HEALS project (7FP European Union).

### Exposure of Children to Polycyclic Aromatic Hydrocarbons and Potential Carcinogenic Risk Assessment From Biomass Burning Incorporating Internal Dosimetry Metrics

Dimosthenis A. Sarigiannis (1,2), Dimitrios Zikopoulos (1), Spyridoula Nikolaki (1), Marianthi Kermenidou (1), Spyros Karakitsios (1)

(1) Environmental Engineering Laboratory, Chemical Engineering Department, Aristotle University of Thessaloniki, Thessaloniki, Greece
(2) Environmental Health Engineering, Scuola Universitaria Superiore IUSS, Pavia, Italy

Corresponding author:
Sarigiannis, Dimosthenis A.
Thessaloniki, Greece and Pavia, Italy
denis@eng.auth.gr
denis.sarigiannis@iusspavia.it

*Background*

The current study deals with the assessment of the children's cancer risk attributable to exposure to polycyclic aromatic hydrocarbons (PAHs), under an increased use of biomass for space heating in Greece since the winter of 2012-2013.

*Methods*

The study incorporated ambient air particulate matter (PM) sampling in several sites, as well as chemical analysis of PAHs and levoglucosan, as the most specific tracer of biomass combustion. External exposure was estimated accounting for PAHs concentration variability among the various locations and the use of personal sensors providing information on location, temperature and intensity of activity. Internal exposure to PAHs was estimated taking into account the deposition of the respective PM fractions across the human respiratory tract and the respective PAHs concentration of the respective PM fractions. Deposition at different regions of the human respiratory tract was estimated using the Multiple-Path Particle Dosimetry model. Potential cancer risk due to exposure to the mixture of urban ambient air PAHs was calculated using the toxicity equivalence factor approach using as basis the benzo(a)pyrene cancer potency. Cancer risk was estimated from the integral of the toxic equivalent quotient of the different size of PM deposited daily across different human respiratory tract regions, by a slope-factor equal to $0.25 \cdot 10^{-6}$ ng/kg body weight/day) function, initially derived by the benzo(a)pyrene Inhalation Unit Risk (equal to $0.88 \cdot 10^{-6}$ $(ng/m^3)^{-1}$).

This refined exposure and risk characterization methodology allowed us to identify significant differences experienced by the different age group, as well as people living in different areas within an urban agglomeration.

*Results*

The human respiratory tract deposition results indicated that the lower respiratory tract of infants and children (up to 14 years old) can retain up to 74% higher mass fraction of $PM_1$ particles than that of adults. The maximum difference in the thoracic deposition between adults and children (referring to children between 3 and 8 years old) is that of 68% and 230% for the $PM_{2.5-1}$ and $PM_{10-2.5}$ fractions respectively. Thus, the $PM_{2.5}$ and $PM_1$ fractions rather than $PM_{10}$ contribute to a greater extent to the absorption of PAHs by the respiratory tract in younger individuals compared to adults. The estimated lung cancer risk was above 10-6 for the areas affected by biomass combustion. Age dependent differences in the estimated risk were mainly attributed to the respiratory physiology differences and the increased intensity of activity characterizing children, favoring the deposition of smaller (and more toxic particles) in children. In addition, biomass emitted particles were found to be more toxic (in terms of PAHs content) than the ones emitted from traffic sources.

### *Conclusions*

Age dependent differences in human physiology, combined with the increased intensity of activity characterizing children, resulted in 2-3 times higher risk estimates from exposure to PAHs compared to adults.

### *Acknowledgements*

This work was financially supported partially by the European Commission through the LIFE+ project CROME-LIFE.

---

### Internal Dosimetry Metrics for Risk Assessment of Endocrine Disruptors — The Case of Bisphenol A

**Dimosthenis A. Sarigiannis (1,2), Spyros Karakitsios (1), Evangelos Handakas (1), Alberto Gotti (1)**

**(1) Environmental Engineering Laboratory, Chemical Engineering Department, Aristotle University of Thessaloniki, Thessaloniki, Greece**
**(2) Environmental Health Engineering, Istituto Universitario di Studi Superiori di Pavia, Pavia, Italy**

**Corresponding author:**
**Sarigiannis, Dimosthenis A.**
**Thessaloniki, Greece and Pavia, Italy**
**denis@eng.auth.gr**
**denis.sarigiannis@iusspavia.it**

### *Background*

Bisphenol A (BPA) remains one of the most controversial industrial chemicals with respect to early developmental stage exposure and effects.

### *Methods*

The current study aims at a comprehensive exposure analysis of BPA, using an integrated exposure modelling framework that comprises far field and near field exposure modelling coupled to a dynamic lifetime physiologically based pharmacokinetic model. Exposure analysis was applied in European data, largely based on literature review of BPA food residues, as well as exposure reconstruction of human biomonitoring data. The latter were further assimilated through an advanced exposure reconstruction modelling framework. Special attention was paid to the assessment of BPA internal exposure through critical developmental stages such as gestation, by modelling the mother-fetus toxicokinetic interaction.

*Results*

The analysis indicated that current exposure levels in Europe are below the temporary tolerable daily intake of 4 µg/kg body weight/day proposed by the European Food Safety Authority. For chemicals with widespread consumer applications such as BPA, environmental pathways contribute insignificantly to BPA intake, while overall exposure is dominated by specific consumer behaviors. For BPA these pertain to consumption of canned food and beverages and the use of medical products found in neonatal intensive care units, such as bags containing intravenous fluids and total parenteral nutrition and tubing associated with their administration. Premature neonates hosted in intensive care units have also been also identified as the only population group that potentially faces some risk related to BPA exposure, especially when accounting for the immaturity of the detoxification pathway. In principle, health risks might be underestimated for specific population groups (e.g. neonates and infants) if the assessment does not take into account the variability in internal exposure due to genetic, physiological and developmental factors. In utero exposure was found to be highly associated to maternal exposure; actual fetal exposure was higher than maternal exposure by almost 20% due to the presence of β–glucuronidase in the placenta. Nevertheless, at the current levels of maternal exposure to BPA, in utero exposure is also very low, and significantly below the respective biologically effective dose derived from the European Food Safety Authority temporary tolerable daily intake.

Exposure reconstruction of literature available on human biomonitoring data resulted in very low intake estimates, similar to the ones estimated from the exposure models. Using biological pathway altering dose derived from in vitro BPA toxicity assessment as the internal exposure reference value, the maximum derived internal exposure values of the worst-case exposure scenarios (premature neonates) are 10 times lower to the lower 99th percentile of the Biological Pathway Activating dose, indicating that there is no reason for concern for individual or aggregate scenarios of BPA exposure.

*Conclusions*

In addition, the use of the internal dosimetry module allowed the translation of the European Food Safety Authority temporary tolerable daily intake into a biomonitoring equivalent urinary BPA-Glu concentration of320 µg/L. Comparison of this value to the collected biomonitoring data shows that the current existing levels of BPA in EU are 2 orders of magnitude below the European Food Safety Authority temporary tolerable daily intake; this margin of safety is in the same magnitude of order to the one estimated when daily intake is estimated from human biomonitoring data.

### Assessing the Impact of Hazardous Waste on Children's Health: The Exposome Paradigm

Dimosthenis A. Sarigiannis (1,2)

(1) Environmental Engineering Laboratory, Chemical Engineering Department, Aristotle University of Thessaloniki, Thessaloniki, Greece
(2) Environmental Health Engineering, Scuola Universitaria Superiore IUSS Pavia, Pavia, Italy

Corresponding author:
Sarigiannis, Dimosthenis A.
Thessaloniki, Greece and Pavia, Italy
denis@eng.auth.gr
denis.sarigiannis@iusspavia.it

*Background*

Assessment of the health impacts (e.g. cancer, neurodevelopmental disorders and congenital anomalies) related to hazardous waste is an emerging scientific challenge with multiple societal implications. Most studies on the links between hazardous waste and health are based on proxies of exposure to waste-related toxicants. They do not provide established mechanistic links between environmental exposure and disease burden prohibiting the exploration of cost-effective waste management options.

*Methods*

The exposome paradigm embraces dynamic interactions between environmental and endogenous exposures and genetic expression in humans, with a special focus on early developmental stages that have been proven determining for later life health status. Thus, it provides a novel tool for holistic hazardous waste management taking into account their adverse effects on children's health. Waste streams are associated with a broad range of contaminants, such as PCDDs and PCDFs, toxic metals (Cd, As, Cr(VI), Hg), VOCs, PAHs, $H_2S$, particulate matter, CH4, pesticides, phthalates, and PCBs. All of these compounds form a complex mixture of highly variable composition in space and time—accounting for differences in multimedia environmental transfer, persistence and bioaccumulation among the mixture components—to which children are exposed through multiple pathways and routes. Waste streams and the related contamination of environmental media are not viewed in isolation, but rather as components of the expotype, the vector of exposures an individual is exposed to over time.

An array of sensors including in situ systems for regulatory monitoring of environmental media, remote and personal sensors and human biomonitoring were used to provide the data necessary for determining the external exposome associated with hazardous waste contamination. Advanced data and model fusion algorithms were designed to integrate functionally integrate these different environmental datasets taking into account and adequately filling data gaps in space and time. External exposures were translated into internal dose through the use of physiology-based biokinetic models and they were assimilated with the biomonitoring data. Biological perturbations were identified through the use of multi-omics technologies. According to our paradigm, starting from untargeted transcriptomics and metabolomics we proceed with joint analysis of biological processes induced by exposure to waste-related xenobiotics at the molecular level and of metabolic processes induced in parallel. Dynamic flux balance analysis is used to jointly interpret gene expression data and metabolite profiles. Thus putative pathways of toxicity are identified; they need to be verified by targeted multi-omics and functional assays to confirm the causal association of waste components with adverse health outcomes. Advanced bioinformatics deep learning algorithms allow us to identify the functional links among the data derived from high throughput testing platforms and disease phenotypes providing phenotypic anchoring of the mechanistic hypotheses made earlier. Waste management practices are thus assessed with regard to their effects on children's health at the individual and community level, taking into account intra-subject variability.

### Results

The health risk associated with exposure to potential contaminants from recycling waste streams and recycled waste processing under normal and accidental conditions was assessed. Experimental data were obtained for the case of a disastrous fire at the waste recycling facility in Aspropyrgos, Greece. Based on agnostic metabolome analysis, inhibition of de novo fatty acid biosynthesis was manifested by down-regulation of acetyl-CoA, malonyl-CoA, palmitoyl-CoA metabolites and related mRNA levels. Results strongly support the important role of the AhR in mediating the metabolic effects seen following PCDDs/PCDFs exposure. This additional evidence on the AhR perturbation, allowed the use of the well-established slope factor for TCDDs and to translate this into an internal exposure slope factor. The latter was used to estimate risk for the exposed and the non-exposed population. The risk of chronic exposure was estimated (mean value) equal to $2.57 \cdot 10^{-7}$, while the exposed population risk (mean value) was up to $2.91 \cdot 10^{-7}$. The respective risk is expected to be higher for neonates (almost 20%) and even higher for neonates that are also breast fed (up to 32%).

### Conclusions

There is sufficient evidence that the various waste management options contribute to potential health effects; yet it is obvious that a more systematic approach is required. Exposome research proposes a paradigm shift that goes far beyond the proxy of exposure and macroscopic health effects association, enhancing our understanding of (a) the type of compounds released from the waste management sites, (b) the environmental fate of these compounds, (c) how population space-time lines cross with the space-time lines of these compounds and (d) the effects at the multiple biological organization levels as a result of this cross.

### School Environmental Health: the First Steps Towards Environmental Health

Miguel Felipe Sánchez-Sauco (3,4), Mayra Alejandra Orozco Llamas (1,2,3), Juan Miguel López Verdú (4), Almudena Pernas Barahona (3,4), Alberto Cárceles-Álvarez (3,4), María Luisa Azurmendi Funes (3,4), Esther Tobarra Sánchez (3,4), Lizbeth Álvarez Gómez (1,3,4), Juan Antonio Ortega-García (3,4)

(1) Mount Sinai School of Medicine, Pediatric Environmental Health Speciality Unit, New York, USA
(2) University Hospital Virgen de la Arrixaca, Murcia, Spain
(3) Laboratory of Environment and Human Health, Instituto Murciano de Investigación Biosanitaria, Murcia, Spain
(4) Pediatric Environmental Health Speciality Unit, University Hospital Virgen de la Arrixaca, Murcia, Spain

Corresponding author:
Sauco, Miguel Felipe Sánchez
Murcia, Spain
miguel@pehsu.org

*Background*

The educational stage (3-18 years) is a period of life in which environmental factors have a major impact on a child. Environmental factors shape current and future adulthood biological strengths and weaknesses. In addition, during the educational stage social and emotional interactions form personality.

*Methods*

Since 2006, the Pediatric Environmental Health Specialty Unit of the Hospital Clínico Universitario Virgen de la Arrixaca has worked along with Colegio San Jorge (Murcia) to promote school environmental health by developing knowledge and maintained goals/concerns that allow us to carry out and implement concrete activities in schools.

The first step was to have the approval and commitment of the school administration to free a professor for a couple hours a month to carry out these tasks. Subsequently, a complete environmental diagnosis of the school was completed in order to understand the strengths and weaknesses of the environment of the school. Once completed, the goals were developed, two of which were implemented and one which will be implemented next year.

*Results*

1st Goal – Tobacco: Identification of the prevalence of smoking at school, awareness activities, ephemeris celebrations (global day without tobacco), and group therapies of tobacco dishabituation for professors and parents of students.

2nd Goal – Ultraviolet Radiation: Including a hat for the school uniform, choosing a time during the year least hot for the most exhaustive physical activity, as well as organizing schedules so that the younger children have exposure to the sun earlier in the day.

3rd Goal – Pesticides: Inform parents at school of fumigation days and the products that are used, production and use of organic pesticides, awareness activities.

### *Conclusions*

These goals are integrated into the day-to-day activities of the school, which affects future habits and strengths of both students and the school community.

### Smoking Cessation Consultation In Pediatrics

**Miguel Felipe Sánchez-Sauco (3,4), Lizbeth Álvarez Gómez (1,3,4), Alberto Cárceles-Álvarez (3,4), Almudena Pernas Barahona (3, 4), Esther Tobarra Sánchez (3,4), María Luisa Azurmendi Funes (3,4), Mayra Alejandra Orozco Llamas (1,2,3), Juan Antonio Ortega-García (3,4)**

**(1) Mount Sinai School of Medicine, Pediatric Environmental Health Speciality Unit, New York, USA**
**(2) University Hospital Virgen de la Arrixaca, Murcia, Spain**
**(3) Laboratory of Environment and Human Health, Instituto Murciano de Investigación Biosanitaria, Murcia, Spain**
**(4) Pediatric Environmental Health Speciality Unit, University Hospital Virgen de la Arrixaca, Murcia, Spain**

**Corresponding author:**
**Sauco, Miguel Felipe Sánchez**
**Murcia, Spain**
**miguel@pehsu.org**

### *Background*

Exposure to tobacco smoke (active or passive) is one of the leading causes of pregnancy complications and a risk factor for developing multiple pediatric diseases. Smoking cessation consultation in pediatrics is aimed at pregnant couples. The consult has a group structure (although in exceptional cases is done individually) and is divided into 5 in-person and 5 telephone sessions. The main action developed in the consult is the control of nicotine dependence, and above all, behavioral and emotional dependence to tobacco.

### *Methods*

During 2016, we developed 3 therapy groups with a mean of 5 patients per group. Individuals who did not attend all sessions and also did not respond to the telephone calls were excluded from the results. There were a total of 16 patients, 12 women and 4 men. The average consumption of cigarettes/day average was 9.78.

### *Results*

For the Fagerström test there was an average of 4.4 (little dependence) and for the Richmond test there was an average of 7.5 (moderate motivation). The current cessation rate is 75%. Emotional and behavioral control is very important during pregnancy. The inclusion of the partner in the process ensures that the cessation is long-lasting, while also promoting a smoke free childhood environment.

### *Conclusions*

The strengths of group therapy are that individuals with the same characteristics (feelings, fears, motivations, etc.), avoid feeling lonely during the process. The group can solve doubts and questions (fears, shyness, etc.) that otherwise would not be resolved.  It helps us to establish pertinent emotional linkages among patients within the group. The results indicate a strong adherence to treatment.

---

### Environmental Reproductive Nursing Consultation: Health from the Beginning

Miguel Felipe Sánchez-Sauco (1,3), Almudena Pernas Barahona (1,3), Ferran Campillo i López (1,3), Alberto Cárceles-Álvarez (1,3), María Luisa Azurmendi Funes (1,3), Esther Tobarra Sánchez (1,3), Mayra Alejandra Orozco Llamas (2,3), Lizbeth Álvarez Gómez (2,3), Juan Antonio Ortega-García (1,3)

(1) Pediatric Environmental Health Speciality Unit, University Hospital Virgen de la Arrixaca, Murcia, Spain
(2) Mount Sinai School of Medicine, New York, USA
(3) Laboratory of Environment and Human Health, Instituto Murciano de Investigación Biosanitaria, Murcia, Spain

Corresponding author:
Sauco, Miguel Felipe Sánchez
Murcia, Spain
miguel@pehsu.org

### *Background*

The Environmental Reproductive Nursing consultation involves a clinical consultation focused on detection, intervention and prevention of exposures to environmental hazards during the critical periods of fetus development (spermatogenesis and pregnancy) that pays special attention to legal and illegal drugs.

### *Methods*

The consultation is supplemented with an ultrasound at 12 weeks of gestation. The goals of the consultation are to detect and reduce environmental exposures that can negatively affect the health of the fetus (or embryo) and to prevent recurrent diseases. In addition, to establish “environmental risk labels “ in those pregnancies where the exposures have been severe enough to trigger diseases related to those exposures and allow us to make diagnoses prenatally or in the first months of life. The working tool is called the “hoja verde” and consists of a set of basic and concise questions that allow us to identify and manage/reduce environmental risk factors at critical periods of pregnancy (including spermatogenesis) and breastfeeding. We examine socioeconomic factors, reproductive history, ionizing radiation, drugs/herbalism/homeopathy, occupational exposures, smoking, illegal drugs, alcohol, pesticides and environmental perception of their community. Ethical approval was granted by La Hoja Verde’s ethics committee.

*Results*

Of the first 1,500 couples with risk during early pregnancy (2009/2012), we found a rate of 34.9% of maternal smoking, 45.4% of paternal smoking, 59.4% of maternal alcohol consumption during early pregnancy, 85% of paternal alcohol consumption during spermatogenesis, and 10.1% of fetuses exposed to illegal drugs during early pregnancy. In the successive years, the data presented showed a significant decrease in women who drink some alcohol in early pregnancy, a significant decrease in the amount of alcohol that the couples consume and a decrease in the number of newborns with congenital heart disease associated with intrauterine exposure to ethanol.

*Conclusions*

The Environmental Reproductive Nursing consultation is a clinical consultation/practice that allows us to identify, address and prevent environmental risk factors that would be otherwise go unnoticed. The training in this type of discipline/consultation is critical in order to increase the quality of pregnancy, health of the pregnant couple, as well as the health of the upcoming newborn.

---

### Blood Lead Concentrations From Children Practicing Airgun Shooting

Alexandra Grabmann (1), Rudolf Schierl (1), Hermann Fromme (1,2), Thomas Göen (3), Dennis Nowak (1)

(1) Institute for Occupational, Social and Environmental Medicine, Hospital of University Munich, Munich, Germany
(2) Bavarian Health and Food Safety Authority, Department of Chemical Safety and Toxicology Munich, Germany
(3) Institute for Occupational, Social and Environmental Medicine, University of Erlangen, Germany

Corresponding author:
Schierl, Rudolf
Munich, Germany
Rudolf.Schierl@med.uni-muenchen.de

*Background*

Elevated lead concentrations in blood samples from adult indoor shooters are well known. Children are shooting mainly with airguns, but data about blood lead concentrations is missing. Such data would be important to know because even lead concentrations from children below 100 µg/l are under suspect nowadays.

*Methods*

For this investigation, children between the ages of 12 and 16 years were recruited after agreement of the local ethical committee. Blood samples (1.2 ml ethylenediaminetetraacetic acid) were drawn from 54 shooters (67% boys) and 48 controls (79% boys) after written consent from children and parents. Lead was analyzed by graphite furnace atomic absorption spectroscopy (GF-AAS) under strict internal and external quality control. All 102 samples were above the detection limit of 5.0 µg/l. Ethical approval was granted by the Hospital of University Munich, Munich, Germany.

### *Results*

The shooters reported a mean training time of 4.8 (SD 2.7) hours per month with 215 shots (SD 115). The mean lead concentration of shooters was 32.4 µg/l (SD 15.1) and somewhat higher compared to controls (mean 25.4 µg/l, SD 13.2). The difference was borderline significant ($p = 0.048$). There was no difference between results from boys and girls and there was no correlation between shooting hours and blood lead levels.

### *Conclusions*

The results underline that shooting with airguns maybe an additional source of lead in children. Due to the known health effects of lead for children, all sources of lead uptake need to be reduced. Further research is necessary to obtain more recent data of the body burden of children in Germany and to look to previously unknown sources.

---

### Training Modules for Pediatricians: Health Effects of Climate Change

**Julia Schoierer (1), Colin O'Reilly (1), Stephan Bose-O'Reilly (1, 2)**

**(1) Unit Pediatric Environmental Epidemiology, Institute and Outpatient Clinic for Occupational, Social and Environmental Medicine, University Hospital Munich, Munich, Germany**
**(2) Institute of Public Health, Medical Decision Making and Health Technology Assessment, Department of Public Health, Health Services Research and Health Technology Assessment, University for Health Sciences, Medical Informatics and Technology), Hall in Tirol, Austria**

**Corresponding author:**
**Schoierer, Julia**
**Munich, Germany**
**Julia.Schoierer@med.uni-muenchen.de**

### *Background*

Health care systems will be increasingly faced with new challenges due to climate change-related health risks. There are several risks to be mentioned: heat-related health problems, allergies and respiratory diseases as well as a new onset of infectious diseases, increased risk of skin cancer due to strong ultraviolet radiation and accidents due to extreme weather events and as a result - mental stress. Children, due to their vulnerability, are seen as a special risk group for numerous pollution and exposure to climate change related factors. It is therefore important for the health sector to improve knowledge about the health effects of climate change.

### *Methods*

As part of the German Strategy for Adaptation to Climate Change (DAS), the Institute of Occupational, Social and Environmental Medicine at the Ludwig Maximilian University of Munich receives funding for a project from the Federal Ministry for Environment, Nature Conservation, Building and Nuclear Safety (grant number 03DAS073) to develop training modules for pediatricians.

### Results

The project funding was launched in April 2016 and will continue until March of 2019. With the support of a cooperation network of professional associations and stakeholders of the target group, a blended learning module is currently being developed for pediatricians. The e-learning phase will begin in spring 2017. The phase to phase teaching will take place during three pediatric annual meetings from summer to autumn 2017.

### Conclusions

The project aims to provide scientific knowledge on climate change-related health risks for children and adolescents. Pediatricians will develop necessary skills for the new challenges in diagnosis, therapy and prevention. It is hoped that the project will enable adequate preventive and curative action for children.

### Thinking of the Relationship Between Children's Health and the Environment as a Rights Issue — the UN Committee on the Rights of the Child Day: General Discussion on Children's Rights and the Environment on 23 September 2016

Jonas Schubert

Terre des Hommes, Osnabrück, Germany

Corresponding author:
Schubert, Jonas
Osnabrück, Germany
j.schubert@tdh.de

### Background

In recent years, it has become increasingly clear that environmental harm is one of the most pressing human rights challenges facing humanity in the twenty-first century. A vast range of human rights, including the rights to life, health, food, water, and housing, depend on a healthy environment. Children are particularly vulnerable to environmental harm. Nevertheless, the effect of environmental degradation on their lives is rarely addressed as a rights issue. The UN Convention on the Rights of the Child (CRC) is often overlooked in setting and implementing environmental policies and standards even though the convention specifically identifies "the dangers and risks of environmental pollution" as a threat to the right to health. States rarely address environmental issues in their periodic reports to the Committee on the Rights of the Child, which monitors the implementation of the CRC, and while many NGOs (and academics) have started working on human rights and the environment, only a few of them cover issues related to children in particular.

*Methods*
The Committee on the Rights of the Child (CRC) periodically devotes one day of general discussion (DGD) to a specific article of the UN Convention on the Rights of the Child (CRC) or to a child rights issue. The purpose of the DGD is to foster a deeper understanding of the contents and implications of the UN Convention. The 2016 Day of General Discussion (DGD) on 23 September will be on the topic of children's rights and the environment. Local and global experts from different fields will participate.

*Results*
The presentation will provide an overview of the main objectives and scope of the UN conference and highlight key aspects of the current debate, including the potential added value of a rights—based approach to children's environmental health.

*Conclusions*
To strengthen the link between the protection of children's environmental health and rights, the child rights community should aim to clarify how relevant provisions of the UN Convention on the Rights of the Child relate to environmental issues. Environmental decision-makers, on the other hand, should explicitly recognize that children's rights apply to environmental (health) matters. Furthermore, environmental health policy-makers and experts should become more familiar with the specifics of the UN Convention on the Rights of the Child and learn how to apply it to their field. Human rights experts would hugely benefit from tailor-made data and arguments to frame environmental health issues in human rights terms.

---

### Mismatch of Classroom Furniture and Student Body Dimensions in Schools in Delhi, India

**Meghna Sharma, Renu Arora**

**Department of Resource Management, Institute of Home Economics, University of Delhi, New Delhi, India**

**Corresponding author:**
**Sharma, Meghna**
**New Delhi, India**
**meghnasharma77@yahoo.co.in**

*Background*
Children (aged 10-19) in India represent about 20% adolescents as proportion of total population. The classroom is a formal environment for learning. A conducive and comfortable classroom environment motivates students to perform better and encourages the learning process. Furniture plays an important role in the maintenance of good sitting posture. Using furniture that promotes proper posture is more important as sitting habits acquired in childhood are very difficult to change later in adolescence or adulthood. Anthropometric measurements are an important factor that should be taken into account in classroom furniture design. The function of the furniture should be to facilitate learning through providing a comfortable and stress free workstation. Hence, the classroom furniture needs to be designed to allow children to move about in their seats, as it is unnatural to keep still for long periods.

*Methods*
To fulfill this objective, an observation based study was undertaken in public schools of New Delhi for devising suitable furniture measurements for a sample size of 450 school children in relation to their anthropometric measurements. In the study, the school furniture measurements were compared with the Bureau of Indian Standards given for children of 10-13 years and > 13 yrs age group, but no comparison was made for the anthropometric measurements for children. However, a correlation between the anthropometric measurements with age was found, along with comparing the findings between the boys and girls.

*Results*
The results showed that none of the schools conformed to the standards given by the Bureau of Indian Standards for school furniture. Also, the majority of the students were getting injured because of certain defects in the classroom furniture. It was reported by 49.5% of school children that the classroom seat on which they were sitting for continuous durations was not comfortable to them. Also, 34.5% of school children from 10-11 years found the classroom seat to be uncomfortable to them, whereas 31.4% children from 12-13 years and 34.1% from the >13 years age group reported the same. Furthermore, the furniture dimensions are one of the main aspects and, amongst these, the seat height is typically the starting point for the design of this type of furniture. The study also indicated the potential relationship between anthropometric data of children and the furniture dimensions. Furthermore, we reported, based on the rules adopted from Parcells et al. (1999), the children's anthropometric measurements and furniture dimensions were mismatched.

*Conclusions*
This highlights the fact that students in the sample schools did not find the existing seats and desks to adequately fit their body dimensions, which indicates an urgent problem for school children in Delhi.

### Assessment of Indoor Air Quality in Air -Conditioned and Naturally Ventilated Schools in Delhi, India

Pratima Singh (1), Renu Arora (2)

(1) Department of Resource Management, University of Delhi, New Delhi, India
(2) Institute of Home Economics, New Delhi, India

Corresponding author:
Singh, Pratima
Delhi, India
pratima_lic@hotmail.com

*Background*
Understanding the impact of the indoor environment on children is important as they spend most of their time indoors (approximately 80-90%), either at school or the home. Within a school, students spend most of their time inside classrooms, engaging in various activities which require considerable amount of concentration and attention. Several research studies have indicated that the air quality inside classrooms is often poor and carbon dioxide ($CO_2$) concentration exceeds recommended levels prescribed by the American Society of Heating, Refrigerating and Air-Conditioning Engineers for indoor environments in majority of schools; which might be due to high student density or poor facilities at schools. If the air inside classrooms is deteriorated, it may cause several health effects that may directly impair concentration or memory of students – e.g., neurological effects – or cause other health effects that indirectly affect learning of children – e.g., absenteeism, poor concentration, restlessness or dizziness. Hence the present research was designed to assess the quality of air inside air-conditioned (AC) and naturally ventilated (NV) schools in Delhi, India.

*Methods*

The present research was exploratory in nature. The research was conducted in four private schools (two AC and two NV) located in Delhi, India. The design features of the selected classrooms were studied using a checklist and observation tool. The indoor-air quality parameters namely $CO_2$, temperature and relative humidity (rH) were monitored at continuous 5 minutes interval from 7:30 am to 2:00 pm for three working days and 8:00 am to 2:00 pm for one non-working day in each school using air quality monitor Testo 435-2.

*Results*

The results of the research study indicated that the measured $CO_2$ concentrations varied among school classrooms based on type of ventilation, windows, room occupancy and student movement. The average $CO_2$ concentration in naturally ventilated classrooms were within recommended limits (472.97 ppm to 718.02 ppm) on all the working day,s i.e less than 1000-ppm $CO_2$ limit prescribed by the American Society of Heating, Refrigerating and Air-Conditioning Engineers 62-2001 standards. However, the average $CO_2$ concentrations frequently exceeded the limits in the AC classrooms on all working days (1674.51 ppm to 3298.12 ppm). The $CO_2$ concentrations were within limits in all classrooms irrespective of ventilation type on all non-working days (minimum 386.08 ppm and maximum 478.37 ppm). The average temperature and rH ranged between 24.22˚C to 26.29˚C and 53.68% to 63.72%, respectively, in AC classrooms on working days providing adequate thermal comfort to occupants. The same was found to be ranging from 30.6˚C to 33.01˚C and 66.5% and 82.92% respectively in NV schools; exceeding the limits of comfortable temperature (approx. 20˚C to 28˚C) and rH ( less than 65%) suggested by the American Society of Heating, Refrigerating and Air-Conditioning Engineers. The analysis of the data highlighted the degraded indoor air quality in the air-conditioned school on all the working days.

*Conclusions*

The elevated indoor carbon dioxide concentrations in AC schools may indicate inadequate ventilation per occupant, which may lead to sick building syndrome symptoms in students affecting their health and performance in schools. On the contrary, students in NV schools often experienced thermal discomfort due to higher temperature and relative humidity in class.

### Quantitative Profiling of Metabolic Markers to Assess Dietary and Microbiota Related Exposure Factors

Zdenek Spacil (1), Tereza Pavlova (2), Jana Klánová (1)

(1) Research Centre for Toxic Compounds in the Environment, Masaryk University, Brno, Czech Republic
(2) Department of Pathological Physiology, Masaryk University, Brno, Czech Republic

Corresponding author:
Spacil, Zdenek
Brno, Czech Republic
spacil@recetox.muni.cz

*Background*

Dietary and intestinal microbiota related exposure factors directly influence human metabolism, thus affecting health states. Quantitative metabolic profiling by mass spectrometry and specifically selected reaction monitoring (SRM) assays may be used to capture an individual's metabolic phenotype, revealing potential perturbations. Biomarker levels correspond to a specific exposure factor and perhaps are consistent with early indications of disease. This study focuses on biochemical intermediates of tryptophan metabolism to explore the influence of dietary and microbial exposure factors on this essential biochemical pathway.

*Methods*

A chemical library containing 234 standards of diverse biochemical intermediates was analyzed using ultra-high pressure liquid chromatography- mass spectrometry system (1290 Infinity II and 6495 QqQ, both from Agilent, CA, USA) to develop SRM assays for all detectable metabolites. Urine samples (n=51) from pregnant women collected in the 38th week of gestation were lyophilized, extracted with methanol, internally standardized and assayed for a panel of metabolites relevant to tryptophan metabolism by SRM technology. Ethical approval was granted by the Ethics Committee of Central European Longitudinal Study of Pregnancy and Childhood.

*Results*

The dietary intake of tryptophan is essential in humans to maintain protein biosynthesis and synthesis of neurotransmitters (serotonin and melatonin). At the same time, tryptophan is metabolized by microbiota residing in the human intestine. The collective dietary and microbiota exposure was explored using highly selective, sensitive and reproducible SRM assays for 20 key intermediates of tryptophan metabolism. The levels of these intermediates were determined in urine samples from pregnant women collected in the frames of Central European Longitudinal Study for Pregnancy and Childhood in the region of South Moravia, Czech Republic. Quantitative metabotypes were compared in urine samples based on abundance levels and mutual ratios between metabolic markers. This study contributes novel data in the area of health related dietary and microbial exposure factors, particularly to the emerging field of the exposome (i.e. non genetic factors affecting human health).

### Conclusions

Innovative study of health related dietary and microbial exposure factors in the emerging field of the exposome.

### Impact of Air Pollution to Oxidative Damage In Newborns and Mothers

Radim J. Sram (1), Pavel Rössner, Jr (1), Andrea Rossnerova (1), Antonín Ambroz (1), Alena Milcova (1), Vlasta Svecova (1), Jana Pulkrabova (2), Jana Hajslova (2), Milos Veleminsky, Jr (3)

(1) Institute of Experimental Medicine AS CR, Prague, Czech Republic
(2) University of Chemistry and Technology, Prague, Czech Republic
(3) University of South Bohemia, Ceske Budejovice, Czech Republic

Corresponding author:
Sram, Radim J.
Prague, Czech Republic
sram@biomed.cas.cz

### Background

The aim of our study was to investigate the impact of air pollution on oxidative DNA damage and lipid peroxidation in the urine and blood from mothers and newborns from two localities with different levels of air pollution: Ceske Budejovice (CB), a locality with a clean air, and Karvina, a locality with high air pollution.

### Methods

The samples from normal deliveries (38-41 weeks+) of nonsmoking mothers and their newborns were collected in the summer and winter seasons. Levels of 8-oxo-7,8-dihydro-2'-deoxyguanosine (8-oxodG) in urine were analyzed with ELISA, levels of 15-F2t-isoprostane (15-F2t-IsoP) in blood plasma were analyzed with immunoassay kits. Ethical approval was granted by the Ethical Committee of the Institute of Experimental Medicine, Academy of Sciences of the Czech Republic, the Polyclinic Hospital Karvina-Raj, and by the Hospital Czech Budejovice.

### Results

Higher $PM_{2.5}$ (particulate matter < 2.5 mm) concentrations were found in Karvina than in CB in the summer of 2013, and in the winter of 2014 ($P < 0.001$). We observed significant differences in 15-F2t-IsoP levels between the summer and winter seasons in Karvina for newborns (mean±SD: 64.24 ± 26.75 vs. 104.26 ± 38.18 pg/ml plasma, respectively ($P < 0.001$). Levels of 8-oxodG differed only in the winter season between localities; they were significantly higher ($P < 0.001$) in newborns from Karvina in comparison with CB (mean±SD: 5.70 ± 2.94 vs. 4.23 ± 1.51 nmol/mmol creatinine, respectively).

### Conclusions

The results of multivariate regression analysis in newborns from Karvina showed $PM_{2.5}$ concentrations to be a significant predictor for 8-oxodG excretion, and $PM_{2.5}$ and benzo[a]pyrene concentrations to be a significant predictor for 15-F2t-IsoP levels. The results of multivariate regression analysis in mothers showed $PM_{2.5}$ concentrations to be a significant predictor of 8-oxodG levels.

Supported by the Czech Grant Agency13-13458S.

### Children's Environmental Burden of Disease: Review of Key Findings and General Methodology

Nadine Steckling (1,2), Myriam Tobollik (3), Stephan Bose-O'Reilly (1,2)

(1) University Hospital Munich, WHO Collaborating Centre for Occupational Health, Institute and Outpatient Clinic for Occupational, Social and Environmental Medicine, Workgroup Pediatric Environmental Epidemiology, Munich, Germany
(2) Institute of Public Health, Medical Decision Making and Health Technology Assessment, Department of Public Health, Health Services Research and Health Technology Assessment, University for Health Sciences, Medical Informatics and Technology, Eduard Wallnoefer Center I, Hall in Tirol, Austria
(3) German Environment Agency, Section Exposure Assessment and Environmental Health Indicators, Berlin, Germany

Corresponding author:
Steckling, Nadine
Munich, Germany
Nadine.Steckling@med.uni-muenchen.de

### *Background*
Children are vulnerable to environmental exposures resulting in disease burden. This can be quantified using the environmental burden of disease (EBD) method. EBD method and key findings of children's EBD are presented.

### *Methods*
Quantifying the EBD in terms of disability-adjusted life years (DALYs) is explained. Recent global studies estimating DALYs caused by environmental risk factors (ERF) for children are summarized and discussed to understand DALYs. A focus is given to global estimates from the World Health Organization (WHO) and the Institute of Health Metrics and Evaluation (IHME).

### *Results*
EBD is a method to quantify how many DALYs in a population are lost due to health restricted life time and premature death attributable to ERF. The WHO estimated that 25% of global DALYs for children (<5 years) are caused by modifiable ERF (e.g., air pollution, noise, unsafe water). In children, ERF contributes the most to the burden of infectious diseases, neonatal conditions, nutritional diseases, and injuries. E.g., 11% of 25 Mio DALYs due to neonatal conditions in children (<5 years) worldwide are assumed to be caused by ERF as in indoor/outdoor air pollution, tobacco smoke during pregnancy, unsafe water/

sanitation. In comparison, the environmental contribution to lower respiratory infections is with 60% of 52 Mio DALYs much higher. IHME quantified DALYs for a selection of ERF, including unsafe water/sanitation/handwashing, air pollution, radon, and lead. These ERF explain less than 5% of the total DALYs for children <5 years but more than 10% for children aged 5-9 years.

### *Conclusions*

The EBD studies differ regarding the definition of “environment” and inclusion of ERF. Moreover, data restrictions avoid the inclusion of all ERF of relevance. However, obvious is a high impact of the environment for children’s health. This contains a high chance for prevention of the burden by reducing the exposure.

This work has received funding from the European Union’s Seventh Programme for research, technological development and demonstration under grant agreement No 603946 (Health and Environment-wide Associations based on Large population Surveys, HEALS).

---

### The Study on the Relationship Between Mercury Exposure and Child Birth Defects

**Unursaikhan Surenjav (1), Baatartsol Dayanjav (1), Davaadorj Rendoo (1), Ichinkhorloo Bonduush (1), Govigerel Bayarsaikhan (1), Oyunchimeg Unshigbayar (2)**

(1) Public Health Institute, Ministry of Health, Ulaanbaatar, Mongolia
(2) National Center for Maternal and Child Health, Ulaanbaatar, Mongolia

Corresponding author:
Surenjav, Unursaikhan
Ulaanbaatar, Mongolia
unursaikhan_suren@yahoo.com

### *Background*

Due to lack of knowledge of the negative health impacts of mercury, illegal gold miners still using mercury for gold extraction activities. Mercury causes not only environmental pollution, but also effects human health. According to children’s health status statistics from 2006 to 2011 in South Gobi province, there have been 26 children born with birth defects. This number has a tendency to increase each year.

### *Methods*

The Scientific Review Board of the Public Health Institute has reviewed the methodology of this study and the Ethical Review Board of Ministry of Health gave permission to conduct the study. In order to find a connection with the causes of these defects and the parents’ work environments, we implemented a human bio monitoring survey of mercury exposures among children 0-5 years of age who were living in a mining area. A total of 56 children participated in this study, 22 boys and 34 girls. We performed pediatric clinical examinations for the all of the participants.

*Results*

We found that 52.9% were normal, 11.8% had rachitis, 7.8% were hypo oxygenated, and 6% had weight loss. We examined the mercury content in hair, urine and blood. The average mercury in participants' urine was 0.215 µg/l (confidence interval (CI) 95% 0.045-0.477), mercury in the root of the hair was 0.075 µg/g (CI 95% 0.042-0.108), mercury in the end of the hair was 0.111 µg/g (CI 95% 0.011 - 0.232), and mercury in the blood was 1.401 µg/l (CI 95% 1.179-1.623).

*Conclusions*

The results of mercury analysis were compared to the threshold of the Human Bio monitoring I limit. It concluded that no statistically significant differences were observed between mercury levels in biological samples of children and their parents' work related to gold mining with mercury. Parents working condition of the children involved in the study was not found to have an influence on children's health status (p=0.130, p=0.619).

---

### Noise Prevention in Kindergarten

**Katharina Takacs**

**Prevention Department, Austrian Workers' Compensation Board, Vienna, Austria**

**Corresponding author:**
**Takacs, Katharina**
**Vienna, Austria**
**Katharina.takacs@auva.at**

*Background*

One of the most important and underestimated challenges is high noise exposure in kindergarten. More than 50 percent of teachers complain about unfavourable noise conditions in child development facilities. Constant noise exposure affects not only attention performance and the development of language; it may also cause psychosomatic complaints or higher stress perception. Furthermore, legislative authorities, architects and teachers see noise pollution as an inevitable fact and do not consider noise protection or current needs, like children's exercise, when building or renovating premises, for example. Our priority intention is to introduce, develop and evaluate noise reducing interventions, which are easily to apply and have immediate effects.

*Methods*

We carried out a 10-month long longitudinal case-study in a kindergarten of high noise exposure, which had been selected according to a set of criteria. Our selection criteria were poor structural technical conditions, at least two groups with similar floorplans, number and age of children. Two similar groups were considered, one intervention group (IG) and one control group (CG) without any interventions.
In the presented project qualitative and also explorative – interpretative – methods were used (open-nonparticipating observation, interviews, survey tools, quantitative determination of individual sound levels (n=12) and also reverberation time of the rooms), because an authentic and natural setting was of great interest. In addition there were two observers in each group, observing and documenting near misses, conflict rate and also the daily routine respective of confounding variables, for example children shouting into their dosimeters. After the first measurement, all involved people developed a specific action plan for noise prevention. After the intervention implementation, a second measurement was taken.

### *Results*

The evaluation was carried out by comparing inter- and intra-individual differences between noise exposure levels in both groups (IG and CG) before and after implementation of measures.
At the first survey IG showed an average sound level of 76,3 dB, and after the implementation the sound level reduced to 70.9 dB (compared to CG: 71.5-72.7). Hence, it can be concluded that the noise prevention measures were successful. If the analysis of the observed conflicts and near misses rate in both groups beyond the two times of measurements is considered, a reduction was also achieved. The difference between CG and IG suggests a correlation between conflicts (IG: 23-9/CG: 18-16) or near misses (IG: 14-0) and high noise exposure. As the baselines of both groups differed, we do not want to interpret without further research. Looking at the individual sound exposure levels of adults and children, it appears that children are more affected than teachers, which matches our observations.

### *Conclusions*

On basis of these results it can be assumed that high noise exposure seems to have a negative impact on kindergartens. Construction, organisational or educational measures are able to reduce stressful sound volume and as a consequence the conflict and near misses rate as well. Noise measurements and consulting interventions in kindergartens should be put into practice. A Noise Prevention Checklist ("Which interventions are the most useful at a specific kindergarten?") and guidance about noise preventions for kindergarten management and policy makers is needed. Further research and evaluation of technical, organisational and pedagogical interventions should be developed in order to make policy makers and other stakeholders, like architects, aware of noise problems and its negative impacts.

We acknowledge the assistance of Eva Ruppert-Pils and Wilhelm Wahler.

### Forests for Health with the Environmental Midwife

Esther Tobarra Sánchez (1,2), Almudena Pernas Barahona (1,2), Lizbeth Álvarez Gómez (1,2), Miguel Felipe Sánchez Sauco (1,2), Milagros García Aroca(4), Mercedes Armero Barranco (4), Antonia Mira Pérez (4), Concepción Martínez Romero (4), Bernarda Díaz Velázquez (4), Juan Jiménez Roset (5), Juan Madrigal de Torres (6), Juan Antonio Ortega-García (1,2)

(1) Pediatric Environmental Health Speciality Unit, University Hospital Virgen de la Arrixaca, Murcia, Spain
(2) Laboratory of Environment and Human Health, Instituto Murciano de Investigación Biosanitaria, Murcia, Spain
(3) University Hospital Virgen de la Arrixaca, Murcia, Spain
(4) Maternity, University Hospital Virgen de la Arrixaca, Murcia, Spain
(5) Regional Coordinator of Drugs, General Direction of Public Health and Addictions, Consejería Regional de Sanidad, Murcia, Spain
(6) Oficina de Impulso Socioeconómico del Medio Ambiente, Consejería Regional de Agua, Agricultura y Medio Ambiente, Murcia, Spain

Corresponding author:
Tobarra Sánchez, Esther
Murcia, Spain
almudena@pehsu.org

*Background*

Forests for Health (FFH) is a project within the program "Nacer sin OH" (Births without OH), promoted by the Pediatric Environmental Health Speciality Unit of the Clinical University Hospital Virgen of Arrixaca (Murcia, Spain). This project intends that each child born and raised will be linked with a tree: incorporating a forest within each of the maternity hospitals of the Region of Murcia (Spain), creating healthy environments as protective factors during infancy, thus building health equity and promoting the enjoyment of natural heritage and regional biodiversity.

*Objective*

To foster the linkage of pregnant partners with nature and their future children from birth through adolescence, stimulating contact with nature as a preventive and therapeutic tool to achieve health, wellness and quality of life.

*Methods*

The environmental health midwife and various maternity healthcare professionals collaborate, giving the family a pine seedling, linked with the birth of a child, and an information package containing educational and prevention materials in line with the goals of the program. Subsequently they contact and invite parents to the replanting day, with the aim of creating forests for health.

*Results*

From 2015 to 2016, approximately 2000 pine seedlings were given to families. There have been 3,637

newborns. Approximately 450 trees have been planted in the FFH, with 4,694 tons $CO_2$/year absorbed.

### Conclusions

As health professionals, midwives are vital in accompanying pregnant couples and influencing the stages of preconception, pregnancy, childbirth, postpartum, throughout the cycle of sexual and reproductive health. The simplicity of the project allows it to be feasible and transferable for families, especially during such a receptive period.

### Impact of Prenatal Exposure to Environmental Pollutants on Autism Spectrum Disorder: A Case Report

Esther Tobarra Sánchez, Isabel Peraita Costa, Alberto Cárceles-Álvarez, Mayra Orozco Llamas, Lizbeth Álvarez, Juan Antonio Ortega-García

Pediatric Environmental Health Specialty Unit, University Hospital Virgen de la Arrixaca, Murcia, Spain

Corresponding author:
Tobarra Sánchez, Esther
Murcia, Spain
esthertob@gmail.com

### Background

Autism spectrum disorder has a high prevalence. This disease remains a complex issue and each patient is an etiological challenge. Hundreds of mutations in all chromosomes have been identified related to autism. In fetal life, brain development is exquisitely susceptible to exposure to toxic chemicals present in the environment. This report presents a case of a patient with a somatic mosaicism related to parental exposure to chemicals during early pregnancy.

### Methods

We examined the case of an 8-year-old boy diagnosed with pervasive developmental and attention deficit and hyperactivity disorder. Microarray hybridization identified a duplication of a segment of chromosome 19 from band p12 to q13.12. He was the first child of healthy non consanguineous parents. The pregnancy was achieved naturally and there was no medication or drug intake. Both parents work as operators in a chemical plant. The mother stopped working at 4 weeks gestation. She had episodes of nausea and dizziness when purging fuel vapors.

### Results

The affected segment includes over 160 genes that have possible associations with autism. Using the National Center for Biotechnology Information and Medical Data Institute database, we identified the chemicals associated with these genes, and volatile organic compounds, and volatile organic compounds and metals were some of the most common mutagenic chemicals identified.

### Conclusions

A careful environmental history is needed to understand the etiology of Autism spectrum disorder. In this case we describe acute symptoms of intoxication with gases derived from fossil fuels around the time of gastrulation. These gases contain volatile organic compounds and metals that are classified as mutagenic and could be associated with the chromosomal duplication and the clinical picture present in the patient. Despite the limitations, there is evidence to support that environmental and genetic factors could converge in neurotoxicity.

---

### Neuropsychological Profile of an Adopted Patient with Fetal Alcohol Spectrum Disorder.

Esther Tobarra Sánchez (1), María Luisa Azurmendi Funes (1), Alberto Cárceles-Álvarez (1), Miguel Felipe Sánchez-Sauco (1), Ferrán Campillo i López (1), Diana Carolina Jaimes Vega (1), Isabel Saura Martínez (2), Lizbeth Álvarez-Gómez (3), Juan Antonio Ortega-García (1)

(1) Pediatric Environmental Health Speciality Unit. Pediatrics Service. Laboratory Environment and Human Health, IMIB, Clinical Universitary Hospital Virgen de la Arrixaca, Murcia, España.
(2) Clinical Universitary Hospital Santa Lucía, Cartagena (Murcia, España)
(3) Mount Sinai School Of Medicine, EEUU, New York, USA

Corresponding author:
Tobarra Sánchez, Esther
esthertob@gmail.com

### Background

Prenatal exposure to alcohol causes a variety of clinical manifestations known as fetal alcohol spectrum disorder (FASD), with neurodevelopmental effects, among others. Alcoholism is common among mothers of children adopted from Spanish institutions, especially from Eastern Europe.

### Methods

A 17-year old boy, born in Ukraine and adopted by a Spanish family at the age of two was referred to the Genetics Unit because of psychomotor delay and facial dysmorphia. He is the product of an unknown controlled pregnancy, and had a sister with psychomotor retardation. The presence of dysmorphic features was detected in infancy by the Medical Genetics Unit, as well as psychomotor and pondostatural growth retardation. At the age of 7 he developed inattention and hyperactivity, as well as anxiety and depressive symptoms. The child was evaluated by the Neuropediatrics and Psychiatry Unit. Subsequently, the child was evaluated at school and by the Environmental Pediatric Service.

### Results

He presented with behavioral problems and poor school performance, limited IQ and verbal reasoning capacity below average, as well as significant curricular gaps in grammatics, mathematics and languages and difficulties in social relationships. He did not show an adequate level of autonomy, all in the context of FASD. The child was diagnosed with FASD.

### *Conclusions*

Patients with FASD may have a normal or decreased IQ, from minimum to profound mental retardation. Executive functions are the most frequently affected. In addition, they usually have poor performance in behavioral inhibition and self-control, verbal and nonverbal fluency, organization and planning. Other manifestations include difficulties in the area of mathematics and social communication because of language disorders. Problems in socialization are influenced by understimulation, common in adopted children. Behavioral problems, substance use, sexual behavior and psychotic disorders are also common. Early diagnosis and intervention and psychopedagogical treatment seem to achieve an improvement in the neurodevelopment of these patients.

### Quantification of the Burden of Disease Due to Environmental Risk Factors in Children and Adolescents in Germany — the UKAGEP Project

**Myriam Tobollik, Dirk Wintermeyer, Tristan Fischer, Andreas Gies, Dietrich Plass**

**German Environment Agency, Department Environmental Hygiene, Berlin, Germany**

**Corresponding author:**
**Tobollik, Myriam**
**Berlin, Germany**
**myriam.tobollik@uba.de**

### *Background*

Environmental Burden of Disease (EBD) estimates are increasingly used by scientists to inform decision-makers about environmental risk factors causing ill-health. Currently, no up-to-date national estimates of the EBD are available for Germany. Children and adolescents represent a group vulnerable to the effects of environmental risk factors. Therefore, the research project UKAGEP aims to estimate the EBD of 16 risk factors for children aged between 3 and 17 years of age in Germany.

### *Methods*

The UKAGEP project uses the EBD methodology and its core measure, the disability-adjusted life year (DALY). Current exposure and human-biomonitoring data derived in the currently ongoing population-representative German Environmental Survey (GerES 2014-2017) are used to estimate the exposure of children in Germany. Combined with exposure-response functions (ERF) and data on the related health effects, DALYs will be estimated. The ERF will be derived from or updated by systematic literature reviews and meta-analyses. To take into account cumulative chronic effects, probabilistic modeling will be performed. Furthermore, it is also necessary to estimate the potential future burden, because many current exposures might affect health after a long lag-period.

*Results*

The concentration of most environmental hazards is expected to be rather low. However, some of the risk factors might still pose a considerable threat at population level, especially if both effects of mortality and morbidity are considered. Furthermore, the estimated future burden will show how current exposures will impact the health of children living with today's exposures in the upcoming years.

*Conclusions*

The UKAGEP study will present the EBD of a set of known hazards, but will also provide insights on newly emerging risk factors. It is also envisaged to strengthen the use of human-biomonitoring data and methods of probabilistic modelling in EBD assessments.

Financial support of the GerES by the German Federal Ministry for the Environment, Nature Conservation and Nuclear Safety and the German Federal Ministry of Education and Research, and financial support of the KiGGS study by the German Federal Ministry of Health is gratefully acknowledged. We also thank the Robert Koch Institute for its support in this collaborative study.

---

### What is the Environmental Burden of Disease? An Animated Video Gives an Answer

Myriam Tobollik (1), Theresa Tobollik (2), Nadine Steckling (3,4)

(1) German Environment Agency, Section Exposure Assessment and Environmental Health Indicators, Berlin, Germany
(2) Raia Productions, Berlin, Germany
(3) University Hospital Munich, WHO Collaborating Centre for Occupational Health, Institute and Outpatient Clinic for Occupational, Social and Environmental Medicine, Workgroup Pediatric Environmental Epidemiology, Munich, Germany
(4) Department of Public Health and Health Technology Assessment, University for Health Sciences, Medical Computer Science and Technology, Hall in Tirol, Austria

Corresponding author:
Tobollik, Myriam
Berlin, Germany
myriam.tobollik@uba.de

*Background*

The Environmental Burden of Disease (EBD) method is used to quantify disability-adjusted life years (DALYs) which can be attributed to environmental risk factors. Thereby, risk factors of major concern for population health can be identified. DALYs are not easily understandable, which might prevent their use in risk communication to the general public as well as in political decision-making.

### Methods

An animated video was produced to explain the EBD method and its possible application fields to the interested general population, scientists from other research fields and policy makers. A vivid example, outdoor air pollution, which affects everyone's health, was chosen to illustrate the quantification steps. Technical terms are used and explained in common language.

### Results

The 8-minute video describes the DALY as a summary measure of population health which consists of years of life lost due to premature mortality and years lived with disability. Likewise, the attribution of DALYs to the risk factor outdoor air pollution is illustrated. The video is freely available on YouTube in German and English (https://www.youtube.com/watch?v=Inentz31Kqk).

### Conclusions

The video is designed to be understandable to a broad audience by using a balance between simplification and scientific correctness. It gives a short but comprehensive explanation of the complex EBD method and is therefore an attempt to break out of the scientific ivory tower.

---

### WHO's Work on Early Life Exposures and Future Health — Prevention for Future Generations

Emiko Todaka

World Health Organization, Geneva, Switzerland

Corresponding author:
Todaka, Emiko
Geneva, Switzerland
todakae@who.int

In 2012, 38 million people lost their lives due to non-communicable diseases (NCDs), and three-quarters of these deaths occurred in low and middle income countries. NCDs include obesity/diabetes, cardiovascular diseases, respiratory diseases, cancers, allergies and mental illnesses. Recently, the number of children with NCDs has increased, and this is cause for serious concern.

In the United Nation's (UN) Sustainable Development Goals (SDGs), target 3.4 aims to reduce by one third premature mortality from NCDs through prevention and treatment and promote mental health and well-being by 2030; target 3.9 aims to reduce the number of deaths and illnesses from hazardous chemicals and air, water and soil pollution and contamination. Although according to the Organisation for Economic Co-operation and Development (OECD), chemical production is increasing rapidly in the world, many chemical exposures could potentially be reduced or removed through environmental management. These chemicals include pesticides, asbestos, various other household and occupational chemicals, ambient and household air pollution, second-hand tobacco smoke, lead and arsenic.

In June 2016, an international expert meeting on “Avoidable Early Environmental Exposure” was convened by the World Health Organization (WHO) in Geneva. Experts from various fields such as maternal/children’s health, developmental origin of health and disease, epigenetic effects, cohort study, and communication participated. The meeting discussed the current situation of early life exposure to chemicals, possible interventions to reduce the risks to children, and effective advocacy among health care professionals, policy makers, and civil society. A roadmap of action for implementing interventions is now being developed. The outcomes of the meeting results will be submitted for publication in a scientific journal.

### Neurodevelopment, Low Level Mercury Exposure and Genetic Polymorphisms in a Birth Cohort From Slovenia and Croatia

Janja Snoj Tratnik (1,7), Ingrid Falnoga (1), Ajda Trdin (1,7), Darja Mazej (1), Alenka Sešek Briški (3), Joško Osredkar (3), Mladen Krsnik (3), Alfred B. Kobal (2), David Neubauer (3), Jana Kodrič(3), Staša Stropnik (3), David Gosar (3), Janja Marc (4), Igor Prpić(6), Zdravko Špirić(5), Milena Horvat (1,7)

(1) Department of Environmental Sciences, Jožef Stefan Institute, Ljubljana, Slovenia
(2) Ex-Department of Occupational Health, Idrija Mercury Mine, Idrija, Slovenia,
(3) University in Ljubljana, University Medical Centre Ljubljana, Slovenia,
(4) University in Ljubljana, Faculty of Pharmacy, Slovenia
(5) Oikon, Institute of Applied Ecology, Zagreb Croatia
(6) Medical Faculty of Rijeka, Department of Pediatrics, University of Rijeka, Rijeka, Croatia
(7) Jožef Stefan’s International Postgraduate School, Ljubljana, Slovenia

Corresponding author:
Tratnik, Janja Snoj
Ljubljana, Slovenia
janja.tratnik@ijs.si

### *Background*

The aim of the present study was to evaluate association between prenatal exposure to mercury and the neurodevelopment of children, taking into account relevant confounders and some relevant genetic polymorphisms. We examined apolipoprotein E (*Apoe*) and glutathione transferase (GSTM1 and GSTT1), the genes that are hypothetically involved in elimination of mercury from the body, and *Apoe* in neurodevelopment.

### *Methods*

The study population was comprised of 601 mother-child pairs recruited from the central Slovenia region and 243 from Rijeka, on the Croatian coast of the northern Adriatic. Determination of total mercury in cord blood, neurodevelopment assessment using Bayley Scales of Infant and Toddler Development, Third Edition (Bayley III) at 18 months of age and genotyping was done in 361 children; 237 of them were from Slovenia and 124 from Croatia. The National Ethics Committees of Slovenia and Croatia approved this study.

### Results

The results showed a negative association between low-to-moderate mercury exposure levels and cognitive outcomes and with fine motor scores at 18 months of age. The observed decrease in cognitive function was significant only in children carrying at least one *Apoe* ε4, while the decrease in fine motor score was independent of the *Apoe* genotype. Adjusting for selenium and lead revealed positive association between selenium and language and negative between lead and motor function, but only in the subgroup of children not carrying the ε4 allele. The GSTT1 genotype revealed positive association between cord blood selenium and cognition in children without gene deletion ($\beta=11.8$, $p=0.008$), but not in children with GSTT1 deletion ($\beta=-15.1$, $p=0.128$).

### Conclusions

The present study indicates that even low-to-moderate mercury exposure in children with normal neurodevelopmental outcome can be associated with lower cognitive and fine motor Bayley III scores and that stratifying for specific genotypes and accounting for beneficial and other potentially neurotoxic substances is crucial in assessing such associations.

---

### Early Life Exposure to Organochlorine Pesticides and Behavioral Development in Children

Ilona Quaak (1), Marijke de Cock (1), Eliška Echová (2), Anton Kocan (2), Michiel de Boer (3), Pim Leonards (4), Margot van de Bor (1)

(1) Department of Health and Life Sciences, Faculty of Earth and Life Sciences, Vrije University, Amsterdam, the Netherlands
(2) Research Centre for Toxic Compounds in the Environment, Masaryk University, Faculty of Science, Brno, Czech Republic
(3) Department of Health Sciences, Faculty of Earth and Life Sciences, Vrije University, Amsterdam, the Netherlands
(4) Institute for Environmental Studies, Faculty of Earth and Life Sciences, Vrije University, Amsterdam, the Netherlands

Corresponding author:
van de Bor, Margot
Amsterdam, the Netherlands
margot.vande.bor@vu.nl

### Background

Although organochlorine pesticides were banned in the West decades ago, bioaccumulation in younger generations still occurs. Exposure to organochlorine pesticides may be related to adverse effects on behavioral development. Exposure during early life may be particularly harmful, as rapid structural and functional changes take place. The aim of this study is to explore the association between early life exposure to organochlorine pesticides and behavioral development at 18 months.

*Methods*
Data from 59 mother-child pairs from LINC, a prospective birth cohort in the Netherlands, were used. The targeted compounds were the sum of polychlorinated biphenyls (ΣPCBs), hexachlorobenzene (HCB), the sum of hexachlorocyclohexane isomers (ΣHCH), heptachlor epoxide, and dieldrin; all determined in breast milk. The scales 'Attention Deficit Hyperactivity Disorder' (ADHD) and 'Externalizing Behavior' of the Child Behavior Checklist 1.5-5 were used for the behavioral assessment. Whole population and sex-stratified regression analyses were performed, in which exposure levels were classified in tertiles (T1, T2 and T3). A family history of ADHD, educational level, smoking, and alcohol or illicit drug use during pregnancy were included as confounders. Ethical approval was granted by the Vrije University Medical Center.

*Results*
Children exposed to the highest levels of ΣHCH presented higher scores on the ADHD-scale of the CBCL. After stratification for sex, significant associations were only found for girls. Positive associations were found between early life exposure to the highest levels of ΣPCBs, ΣHCH, dieldrin, and T2 levels of HCB; and ADHD symptoms. Higher scores were found on the Externalizing Behavior scale of the CBCL after exposure to dieldrin (T3) and ΣPCBs (T2 en T3).

*Conclusions*
Significant associations were found between early life exposure to organochlorine compounds and behavioral development at the age of 18 months. Associations were sex-specific (in girls only). Results should be seen as exploratory, as the sample size was small.

---

### Prenatal Exposure to Environmental Chemicals: Associations with Neurotransmitter Levels in Cord Blood

Marijke de Cock (1), Ilona Quaak (1), Jeroen Dudok (1), Raluca I. van Staden (2,3), Jacobus F. van Staden (2,3), Margot van de Bor (4)

(1) Institute for Environmental Studies, Faculty of Earth and Life Sciences, Vrije University, Amsterdam, the Netherlands
(2) Process Analytical Technology Laboratory, National Institute for Research and Development in Electrochemistry and Condensed Matter, Timisoara, Romania
(3) Laboratory of Electrochemistry, National Institute for Research and Development in Electrochemistry and Condensed Matter, Bucharest, Romania
(4) Department of Health and Life Sciences, Faculty of Earth and Life Sciences, Vrije University, Amsterdam, the Netherlands

Corresponding author:
van de Bor, Margot
Amsterdam, the Netherlands
margot.vande.bor@vu.nl

### Background

Neurodevelopmental disorders such as attention deficit hyperactivity disorder and autism have been associated with exposure to environmental chemicals, including dioxins and organochlorine pesticides. Effects have been observed, particularly in relation to exposure in early life. Mechanistic pathways, such as relations between chemical exposure and neurotransmitter levels, have, however, been studied less frequently. Our objective was to study the effect of early life exposure to environmental chemicals on neurotransmitter levels in cord blood.

### Methods

Data were used from 63 mother-child pairs from the LINC study, a prospective birth cohort in the Netherlands. Cord plasma was collected at birth and was used to determine both levels of environmental chemicals and neurotransmitters. Exposure to various compounds was determined, including phthalates (metabolites of di-2-ethylhexyl phthalate [DEHP]), perfluorinated alkyl acids (perfluorooctanesulfonic acid [PFOS], and perfluorooctanoic acid [PFOA]), and brominated flame retardants. The neurotransmitters included dopamine, epinephrine, norepinephrine, acetylcholine, serotonin, gamma-amino butyric acid (GABA), and glutamate. Univariate regression models were composed for each compound in tertiles, and each neurotransmitter. Ethical approval was granted by the Vrije University Medical Center.

### Results

An overall significant effect of PFOA exposure on GABA was observed ($p = 0.049$). It appeared to be that higher levels were observed in the second tertile compared to the first, and lower levels in the third tertile compared to the first. Furthermore significant overall effects were observed for MEHHP (acetylcholine, $p = 0.039$), and MEOHP (epinephrine, $p = 0.048$). For mono(2-ethyl-5-hydroxyhexyl) phthalate (MEHHP), lower levels of acetylcholine were observed in the highest exposure tertile, whereas for mono(2-ethyl-5-carboxypentyl) phthalate (MEOHP) higher levels of epinephrine were observed in the second exposure tertile.

### Conclusions

Prenatal exposure to PFOA and DEHP was associated with levels of GABA, acetylcholine, and epinephrine. Dose-response effects were non-linear. Whether these changes in neurotransmitter levels in cord blood are related to abnormal behavioral development needs to be further explored.

### Maternal Exposure to Particulate Matter and Birth Weight in Dutch Children

Marijke de Cock (1), Ilona Quaak (1), Laura P. den Ouden (2), Margot van de Bor (3)

(1) Institute for Environmental Studies, Faculty of Earth and Life Sciences, Vrije University, Amsterdam, the Netherlands
(2) Erasmus University Rotterdam, Rotterdam, the Netherlands
(3) Department of Health and Life Sciences, Faculty of Earth and Life Sciences, Vrije University, Amsterdam, the Netherlands

Corresponding author:
van de Bor, Margot
Amsterdam, the Netherlands
margot.vande.bor@vu.nl

*Background*

Maternal exposure to particulate matter (PM) may cause decreased transplacental oxygen and nutrient transport to the fetus, potentially resulting in a lower birth weight (BW). Results of observational studies have, however, been inconsistent and because of the potential health implications, further research is needed to confirm the relation between maternal exposure to PM and BW.

*Objective*

To investigate the association between maternal residential exposure to fine and coarse particulate matter ($PM_{2.5}$ and $PM_{10}$) and BW.

*Methods*

Data were used from the LINC study, a prospective birth cohort study in the Netherlands (n = 223). Individual exposure estimates to $PM_{2.5}$ and $PM_{10}$ at each participant's home address were assessed using a geographical air quality map. Data on BW, gestational age and maternal anthropometry were obtained from the midwives, and data on parental health, lifestyle and socio-demographics characteristics were collected by means of questionnaires. To estimate the impact of PM on BW as a continuous measure, linear multivariable models were used. Because the sample size, PM was converted into a dichotomous variable with the concentration groups <12 μg $PM_{2.5}/m^3$ (reference group) and 12 – 13 μg $PM_{2.5}/m^3$ for $PM_{2.5}$ and the concentration groups <19 μg $PM_{10}/m^3$ (reference group) and 19 – 20 μg $PM_{10}/m^3$ for $PM_{10}$. Ethical approval was granted by the Vrije University Medical Center.

*Results*

For 191 of the 223 children (85.7%), exposures were successfully estimated. The highest exposure level for $PM_{2.5}$ was 12-13 μg/m$^3$, to which 65.4% of participants were exposed. For $PM_{10}$, the highest exposure level was 19-21 μg/m$^3$, which was observed for 52.4% of households. Only exposure to a higher concentration of $PM_{2.5}$ resulted in a statistically significant negative effect on BW (p=0.027). After adjusting for confounders, a decrease in mean BW was found for $PM_{2.5}$, with a change of -142.8 g (95% CI, -269.1 to -16.4).

### Conclusions

Maternal exposure to higher levels of $PM_{2.5}$ was associated with a lower BW. The implications for public health in the Netherlands are important, as exposure levels in this study are common.

### Polycyclic Aromatic Hydrocarbons from Traffic Exhausts in Primary Schools and Cognitive Development of Children

Barend L. Van Drooge (1), Joan O. Grimalt (1), Mikel Esnaola, (2,3,4), Ioar Rivas (6), Mar Alvarez-Pedrerol (2,3,4), Joan Forns (2,3,4), Payam Dadvand (2,3,4), Xavier Basagana (2,3,4), Xavier Querol (1), Jordi Sunyer (2,3,4,5)

(1) Institute of Environmental Assessment and Water Research, Spanish National Research Council, Barcelona, Spain
(2) Barcelona Institute for Global Health, Centre for Research in Environmental Epidemiology, Barcelona, Spain
(3) Pompeu Fabra University, Barcelona, Spain
(4) Centro de Investigación Biomédica en Red de Epidemiología y Salud Pública, Madrid, Spain
(5) IInstitut Hospital del Mar d'Investigacions Mèdiques-Parc de Salut Mar, Barcelona, Spain
(6) Department of Civil and Environmental Engineering, University of Surrey, Surrey, United Kingdom

Corresponding author:
Van Drooge, Barend L.
Barcelona, Spain
barend.vandrooge@idaea.csic.es

### Background

Traffic air pollution is associated with slower cognitive function growth in schoolchildren. Polycyclic aromatic hydrocarbons (PAHs) impair brain development in animal studies. Here, we assess the association between PAHs in urban schools (n=39) and the cognitive development in children (n=2,897) from 2nd to 4th grade (7-10 years).

### Methods

Cognitive development was assessed through 12-month change in working memory and attention. All parents or guardians signed the informed consent form approved by the Clinical Research Ethical Committee of the IInstitut Hospital del Mar d'Investigacions Mèdiques-Parc de Salut MAR, Barcelona, Spain.

Schools were sampled on particulate matter 2.5 ($PM_{2.5}$) indoor (classroom) and outdoor (playground) twice in 2012 during one-week periods. One fourth of each $PM_{2.5}$ filter was used for PAH analysis by gas chromatography–mass spectrometry. Furthermore, organic carbon, elemental carbon, nitrogen dioxide and $PM_{2.5}$ from traffic origin were measured.

### Results

Indoor PAH levels were similar to outdoor levels (ratio 0.9), except for the more volatile particulate bounded PAHs, such as benz[a]nthracene and chrysene with higher indoor levels (ratio 1.5). Higher PAH concentrations were observed in schools from the urban centre. BaP, the only compound with an established annual limit concentration (1000 pg/m$^3$), ranged from 22 to 343 pg/m$^3$ indoors and from 17 to 301 pg/m$^3$ outdoors. Correlations between outdoor PAHs and EC were high (0.66<r<0.79) while indoor correlations had a wider range (0.54<r<0.84) and were lower for benz[a]anthracene (r=0.62) and chrysene (r=0.54). A similar pattern was seen for NO2 and traffic $PM_{2.5}$.

Outdoor PAHs were generated by traffic inputs, while indoor PAHs reflected older mixtures. The more volatile PAHs were under the influence of gas-particle partitioning in combination with high indoor organic carbon. Children attending schools with higher PAH levels had worse working memory and an increase in inattentiveness, which was statistically significant for the more volatile PAHs and benzo[ghi] perylene. The exact mechanism by which PAHs affect the developing brain remains unclear, however, it is likely to be mediated by inducing inflammation, oxidative stress, and vascular injury.

### *Conclusions*

This study points towards the role of motorized vehicle exhausts in air pollution in schools on cognitive development in a modern urban area. Although stronger associations to effects were observed in the outdoor PAH distribution, and thus, fresh vehicle exhaust, the effects were also reflected in the indoor air, which receives outdoor contamination.

### *Acknowledgements*

The research leading to these results has received funding from Fundació Obra Social "La Caixa" and the European Research Council under the ERC Grant Agreement number 268479 – the BREATHE project.

---

### Considerations for Policies Design to Promote Drinking Water Consumption in Mexican Children and Adolescents: Santa Fe, CDMX

**Arely Vergara-Castañeda, María de los Angeles Peña-Farfán, Donovan Raúl Ríos-Hernández, César Jesús Sandoval-García, Laura Martino-Roaro**

**Research Group "Food and Health," Faculty of Chemistry, La Salle University, D.F., Mexico**

**Corresponding author:**
**Vergara-Castañeda, Arely**
**Mexico City, Mexico**
**arely.vergara@gmail.com**

### *Background*

Water is the major constituent of the human body and drinking water is the most important source to fulfil its needs. There are several choices in water provision (e.g., bottled water consumption, home-based water treatment or installation of water storage facilities) which can be adapted to the existing water supply system in urban communities where we cannot ensure access to potable water. According to the World Health Organization, not only physical environmental, biological or chemical factors affect human health, but also environmental and psychosocial factors. In this sense, factors such as little or no availability of drinking water can contribute to low water consumption. The aim of this analysis is to emphasize the need for multicomponent strategies (access, social campaigns, social empowerment and psychosocial factors) to attain the recommended levels of water consumption in Mexican children and adolescents living in Santa Fe, CDMX.

*Methods*

This study examines the feasibility of implementing strategies aimed at increasing water consumption in children and adolescents as stated in the National Food Health Agreement (Acuerdo Nacional de Salud Alimentaria) in 2010 according to the socio-demographic conditions reported for a marginalized urban area in CDMX, Mexico. We also describe various other factors that could play a decisive role in the trend of tap water consumption in this specific population, putting them at risk for not accomplishing the recommendations and discuss a direction for developing specific child welfare policy for vulnerable groups.

*Results*

In Mexico, one sixth of the population has no access to drinking water (1100 million) and almost 40% lack basic sanitation. These figures are worse in marginalized vulnerable urban communities as Santa Fe in CDMX where only 18% of children and adolescents reach water consumption recommendations. As this population spends most of their time in schools and at home, these places should been considered key places to promote and ensure water consumption. Current polices to promote drinking water are focused only on the installation of water fountains in schools (some reports estimated that 55% of public elementary schools have this service) without considering availability at home and it has been recognized that confidence about the quality of water filters affects water consumption. Psychosocial factors such as intent, knowledge, beliefs, attitudes, subjective norms and self-efficacy, which are associated factors with low water consumption, have not been considered as part of environmental modifications.

*Conclusions*

Policies aimed to increase water consumption in children and adolescents should involve not only multicomponent environmental modifications aimed to ensure access to drinking water not only in schools, but also consider broader psychosocial factors.

### Occupational Injuries Among Child Labourers: Results From a Cross-Sectional Study in Peru

Tobias Weinmann (1,2), Cornelia Schlick (2,3), Manuela Joachin (4), Leonardo Briceño (5), Daniel Moraga (6), Katja Radon (7)

(1) Occupational and Environmental Epidemiology and NetTeaching Unit, Institute and Outpatient Clinic for Occupational, Social and Environmental Medicine, University Hospital of Munich, Munich, Germany
(2) German Centre for Lung Research, Munich, Germany
(3) Department of Neurology, Ludwig-Maximillian's-University of Munich, Munich, Germany
(4) Centro Medico Espinar Sociedad de Responsabilidad Limitada, Cuzco, Perú
(5) Department of Public Health, Universidad del Rosario, Bogota, Colombia
(6) Universidad Iberoamericana de Ciencias y Tecnologia, Santiago, Chile
(7) Department of Occupational Health and Environmental Medicine, Ludwig-Maximillian's University of Munich, Munich, Germany

Corresponding author:
Weinmann, Tobias
Munich, Germany
tobias.weinmann@med.lmu.de

*Background*

Although there is a considerable number of children involved in child labour worldwide, data on occupational hazards and injuries is limited. Thus, the objective of this study was to investigate the lifetime prevalence of and risk factors for occupational injuries in child labourers in Peru.

*Methods*

Between August and December 2010, a cross-sectional study was conducted at five public night schools in Cusco Province. 375 students (response 91.5%) aged 10 to 17 years completed an interview-based questionnaire on socio-demographic factors (age, sex, place of birth, education, residential status, minority status, health care coverage), work-related factors (income, working days per week, occupational sector) as well as lifetime prevalence of occupational injuries. Multiple logistic regression analyses were performed to estimate risk factors for different types of occupational injuries. Ethical approval was granted by the Medical Faculty of the University of Munich and the Ministerio de la Mujer y Desarrollo Social (Ministry of Women und Social Development) in Peru.

*Results*

We found falls (11%), car accidents (9%) and physical violence (3%) were common causes of injuries in this population. Severe injuries (fractures, luxation or amputations) were reported by three percent of the study sample. Participants with high daily income (≥20 PEN, ~15 USD) showed increased odds for falls (odds ratio 2.8; 95% confidence interval 1.2-6.5) and physical violence at work (odds ratio 12.1; 95% confidence interval 1.3-116.0). Children working in the service sector were at higher risk of injuries caused by car accidents (odds ratio 4.2; 95% confidence interval 1.2-15.3).

### Conclusions

Occupational accidents among child workers attending public night schools are common in Cusco with a lifetime prevalence of three percent for severe injuries. High income seems to convince child labourers to accept poor working conditions.

---

### Air Pollutants Exposure and Functional Repair Activity In Placenta

Nurulshyha Md. Yatim (1), Geoffrey P. Margison (1), Edward Johnstone (2), Andrew C. Povey (1)

(1) Centre for Occupational and Environmental Health, Centre for Epidemiology, Institute of Population Health, University of Manchester, Manchester, England, UK
(2) Maternal and Fetal Health Research Centre, St. Mary's Hospital, Manchester, England, UK

Corresponding author:
Yatim, Nurulshyha Md.
Manchester, England, UK
nurulshyha.mdyatim@postgrad.manchester.ac.uk

### Background

Maternal exposure to air pollutants can affect fetal development and cause adverse birth outcomes. Nitrogen oxides (NOx) can react with amines to generate toxic alkylating agents (AAs), and their biological effects are mediated, in part, by the formation of DNA damage including the promutagenic and toxic, O6-methylguanine. O6-methylguanine methyl transferase (MGMT) removes O6-alkylguanine adducts by transferring the alkyl group from the O6 position to the cysteine residue within its active site. The aim of this study is to quantify MGMT functional repair activity in placentas and to determine its association with air pollutant exposure over various time windows during pregnancy.

### Methods

Tissue samples from the middle, cord and peripheral areas of the normal placentas were collected from 10 mothers, who lived in Greater Manchester, and stored at -80°C until processed. MGMT repair activity was quantified in cell-free sonicates of 100-120 mg tissue using a standard radioistotopic methyl transfer assay and protein levels were quantified by a Bradford assay. Postcodes of the mothers were geocoded by ArcGIS and air pollutant data obtained from the Department for Environment, Food and Rural Affairs. Ethical approval was granted by the Central Manchester University Hospitals NHS Foundation Trust.

### Results

Placental MGMT activity (mean ± standard deviation) was 0.140 ± 0.022, 0.134 ± 0.014 and 0.124 ± 0.007 fmoles/µg for middle, cord and peripheral samples respectively and varied 4, 5 and 1.5 fold respectively. Level of pollutant exposure varied with mean nitrogen dioxide exposure was 16.2 $\mu g/m^3$, NOx 23.3 $\mu g/m^3$, $PM_{10}$ and $PM_{2.5}$ were 13.1$\mu g/m^3$ and 9.4$\mu g/m^3$ respectively. None of the pollutants were significantly associated with any time windows during the pregnancy ($p>0.05$).

*Conclusions*

This initial study indicates that the placenta contains functional MGMT activity that may affect susceptibility to alkylating agent exposure. The biological significance of variable placental MGMT activity remains to be characterized. An association between placental MGMT activity with pregnancy outcomes need to be further studied.

---

### Acquired Immune Deficiency syndrome Orphans in Sub-Saharan Africa: A systematic Review on Differences Between Rural and Urban Environments

Tara Rava Zolnikov (1,2)

(1) National University, San Diego, California, USA
(2) Shepherd's Village, Narok, Kenya

Corresponding author:
Zolnikov, Tara Rava
San Diego, California, USA
tarazolnikov@gmail.com

*Background*

Currently, there are more than 11 million acquired immune deficiency syndrome (AIDS)-affected orphans that suffer from various adverse effects, most of whom reside in sub-Saharan Africa. The difference between whether a child resides in a rural or urban environment can have a significant role in a child's education, health status and access to healthcare, and social or family relationships.

*Methods*

A systematic review was conducted in order to understand these environment-based differences on orphans directly affected by human immunodeficiency virus infection (HIV)/AIDS in sub-Saharan Africa. There were 233 sources used for this review; however, 164 manuscripts focused more on a general review of orphans within a rural or urban environment. Thus, after eliminating for various factors, 69 manuscripts were used, which focused primarily on the social aspect of orphans due to HIV/AIDS.

*Results*

Rural environments provided more family support, while urban environments generally had more resources available to orphans (e.g. school fees). Unfortunately, both rural and urban environments were found to be fairly non-supportive of orphans and their development. This systematic review found, in general, that orphans in both urban and rural environments continue to suffer from the iatrogenic effects of low parental support due to AIDS mortality.

*Conclusions*

These conclusions continue to support an increase in developmental support through school and social relationships specific to orphans throughout sub-Saharan Africa.

### Improved Relationships in Eastern Kenya from Water Interventions and Access to Water

Tara Rava Zolnikov (1,2), Elizabeth Blodgett Salafia (3)

(1) National University, San Diego, California, USA
(2) Shepherd's Village, Narok, Kenya
(3) Human Development and Family Science, North Dakota State University, North Dakota, USA

Corresponding author:
Zolnikov, Tara Rava
San Diego, California, USA
tarazolnikov@gmail.com

*Background*

Global health interventions focus on providing solutions to unaddressed public health issues in low- and middle-income countries. Providing nearby access to safe drinking water is a primary public health concern and a frequent public health intervention throughout sub-Saharan Africa; access to water eliminates long gathering and transport time and provides an individual with more personal time. Previous research has not reviewed the aspect of time and relationship changes after implemented water interventions.

*Method*

A qualitative phenomenological approach used 52 semistructured interviews to examine relationship experiences among primary water gatherers and their families after receiving nearby access to water. The study group consisted of men and women from approximately 7 years old to 75 years old. This study took place throughout the historically semiarid eastern region in Kitui, Kenya, where community members have been beneficiaries of various water interventions. Ethical approval was granted by both North Dakota State University and Moi University.

*Results*

Before the water intervention, family members experienced difficulties with irregular meal times and food availability, infrequent family conversations, irritation with lack of bathing and cleanliness, and general discontent without the availability of 1 able-bodied family member. Participants' experiences after implemented water interventions revealed enhanced relationships within household family units; additional personal time was gained and used to rebuild relationships. The newly established relationships included constructive discussions and conversations built around understanding and developing solutions to address economic or individual challenges encountered by the family.

*Conclusions*

This study's findings suggest that relationships significantly improved after households receive access to nearby water. Furthermore, this research provides evidence of an increased need for access to quality water for communities throughout the world to positively contribute to constructive family relationships.

### A Review on Managing and Treating Mercury Emissions in Small Artisanal Gold Mining

Tara Rava Zolnikov (1,2), Gavin Hilson (3)

(1) National University, San Diego, California, USA
(2) Shepherd's Village, Narok, Kenya
(3) Sustainability in Business, University of Surrey, Surrey, United Kingdom

Corresponding author:
Zolnikov, Tara Rava
San Diego, California, USA
tarazolnikov@gmail.com

*Background*

Small artisanal gold mining (SAGM) continues to be a pervasive public health issue worldwide, affecting miners as well as both children and women. While there are various problems associated with SAGM, mercury exposure is the primary hazard contributing to adverse health effects in exposed human populations.

*Methods*

A literature review was conducted in order to specifically assess the impact of current mercury interventions within the entire SAGM industry. To aid in this review, a resource pool of information on SAGM and mercury emissions, treatment and management of emissions, and interventions was assembled through a search conducted via multiple search engines.

*Results*

Mercury usage continues to be largely embedded in the handling, processing, and extraction techniques of SAGM; however, current interventions have been implemented worldwide to focus specifically on reducing or eliminating mercury emissions through education, processing centers, or mercury alternative techniques. Education focused on environment or health awareness or more specifically on alternative techniques. Processing centers offered areas of large-scale technology centers for small-scale miners. Alternative techniques included retorts, vat-leaching, magnets, direct smelting of gold, and borax. Results determined positive and negative outcomes associated with every intervention, thereby confirming the fact that a single solution managing and treating mercury currently does not exist.

*Conclusions*

Ultimately, the need for novel techniques addressing mercury emissions in SAGM needs to be pushed to the forefront of critical issues in public health in order to aid in developing positive change in the industry.
